# Synthesis and Docking Studies of Glycolipids Inspired by *Bacteroides fragilis* Lipid A

**DOI:** 10.3390/molecules30193927

**Published:** 2025-09-30

**Authors:** Davie Kenneth, Cristina Manuela Santi, Francesca Tanda, Alessia Izzo, Monica Civera, Giuseppe D’Orazio, Luigi Lay

**Affiliations:** 1 Department of Chemistry, Università degli Studi di Milano, Via C. Golgi 19, 20133 Milan, Italy; davie.kenneth@unimi.it (D.K.); cristina.santi@iit.it (C.M.S.); francesca.tanda@unimi.it (F.T.); alessia.izzo96@gmail.com (A.I.); monica.civera@unimi.it (M.C.); 2 Laboratory for Polymers and Biomaterials, Fondazione Istituto Italiano di Tecnologia, 16163 Genova, Italy

**Keywords:** lipid A, glycolipids, *Bacteroides fragilis*, immunomodulation, glycoderivatives, anti-inflammatory agents, molecular docking

## Abstract

*Bacteroides fragilis*, a prominent commensal of the human gut microbiota, plays a vital role in immune system regulation through its capsular polysaccharide A (PSA), which requires a glycolipid anchor structurally reminiscent of lipid A. While canonical *Escherichia coli* lipid A acts as a potent TLR4 agonist contributing to septic shock and inflammatory disorders, certain *B. fragilis*-derived glycolipids demonstrate antagonistic effects, offering potential as anti-inflammatory agents. In this study, we report the synthesis and preliminary computational evaluation of a library of glycolipids inspired by *B. fragilis* lipid A. Three lipid As, including a tetra-acylated 1-phosphoryl lipid A analog (Tetra C-1), were synthesized and assessed using molecular docking simulations targeting the human TLR4/MD-2 complex. Docking results reveal that Tetra C-1 exhibits more favorable antagonist binding characteristics compared to the well-studied TLR4 antagonist Eritoran. This work highlights a microbiota-informed strategy for the development of novel TLR4 antagonists, potentially enabling targeted modulation of innate immunity for therapeutic applications in inflammatory diseases and as vaccine adjuvants.

## 1. Introduction

The innate immune system is highly sensitive to microbial signatures of invaders, with lipopolysaccharides (LPS)—particularly their lipid A component—playing a pivotal role in triggering the immune response [1]. Lipid A, composed of a mono- or di-phosphorylated di-glucosamine core with multiple fatty acid chains, anchors LPS into the bacterial membrane and is largely responsible for the toxicity of Gram-negative bacteria [2]. As the bioactive core of LPS, bacterial lipid As play a dual/Janus-like role. On one side, they are vital immunostimulants, able to modulate humans’ immune response through their interaction with key components of the innate immune system named Toll-like receptors (TLRs). Some particular lipid As have been proven to have a low-toxicity profile, stimulating a mild activation of the innate immune system capable of boosting the immune response to co-administered antigens. On the other side, other lipid A structures lead to excessive immune reaction and, upon being lysed by the immune system, their fragments are released into the bloodstream, causing dysregulated inflammatory processes and endotoxin-related pathologies such as fever, diarrhea, and possible fatal endotoxic shock [3,4]. This paradox highlights the need for precise modulation of innate immunity and emphasizes the therapeutic potential of targeting Toll-like receptor 4 (TLR4)—the central sensor for LPS in several types of immune cells like monocytes, neutrophils, dendritic cells, and macrophages [5,6,7] as well as intestinal epithelial cells [8,9].

**Figure 1:**
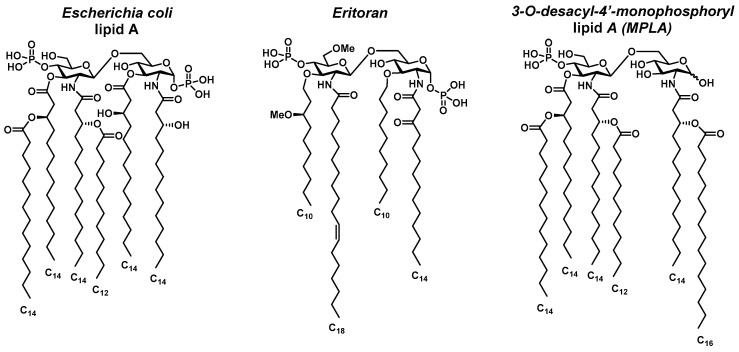
Structures of different lipid A from *E. coli*, Eritoran, and MPLA.

*Escherichia coli* lipid A (Figure 1), the reference structure of this family of glycolipids, strongly activates the TLR4/MD-2 (Myeloid Differentiation factor 2) complex, initiating a potent inflammatory cascade, which strictly depends on the lipid A/LPS structure and concentration [10]. Structure-activity relationship studies reveal that the endotoxicity of lipid A is influenced by its phosphorylation level and the number, length, and spatial arrangement of acyl chains [11]. Some over- or under-acylated lipid A variants behave as TLR4 antagonists, suggesting that structural modifications can provide lipid A with an immunomodulatory activity [12]. Although several antagonists have been developed, such as Eritoran (based on *Rhodobacter sphaeroides* lipid A, Figure 1) [13], clinical outcomes have been limited, reflecting the need for better-designed molecules with enhanced efficacy and safety profiles. Specific lipid A derivatives also hold promise in vaccine adjuvant development. Many subunit vaccines rely on adjuvants to improve immune response strength and duration, lower the required dosage, and enhance the generation of memory B cells [14]. Adjuvants can also boost immune responses and accelerate initial vaccine efficacy [15].

To uncouple the toxicity of lipid A from its immune-stimulating properties, Masihi et al. developed monophosphoryl lipid A (MPLA) in 1986 [16]. Derived from lipid A of *Salmonella minnesota* R595, MPLA was obtained by removing one acyl chain and one phosphate group, reducing toxicity by 100-fold while preserving immunostimulatory effects (Figure 1). MPLA acts as a TLR4 agonist, promoting dendritic cell maturation and Th1 CD4+ T cell polarization [17,18,19]. MPL^®^, an MPLA-based adjuvant, is the only TLR4 ligand approved for human vaccines and is included in Cervarix^®^ and Fendrix^®^ (by GSK, London, UK), targeting HPV and Hepatitis B, respectively [18].

A promising frontier in developing new and safer lipid A-based immunomodulatory agents lies in the exploration of commensal bacteria [20] and their unique glycolipid architectures—especially those with immunomodulatory rather than pro-inflammatory properties. The human immune system’s tolerance toward symbiotic bacteria makes them attractive candidates for novel, safe vaccine adjuvants. Among these, *Bacteroides fragilis*, a Gram-negative bacterium inhabiting the human gastrointestinal tract, has emerged as a model organism.

*Bacteroides fragilis* is known for its capsular polysaccharide A (PSA), which promotes immune tolerance and anti-inflammatory cytokine production, particularly IL-10, through interactions with dendritic cells and regulatory T cells [21,22,23]. In 2019, the Kasper group revealed that the lipid A moiety anchoring PSA plays a crucial immunomodulatory role [24]. The authors demonstrated that upon the glycolipid-mediated activation of the TLR1-TLR2 heterodimer and polysaccharide-based Dectin-1 engagement, PSA processing and its presentation to CD4+ T cells were induced, ultimately leading to production of IL-10, indicating an anti-inflammatory response which did not occur when PSA lacked its lipid anchor, hence highlighting the importance of the glycolipid moieties. By means of NMR and LC/MS-MS, the authors characterized *B. fragilis* lipid A as having a di-glucosamine backbone with variable acylation (C15–C17 chains) and phosphorylation patterns [24], along with tri- and tetra-acylated variants. Compared to *E. coli* lipid A, *B. fragilis* lipid A shows greater structural diversity, which may explain its distinct TLR2/TLR4-mediated immune responses [25].

**Figure 2:**
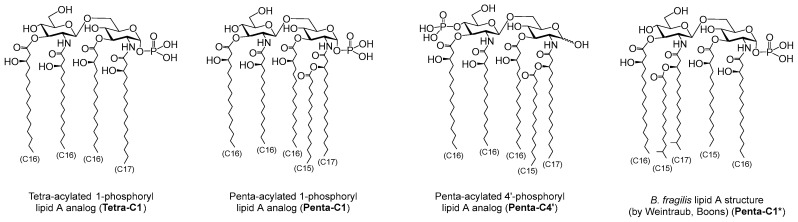
Structures of the lipid A analogues object of this work.

Interestingly, as early as 1989, Weintraub et al. proposed a lipid A structure for *B. fragilis*, characterized by a distinct pattern of fatty acid chains, including terminally-branched chains [26] (Penta-C1*, Figure 2), the same features that were later acknowledged in the more recent work of Kasper [24]. Furthermore, this structure was recently synthesized and immunologically evaluated [27]. The compound exhibited an antagonistic response towards the production of pro-inflammatory cytokines induced by *E. coli* lipid A, which is a natural ligand of the TLR4/MD-2 heterodimer [27].

Despite its biological relevance, the detailed structure of *B. fragilis* lipid A remains only partially understood. Due to gaps in structural characterization, our study is focused on the synthesis and computational evaluation of glycolipid compounds inspired by *B. fragilis* lipid A, considering the various structural hypotheses proposed to date.

In this work, we report the synthesis of three lipid A structures (**Tetra-C1**, **Penta-C1,** and **Penta-C4′**, Figure 2) along with preliminary molecular docking analysis to assess their binding affinity to the TLR4/MD-2 complex. The compounds panel included both newly synthesized structures and the one previously reported by Weintraub and Boons [26,27] (Penta-C1*, Figure 2). We evaluated the antagonistic potential of synthesized lipid A analogs against the TLR4/MD-2 complex through in silico methods, comparing their inhibitory efficacy to that of the known antagonist Eritoran, and aimed to highlight the distinctive functional properties of *Bacteroides fragilis*-derived lipid A to support its further biological validation as a promising immunomodulatory candidate.

## 2. Results and Discussion

### 2.1. Library Design

Inspired by literature, particularly the studies conducted in 2019 [24], in 1989 [26], and more recently in 2024 [27]—we designed a set of lipid A analogues featuring three novel structures: **Tetra-C1**, **Penta-C1**, and **Penta-C4′** (Figure 2). These compounds contain either four or five fatty acid chains, including C16 and C17 chains bearing 3-hydroxy groups with (*R*)-configuration. Each chain is directly attached to the hydroxyl or amino functionalities of the di-glucosamine disaccharide backbone. In both **Penta-C1** and **Penta-C4′**, the 3-hydroxy group of the C17 chain connected to the amino group of the reducing-end glucose is further acylated with a linear C15 fatty acid. Phosphorylation occurs at the anomeric position of the reducing-end glucose in **Tetra-C1** and **Penta-C1**, whereas in **Penta-C4′** the phosphate group is positioned at C4′.

The library also includes the lipid A analog Penta-C1*, originally proposed by Weintraub [26] and subsequently synthesized and evaluated by Boons in 2024 [27]. This compound exhibits a distinct fatty acid profile, including C15 and C17 branched chains, setting it apart from the newly designed structures. All three novel analogues were synthesized from a common disaccharide intermediate. The complete set—comprising both the newly developed and the previously proposed structures—was subjected to molecular docking simulations with the TLR4/MD-2 heterodimer to evaluate binding affinities. The results were compared to reference ligands, including Eritoran, a well-known TLR4 antagonist [13,28], and lipid IVa from *E. coli*, a canonical TLR4 antagonist [29,30,31].

### 2.2. Synthesis of Target Compounds-General Approach

**Scheme 1:**
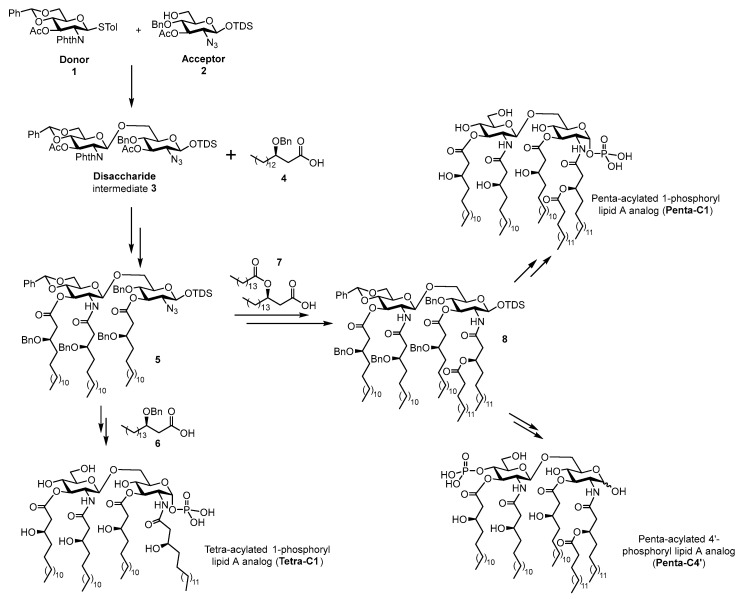
General scheme for the synthesis of lipid A compounds **Tetra-C1**, **Penta-C1,** and **Penta-C4′**.

The synthesis of the lipid A analogues (**Tetra-C1**, **Penta-C1**, and **Penta-C4′**) relied on two key structural components: a protected di-glucosamine disaccharide (**3**) and three fatty acid chains (**4**, **6**, and **7**), derived from commercially available natural fatty acids. In our approach (Scheme 1), the di-glucosamine disaccharide **3**, obtained via glycosylation of donor **1** and acceptor **2**, served as the carbohydrate scaffold. Protective groups on the donor and acceptor were selected to enable stereoselective β-glycosylation and facilitate selective deprotection at the sites intended for functionalization with fatty acid chains. To introduce 16-carbon chains at positions 3, 2′, and 3′ of the disaccharide, phthalimide and acetyl groups were employed to protect the amine at 2′ and hydroxy groups at 3 and 3′, respectively, due to their susceptibility to base-mediated cleavage. In order to install the fatty acid chain **7** on the amine at position 2, this group was temporarily masked as an azide, allowing for selective reduction and functionalization post-chain **4** introduction. Selective phosphorylation at the anomeric position (C1)—required for synthesizing **Tetra-C1**—was achieved by 1-O-silyl ether deprotection using fluoride-based reagents. Additionally, positions 4′ and 6′ were protected as a benzylidene acetal, which was removed in the final step of the **Penta-C1** synthesis alongside benzyl groups. Alternatively, regioselective reductive ring opening of the benzylidene acetal revealed a free hydroxy group at C4′, which was subsequently phosphorylated to generate compound **Penta-C4′**.

### 2.3. Synthesis of Disaccharide ***3***

The disaccharide backbone **3** was synthesized via glycosylation with thioglucoside donor **1** of acceptor **2**. The preparation of acceptor **2** began from d-glucosamine hydrochloride, which was transformed into the azido derivative through a diazotransfer reaction using the safe and stable reagent imidazole-1-sulfonyl azide hydrogen sulfate [32], with subsequent O-acetylation to afford intermediate **9**. Selective removal of the acetyl group at the anomeric position, followed by 1-O-silylation, yielded compound **11**. This intermediate was then deacetylated, regioselectively protected at positions 4 and 6 with a benzylidene acetal (giving alcohol **12**), and finally acetylated at 3-OH to generate compound **13**. Reductive opening of the benzylidene acetal using phenylboron dichloride as Lewis acid and triethylsilane as the reducing agent [33] afforded acceptor **2** with an overall yield of 56% across seven synthetic steps.

**Scheme 2:**
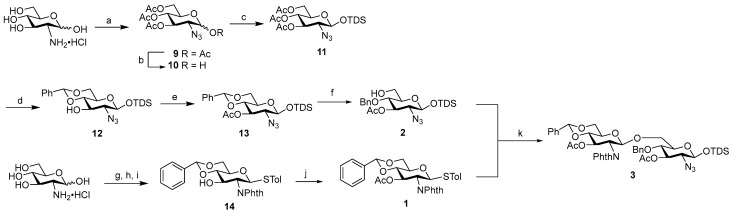
Synthesis of disaccharide **2**. Reagents and conditions. (a) 1. Imidazole-1-sulfonyl azide hydrogen sulfate, K_2_CO_3_, MeOH, 0 °C to r.t., 4 h; 2. Ac_2_O, DMAP, Py, CH_2_Cl_2_, 0 °C to r.t., 12 h 84% over two steps; (b) hydrazine hydrate, AcOH, DMF, 0 °C to r.t., 4 h; (c) TDSCl, imidazole, CH_2_Cl_2_, 0 °C to r.t., 12 h, 92% over two steps; (d) 1. NaOMe, MeOH, r.t., 1 h; 2. PhCH (OMe)_2_, *p*TSA, CH_3_CN, r.t., 8 h; (e) Ac_2_O, DMAP, Py, CH_2_Cl_2_, 0 °C to r.t., 4 h, 80% over three steps; (f) PhBCl_2_, Et_3_SiH, CH_2_Cl_2_, −78 °C, 3 h, 93%, (g) 1. Phthalic anhydride, MeONa, MeOH, Et_3_N, r.t., 24 h, 76% [34]; 2. Ac_2_O, Py, 0 °C to r.t., 48 h, 90% [34]; (h) p-thiocresol, BF_3_ OEt_2_, CH_2_Cl_2_, 0 °C to r.t., 24 h, 91% [34]; (i) 1. NaOMe, MeOH, r.t., 1 h, quant. [34]; 2. PhCH (OMe)_2_, *p*TSA, THF, reflux, 10 h, 84% [34]; (j) Ac_2_O, DMAP, Py, CH_2_Cl_2_, 0 °C to r.t., 2 h, 95%; (k) NIS, TMSOTf, 4 Å MS, dry CH_2_Cl_2_, −20 °C, 2 h, 71%.

Donor **1** was prepared according to the route reported by Liu and Wei in 2012 [34], employing phthalimide as the amino-protecting group. This choice enabled its removal under basic conditions using hydrazine hydrate, which also allowed simultaneous deprotection of the acetyl groups at positions C3′ and C3 of the disaccharide—thereby unveiling the attachment sites for the C16 fatty acid chain derivative **4**. Glucosamine hydrochloride was first reacted with phthalic anhydride to afford the corresponding imide, which was then fully O-acetylated. Thioglycoside formation, subsequent O-deacetylation, and benzylidene protection at 4-OH and 6-OH furnished compound **14** [34]. Final acetylation at 3-OH yielded donor **1**, with an overall yield of 52% (Scheme 2).

The final disaccharide backbone was synthesized by glycosylation of acceptor **2** with donor **1**, promoted by TMSOTf/NIS at –20 °C, affording intermediate **3** in 71% yield (Scheme 2). ^1^H-NMR spectroscopy confirmed the β-configuration of the newly formed glycosidic linkage, as evidenced by the measured J_1-2_ coupling constant of 8.4 Hz for H1′, typical of 1,2-trans glycosides.

### 2.4. Synthesis of the Fatty Acid Chain Derivatives

The synthesis of fatty acid chain derivatives **4**, **6**, and **7** followed a common route starting from two commercially available fatty acids—tetradecanoic and pentadecanoic acid. These precursors were elongated into the corresponding β-ketoesters using a method reported by Brinkerhoff et al. in 2014 [35]. The approach involved initial condensation with Meldrum’s acid and DCC to form enol derivatives, which were subsequently converted into the desired β-ketoesters via acid-mediated reaction with the appropriate alcohol. Using this strategy, we obtained three β-ketoesters (**15**, **16**, and **17**) with yields ranging from 80% to 90%. Compound **17**, which incorporated a tert-butyl group as the alcohol component, proved to be particularly advantageous for the synthesis of the double-chain fatty acid derivative **7**. The tert-butyl group could be selectively removed under mild acidic conditions, thereby preserving the ester functionality at the 3-OH position.

**Scheme 3:**
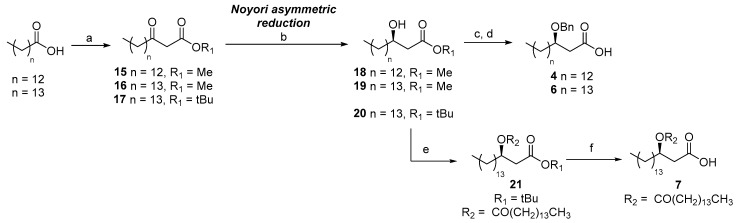
Synthesis of the fatty acid chain derivatives. Reagents and conditions. (a) i. DCC, CH_2_Cl_2_, DMAP, r.t., 45 min, then Meldrum’s acid, Pyridine, CH_2_Cl_2_, r.t., 2 h; ii. R_1_OH, H_2_SO_4_ (cat.), reflux, 16 h, 64–74% yields over three steps; (b) [(R)-BINAP]RuBr_2_ (10 mol%), dry MeOH, H_2_ (balloon), 50 °C, 18 h, **18** (89%), **19** (85%), **20** (73%); (c) PhCHO, TMS_2_O, TMSOTf, THF, 0 °C, 3 h, then Et_3_SiH, 0 °C, 2 h; (d) LiOH, THF/MeOH/H_2_O (3/2/1), rt., 5 h, **4** (80% over two steps), **6** (82% over two steps); (e) pentadecanoic acid, DIC, DMAP, CH_2_Cl_2_, rt, 6 h, quant.; (f) TFA/CH_2_Cl_2_, r.t., 12 h, 93%.

Subsequent efforts focused on the stereoselective reduction of the ketone functionality to achieve the desired (*R*)-configuration at the β-carbon. The application of Noyori reduction conditions, employing the freshly prepared dibromodiphosphineruthenium(II) complex [(R)-BINAP]RuBr_2_ [36], proved highly efficient—yielding (R)-β-hydroxy fatty acids **18**–**20** in good overall yields. The absolute configuration of the resulting β-hydroxy esters was confirmed via Mosher’s ester analysis. This involved performing ^1^H-NMR experiments on the corresponding (*S*)- and (*R*)-MTPA esters [37] (see Supporting Information), as well as complementary NMR studies using the chiral lanthanide shift reagent Eu(hfc)_3_ [38] (see Supporting Information). To prevent racemization, protection of the 3-hydroxy group in compounds **18** and **19** was carried out under carefully selected conditions. Specifically, acid-catalyzed reductive benzylation of compounds **18** and **19** utilizing benzaldehyde [39] and subsequent hydrolysis of the ester groups furnished the final benzyl-protected β-hydroxy fatty acids **4** and **6**. For the synthesis of the double-chain fatty acid derivative **7**, initial condensation with pentadecanoic acid provided compound **21** in quantitative yield. The final fatty acid derivative **7** was obtained by acidic hydrolysis of the tert-butyl ester moiety (Scheme 3).

### 2.5. Fatty Acid Chains Condensation and Final Deprotections

**Scheme 4:**
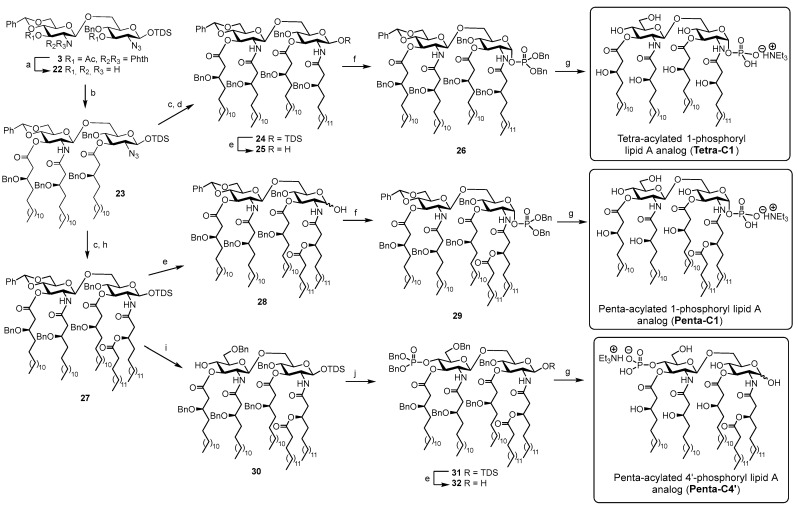
Condensation of fatty acid chains and final deprotection. Reagents and conditions. (a) Hydrazine hydrate, EtOH, 70 °C, 2 h; (b) **4**, CH_2_Cl_2_/DMF (5:1), 45 °C, 12 h, Table 1; (c) Zn, 1,4-dioxane/AcOH (10:1), r.t., 12 h; (d) **6**, HATU, DIPEA, CH_2_Cl_2_/DMF (5:1), rt, 7 h, 87% over two steps; (e) HF·pyridine, THF/Pyridine, r.t., 8 h, 84% (for **25**), 75% (for **28**), 73% (for **32**); (f) tetrabenzyl pyrophosphate, LiHDMS, THF, −70 °C to 0 °C, 1 h, 63% (for **26**), 65% (for **29**); (g) Pd(OH)_2_/C, H_2_ (balloon), CH_2_Cl_2_/MeOH/AcOH (10/10/1), 2 d, then Et_3_N, quant. (for **Tetra-C1**, **Penta-C1** and **Penta-C4′**); (h) **7**, HATU, DIPEA, CH_2_Cl_2_/DMF (5:1), r.t., 4 h, 85% over two steps; (i) Et_3_SiH, TfOH, MS4Å, CH_2_Cl_2_, −78 °C, 3 h, 70%; (j) dibenzyl *N*,*N*-diisopropyl phosphoramidite, 1*H*-tetrazole, CH_2_Cl_2_/CH_3_CN (5:1), then *m*CPBA, rt, 2.5 h, 87%.

The final phase of the synthetic work comprised the following key steps (Scheme 4): (1) deprotection of the phthalimido group together with the cleavage of acetyl esters at the C3 and C3′ hydroxyls; (2) condensation of fatty acid derivative **4** on the newly liberated hydroxyl and amino sites; (3) reduction of the azido group, followed by condensation with fatty acid derivatives **6** or **7**; (4) phosphorylation at either C1 or C4′, followed by final global deprotection. The phthalimide and acetyl groups were removed using hydrazine hydrate in ethanol at 70 °C, and the resulting crude disaccharide **22** was directly used in the subsequent fatty acid coupling step. This stage proved particularly challenging, as the introduction of multiple long-chain and bulky fatty acids required extensive optimization to identify ideal conditions for satisfactory yields. After screening different conditions (Table 1), optimal coupling was achieved using four equivalents of fatty acid **4** and 10 equivalents of EDC hydrochloride. Reduction of the azide at C2 using zinc in acetic acid, followed by condensation with benzyl-protected 3-hydroxy fatty acids **6** and **7**, furnished intermediates **24** and **27** in excellent yields (87% and 85% over two steps, respectively).

Removal of the TDS group from compounds **24** and **27** by treatment with HF·pyridine afforded hemiacetals **25** and **28**, which were phosphorylated using tetrabenzyl pyrophosphate and LiHDMS, yielding protected precursors **26** and **29**, respectively. Final compounds **Tetra-C1** and **Penta-C1** were obtained through hydrogenolysis to remove benzylidene acetal and benzyl groups. For the synthesis of **Penta-C4′**, in which the phosphate group is located at the C4′ hydroxyl, regioselective ring opening of the benzylidene acetal on intermediate **27** generated derivative **30**, delivering the C4′ hydroxyl. The phosphorylation conditions used for compounds **26** and **29** proved to be ineffective in this case. Then, dibenzyl *N*,*N*-diisopropyl phosphoramidite was employed as an alternative phosphorylating agent, affording the C4′-dibenzylphosphate intermediate **31** in excellent yield. Cleavage of the silyl ether at C1 using previously described conditions, followed by exhaustive protecting group cleavage, furnished the final **Penta-C4′** compound.

### 2.6. Docking of Compounds ***1***–***4*** in hTLR4/MD-2 Complex

**Figure 3:**
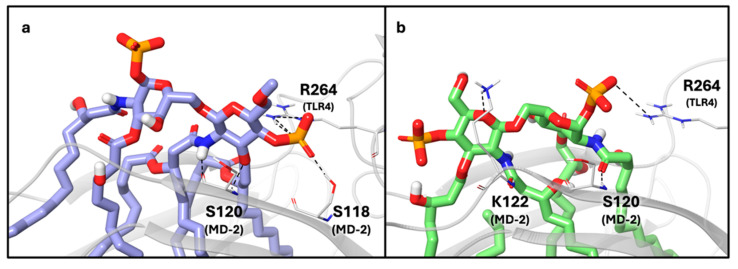
(**a**) X-ray structure (PDB ID: 3FXI) of the lipid A (purple carbon, blue nitrogen, red oxygen, orange sulfur and white polar hydrogen atoms) and (**b**) generated structure (PDB ID: 2E59) of the lipid IVa (green carbon, blue nitrogen, red oxygen, orange sulfur and white polar hydrogen atoms) bound to MD-2 and TLR-4 (grey ribbon representation). Key residues involved in the interactions with the disaccharide unit are shown in tube representation (grey for carbon, blue for nitrogen, red for oxygen and white for polar hydrogen atoms). For lipid A, we highlighted the salt bridge and the hydrogen bond between the ligand phosphate group and R264 (TLR4) and the side chain of S118 (MD2), respectively. For lipid IVa, we highlighted the salt bridge between the ligand phosphate group and R264 (TLR4), the hydrogen bond between the ligand amide and the backbone of S120 (MD-2), and between the sugar ring oxygen and the side chain of K122 (MD-2).

To investigate the binding features of the four compounds of Figure 2, we performed docking calculations in the agonist and antagonist models of TLR4/MD-2 dimer, generated starting from the corresponding X-ray structures (see Section 3 for details). The protein, the sugar core, and the first two torsions of each acyl chain were kept rigid during the docking calculations using AutoDock Vina [40,41]. For each ligand, we run docking simulations in triplicate and build two ligand conformations, one based on the crystallographic di-glucosamine backbone of lipid A bound to MD-2 (agonist-like structure, PDB ID: 3FXI [10]) and the other from lipid IVa (antagonist-like structure, PDB ID: 2E59 [30]) complex. *E. coli* lipid A is the active portion of LPS and acts as an agonist; lipid IVa is a tetra-acylated precursor of lipid A that binds to the same hMD-2/TLR4 complex without triggering the biological response. In the X-ray structures, lipid A and lipid IVa share a similar disaccharide conformation but exhibit an inverted orientation of the entire ligand within the MD-2 pocket, corresponding to a 180° horizontal rotation (Figure S1). In the agonist complex, the sugar moiety of lipid A interacts with residues S118 and S120 at the MD-2 surface, whereas in the antagonist structure, contacts are established with S120 and K122 residues. In both complexes, one phosphate group establishes an electrostatic contact with R264 on TLR4 (Figure 3).

**Figure 4:**
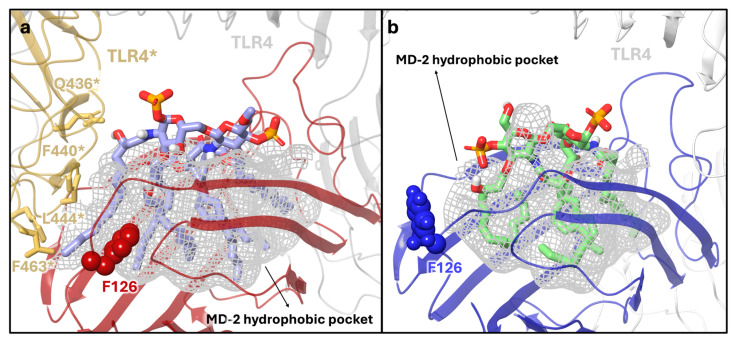
(**a**) X-ray structure (PDB ID: 3FXI) of the lipid A (purple carbon, blue nitrogen, red oxygen, orange sulfur and white polar hydrogen atoms) in complex with MD-2 (red) and two TLR4 proteins (grey and yellow). The MD-2 hydrophobic pocket is represented as a grey meshed surface. MD-2 is shown in red, with residue F126 highlighted in CPK representation in its “closed” conformation, forming hydrophobic interactions with one of the acyl chains of lipid A. One TLR4 chain is shown in grey, the second TLR4* in yellow. The key residues of the TLR4* (yellow)–MD-2 interface are also shown. (**b**) Generated structure of the lipid IV*a* (green carbon, blue nitrogen, red oxygen, orange sulfur and white polar hydrogen atoms) in complex with MD-2 and TLR4 dimer. MD-2 is shown in blue, and F126 is displayed in CPK representation in its “open” conformation, not interacting with the acyl chains of lipid IV*a*. TLR4 is shown in grey.

Beyond the sugar region, more pronounced structural differences are observed in the orientation of the acyl chains. In the agonist state, five of the six lipid A acyl chains bind within the hydrophobic cavity of MD-2, while the sixth chain extends outward from the pocket to interact with a hydrophobic surface on a second TLR4* unit (Figure 4a—the asterisk indicates the TLR4 of the other heterodimer involved in tetramer formation).

This interface, mainly formed by TLR4* residues (Q436, F440, L444, and F463, Figure 4a) and the F126 of the MD-2 loop, has a pivotal role in the recruitment of the second TLR4*∙MD-2*-LPS complex and in the formation of the signaling-competent multimer. Residue F126 is located at the entrance of the MD-2 binding pocket and, after the binding of the ligand, reorients its side chain and creates, together with one LPS chain, the hydrophobic pocket for the interaction with the second TLR4 protein. Site-directed mutagenesis studies confirmed that F126 is essential for receptor dimerization [28].

By contrast, in the antagonist structure, the human lipid IVa is fully embedded within the MD-2 pocket, with all four acyl chains buried inside the pockets and no hydrophobic chain available for interaction with another MD-2/TLR4 unit (Figure 4b). F126 adopts an open conformation (Figure S2) that prevents its side chain from engaging the ligand lipid chain (no contacts between the ligand and F126 are formed).

To evaluate whether our synthesized ligands behave as agonist or antagonist ligands, docking poses were compared to the crystallographic structures of lipid A, for the results in the agonist model, and of lipid IVa, for the antagonist model.

For each ligand, after clustering the docking results based on volume overlap, we first quantified the displacement of the disaccharide moiety from the X-ray structure by calculating the heavy-atom RMSD for each representative binding mode. Low RMSD scores (<2 Å, see Table S1 for the atom selection) indicate a good superimposition of the ligand pose to the experimental structure. In addition, we compared the interactions formed with MD-2 and TLR4 proteins (shown in Figure 3) and the overlay of the acyl chains in the MD-2 pockets to lipid A (agonist docking results) or to lipid IVa (antagonist docking results) by visual inspection. We also considered contacts between the outer lipophilic chain and Phe126 side chain (distance cutoff < 5.5 Å) as indicative of agonist-like behavior. According to these criteria, in the agonist MD-2/TLR4 model, none of the compound geometries reproduces the binding mode of lipid A (Figures S4–S7). With the exception of Penta-C1*, the disaccharide core in all ligands deviates significantly from the crystallographic reference, showing RMSD values greater than 5.84 Å. Even if Penta-C1* in the top-ranked pose is well-aligned to the sugar moiety of lipid A (RMSD = 1.22 Å), none of the X-ray protein–ligand interactions are formed, and the acyl chains are entirely buried within the pocket, and no contacts with Phe126 side chain are present, failing to create the hydrophobic interface required for dimerization (Figure S7a). Taken together, these in silico analyses suggest that none of the compounds can bind to MD-2 like the agonist lipid A.

**Figure 5:**
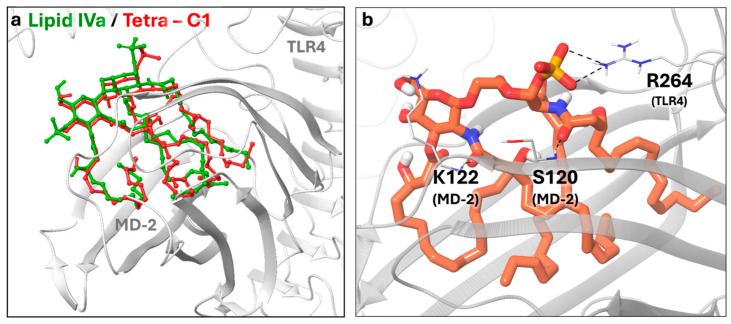
(**a**) Top-ranked pose of the most populated cluster of **Tetra-C1** ligand (red) in the antagonist models overlaid to the crystallographic ligand (green). Both TLR4 and MD-2 are shown in grey. The RMSD range values of the cluster for the di-glucosamine backbone are 0.25–1.05 Å (**b**). Close-up view of the **Tetra-C1** (orange carbon, blue nitrogen, red oxygen, orange sulfur and white polar hydrogen atoms) binding in the MD-2 pocket. Key residues involved in the interactions with the disaccharide unit are shown in tube representation (grey for carbon, blue for nitrogen, red for oxygen and white for polar hydrogen atoms).The salt bridge between the ligand phosphate group and R264 of TLR4, the hydrogen bond between the ligand amide and the backbone of S120 of MD-2, and between the sugar ring oxygen and the side chain of K122 of MD-2 are shown.

Conversely, the analysis of docking poses generated in the antagonist model, besides showing good convergence among the three independent docking runs, highlighted that only the **Tetra-C1** ligand can bind to the MD-2 pocket like the crystallographic ligand. Compound **Tetra-C1** features an antagonist-like binding mode with low RMSD scores in the top-ranked and most populated cluster (50% populated, RMSD range values 0.25–1.05 Å, Table S5 and Figure S8 and Figure 5a,b). All lipid chains are properly accommodated within the MD-2 pocket, and the salt bridge with R264 (TLR4) and hydrogen bonds with S120 and K122 (MD-2) are formed. For Penta-C1*, only a small subset of docking poses shows an antagonist-like positioning of the sugar moiety (8% populated, RMSD range values 0.53–1.76 Å, Table S6) and the interactions with R264 on TLR4 and K122, and S120 residues on MD-2. One acyl chain of Penta-C1* is partially solvent-exposed and poorly aligned to the lipid IVa reference structure (Figure S9). Docking poses of **Penta-C4′** clustered into two main binding modes: the first (12% populated) shows a good sugar core alignment (RMSD range values 0.29–0.98 Å, while the second (57% populated) has a significantly higher displacement (Table S7). In both binding modes, one lipid chain points outside the MD-2 pocket and does not overlap with the antagonist binding mode (Figure S10). Compound **Penta-C1** (Figure S11) adopts two binding modes, both with the disaccharide alignment deviating from the crystallographic pose of lipid IVa (Table S8) and one acyl chain protruding from the MD-2 pocket.

**Figure 6:**
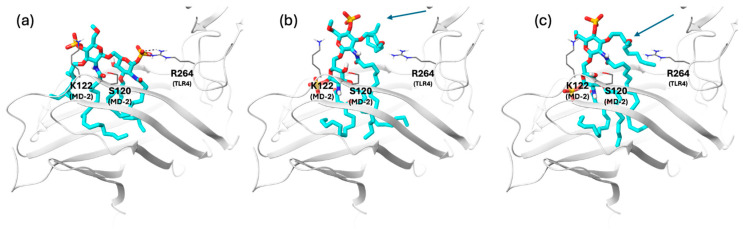
Representative docking poses of Eritoran (orange) in the antagonist MD-2/TLR4 model from cluster analysis. Both TLR4 and MD-2 are shown in grey and Eritoran with cyan carbon, blue nitrogen, red oxygen, orange sulfur and white polar hydrogen atoms. Key residues involved in the interactions with the disaccharide unit are shown in tube representation (grey for carbon, blue for nitrogen, red for oxygen and white for polar hydrogen atoms). (**a**) Cluster 1: moderate disaccharide alignment with the lipid IVa X-ray reference; all acyl chains accommodated in the MD-2 pocket; salt bridge with R264 observed, and the hydrogen bond with K122, consistent with an antagonist-like binding mode. (**b**) Cluster 2: poor disaccharide alignment; one acyl chain protrudes from the pocket as indicated by the arrow. (**c**) Cluster 3: misaligned disaccharide; one acyl chain protrudes toward TLR4 as indicated by the arrow.

To compare our results with a well-characterized TLR4 antagonist, we applied our docking protocol to Eritoran, selecting its crystallographic conformation bound to human MD-2 as the starting point (PDB ID: 2Z65 [28]) and both the antagonist and agonist MD-2/TLR4 models. Despite the structural differences with lipid IVa, the two antagonists bind in a similar way to MD-2, inserting the lipid chains in the hydrophobic pocket. Their di-glucosamine backbones conformation is superimposable (they share a similar conformation) with a 3.25 Å shift (Figure S10) with the MD-2 site. As expected, in the docking results of the agonist model, Eritoran adopted two binding modes, both with poor alignment of the disaccharide core to the lipid A (RMSD range values 7.29–9.31 Å, Table S9, Figure S13). Conversely, in the antagonist model, the top-ranked cluster of docking poses (23% populated), all the acyl chains are properly buried inside the MD-2 hydrophobic pocket, and the interactions with residues K122 (MD-2) and the salt bridge with R264 (TLR4) are formed. The disaccharide core is still not well aligned to lipid IVA, as in the X-ray structures (average RMSD values 4.35 Å, Table S10). The remaining two clusters of docking poses are poorly aligned to both the lipid IVa and Eritoran X-ray structures, with one of the acyl chains partially solvent-exposed and mispositioned outside the MD-2 pocket (Figure 6).

Finally, to provide a consensus ranking that complements the docking scores, docking poses from the antagonist model were re-scored using MM-GBSA calculations [42]. This method estimates the free energies of binding by calculating the difference between the solvation energy of the bound complex and the energy of the unbound protein and ligand using implicit solvent. This method, though it neglects configurational entropy and depends on the quality of force field parameters and solvation model [43], provides a more accurate estimation of binding free energy compared to the docking score, allowing for a better ranking of compounds [44]. Kaus et al. [45] have reported that the MM-GBSA method performs better than standard scoring functions in ranking binding poses. Similarly, Rastelli et al. [46] have shown that both MM-GBSA and MM-PBSA rescoring methodologies improve the affinity ranking estimated by the AutoDock scoring function. Based on this estimation, the complex with the lowest MM-GBSA energy should have the strongest binding affinity. Notably, within the same cluster of docking poses, MM-GBSA values show greater variability compared to docking scores or RMSD values (Tables S5–S8, S10, and S12), reflecting the intrinsic sensitivity of MM-GBSA calculations to small ligand conformational differences. When focusing only on agonist-like binding modes, the reference compounds, Eritoran and lipid IVa, and the **Tertra-C1,** showed comparable affinities based on docking scores, whereas the MM-GBSA values of **Tertra-C1** were higher than those of the reference ligands (Table 2). Experimental validation will be essential to confirm these predictions and to guide further refinement of the model. In addition, advanced MD-based free-energy calculations could be performed in the future to provide more robust and quantitatively reliable estimates of binding free energies.

## 3. Materials and Methods

### 3.1. Docking Studies

#### 3.1.1. Ligands Preparation

The 3D structures of lipid A and lipid IVa were obtained from the corresponding X-ray complexes (PDB ID 3FXI [10] and PDB ID 2E59 [30], respectively). To build the 3D conformations of compounds **1**–**4**, both lipid A and lipid IVa were used as templates, and their hydrophobic side was modified using the “*building panel*“ of the Maestro interface. Only the newly added and modified atoms of the aliphatic chains were locally minimized using the “Minimize Selected Atoms” function of Maestro (OPLS4 force field [47]).

#### 3.1.2. Protein Structures Preparation

For the agonist model, the X-ray structure of 2:2:2 hTLR4, hMD-2, and *E. coli* LPS complex (PDB ID 3FXI [10]) was selected. After removing all the water molecules and ions, the Protein Preparation tool of Maestro (Schrödinger Release 2024-3, version 14.1.138) was used to add hydrogen atoms and determine the protonation states of the protein residues at pH 7 ± 2. The system was relaxed by a restrained energy minimization step with the OPLS3 force field [48] and a heavy atom RMSD convergence set to the default value of 0.3 Å. Chain A of TLR4 and chain C of MD-2 were considered for the final agonist heterodimer model for docking calculations. Since the crystallographic structure of the hTLR4/MD-2 complex in the agonist-bound state is not available, we generated the antagonist model starting from the structure of hMD-2 bound to lipid IVa (PDB ID 2E59, [30]) and adding the TLR4 protein by superimposition to the agonist model, following the procedure described in this work by Lembo-Fazio et al. [49]. We prepared the system for docking calculation by applying the Protein Preparation workflow used for the agonist model. The final structure was then used to generate the grids for docking calculations.

#### 3.1.3. Docking Workflow

Docking calculations were performed with AutoDock Vina [40,41] using a grid spacing of 1 Å and a box size of 60 Å. The grid box (33.00 × 40.50 × 35.25 Å) was centered at the TLR4/MD-2 interface, specifically between the center of mass of R264 (TLR4), R90 (MD-2), and K122 (MD-2) for LPS. For lipid IVa, K122 was replaced by R96 (MD-2), following the protocol reported in the work by Sestito et al. [50]. Gasteiger charges for ligands and receptors were calculated with AutoDockTools 1.5.6 [51], after merging non-polar hydrogens on the receptors. The receptor structures were kept rigid, while ligands were treated as partially flexible. Ligand conformations were generated as described in the ligand preparation section. We considered the saccharide core as rigid, and we introduced dihedral constraints on lipid chains. To determine the number and position of the constraints, the X-ray crystallographic ligands were used as references. For the agonist model, lipid A structure (i.e., the phosphorylated di-glucosamine core and lipid chains) was selected, and for the antagonist model, the lipid IVa. Docking calculations were performed with different combinations of dihedral constraints to identify the setup that best reproduced the experimental binding mode: RMSD of the di-glucosamine core < 2 Å (according to the atoms selection shown in Figure S3); the formation of the relevant polar interactions shown in Figure 3; and all the lipidic chains properly inserted in the MD-2 pocket examined through visual inspection of the docking poses with respect to the X-ray structures and through the formation of ligand–Phe126 contacts (within a 5.5 Å distance cutoff). The best results were obtained by constraining the first two dihedral angles linked to the sugar ring in each hydrophobic chain (dihedral values reported in Table S13). For each ligand, we run three independent docking calculations, and we collected the top-ranked 20 poses, yielding a total of 60 poses (20 poses × 3 runs). These poses were then clustered according to a volume-based overlap criterion (using the utility “phase_volCalc” available in Maestro 2024-3, version 14.1.138) to identify the relevant binding modes of each ligand. We selected all ligand atoms to capture the full spatial shape, enabling a rigorous rigid alignment and grouping based on normalized volume overlap. For the most representative clusters (i.e., those accounting for more than 10% of the total population), docking scores and RMSD values of the saccharide core relative to the reference structures were calculated (Tables S1–S12). The most representative clusters were then evaluated for their capability of reproducing the binding modes of the agonist/antagonist X-ray ligands based on the validation criteria. If all criteria were satisfied, ligand binding modes were classified as agonist-like when the outer acyl chain interacted with the Phe126 side chain (distance < 5.5 Å), and as antagonist-like when no such contact was observed. For each ligand docked in the antagonist model, the most representative clusters were re-scored using the MM-GBSA tool of Prime version 3.0 [42] using the VSGB2.0 implicit water model [52] and the OPLS4 force field [47] after the addition of non-polar hydrogens to both ligands and protein (Tables S5–S8 for compounds **1**–**4**, Table S10 for Eritoran and Table S12 for lipidIVa).

### 3.2. Synthesis—General Remarks

All reagents were purchased from commercial suppliers and were used as supplied without further purification. Dry solvents such as DCM, MeOH, pyridine, toluene, DMF, CH_3_CN, and THF were purchased from Merck© (Darmstadt, Germany) or Thermo Fisher Scientific© (Waltham, MA, USA) and were used without further purification. Non-anhydrous solvents were used directly as supplied from commercial vendors. Analytical thin-layer chromatography (TLC) was performed on Merck© precoated 60F254 plates (0.25 mm), visualized by UV light at 254 nm and/or dipping the plates into stains: molybdic solution (21 g of (NH_4_)_6_Mo_7_O_24_, 1 g of Ce(SO_4_)_2_, 31 mL of H_2_SO_4_ 98%, 970 mL H_2_O) sulfuric acid solution (50 mL of H_2_SO_4_ 98%, 450 mL of MeOH, 450 mL H_2_O), ninhydrin (2.7 g of 2,2-dihydroxyindane-1,3-dione, 27 mL of AcOH, 900 mL of EtOH) with detection by charring at 300 °C. For some reactions, High Performance Thin Layer Chromatography (HPTLC) (Merck© precoated 60 F254 plates 0.20 mm) was used and correspondently pursued. Flash chromatography was performed using Silica gel (SiO_2_, high-purity grade (Merck Grade 9385), pore size 60 Å, 230–400 mesh particle size) from Merck©. Biotage^®^ (Biotage Sweden AB™, Uppsala, Sweden) Isolera SP1 system was used to purify some compounds. Normal phase Biotage cartridges (sizes from 3 g to 340 g, standard 50 μm silica) were employed. Freeze-drying of aqueous solutions was performed using the Lio5P Lypholizer. ^1^H, ^13^C, and ^31^P-NMR spectra were recorded at 25 °C, unless otherwise stated, on a Bruker© Avance TM NEO 400 MHz (Billerica, MA, USA) (400, 100.6 MHz, and 162 MHz for ^1^H, ^13^C, and ^31^P, respectively). All NMR measurements were performed at room temperature. The samples were prepared using deuterated solvents (CDCl_3_, CD_3_COCD_3_, D_2_O, and CD_3_OD from Merck© or Eurisotop^®^). Chemical shifts are reported in ppm and coupling constants (J) in Hz. Multiplicities are abbreviated as br (broad), s (singlet), d (doublet), t (triplet), hept (heptet), m (multiplet), or combinations thereof. ^1^H-NMR spectra were recorded for all the compounds. For unknown structures, characterization is reported by ^1^H-NMR, ^13^C-NMR, and ^31^P-NMR. Low resolution mass analysis was recorded in negative or positive mode on a Thermo© (Waltham, MA, USA) Finnigan LCQAdvantage equipped with an ESI source. High resolution mass spectra (HR-MS) were acquired on a Synapt G2-Si QTof mass spectrometer (Waters™, Milford, MA, USA) equipped with a Zspray ESI-probe (Waters) for electrospray ionization in full scan mode. Data were processed using MassLynx v4.2 software (Waters).

### 3.3. Synthesis—Synthetic Procedures

#### 3.3.1. 1,3,4,6-Tetra-*O*-acetyl-2-deoxy-2-azido-α/β-D-glucopyranose **9**

Glucosamine hydrochloride (7.95 g, 36.9 mmol) was dissolved in MeOH (185 mL). Anhydrous K_2_CO_3_ (15.3 g, 110.7 mmol) was added slowly at 0 °C. After 15 min, imidazole-1-sulfonyl azide hydrogen sulfate (12 g, 44.3 mmol) was added in portions, followed by CuSO_4_∙5H_2_O (92 mg, 0.37 mmol). The reaction progress was monitored through TLC (DCM/MeOH 7:3). After 4 h, the starting material was consumed, and the solvent was removed under reduced pressure and dried under vacuum. The crude 2-azido-2-deoxy-glucopyranose was then suspended in dichloromethane (185 mL) and pyridine (48 mL), then cooled to 0 °C. DMAP (225 mg, 1.85 mmol) and Ac_2_O (27.9 mL, 295 mmol) were added, and the reaction was stirred at r.t. for 12 h. The reaction was followed by TLC (DCM/MeOH 7:3 and hexane/EtOAc 7:3). The reaction was diluted with DCM, then washed three times with 5% HCl solution, twice with saturated solution of NaHCO_3_, and finally with brine. The organic phase was dried with sodium sulfate, filtered, and evaporated. Purification by filtration on silica (hexane/EtOAc 6:4) led to **9** (13 g, 35 mmol) in 95% yield over 2 steps as a 2:1 mixture of the α and β anomers. The spectroscopic data are in agreement with those reported in the literature [53].

#### 3.3.2. 3,4,6-Tri-*O*-acetyl-2-deoxy-2-azido-β-D-glucose **10**

Hydrazine hydrate (1.8 mL, 37.2 mmol) was dissolved in MeOH (30 mL). AcOH (1.77 mL, 31 mmol) was slowly added at 0 °C, and the solution was stirred for 20 min to give hydrazine acetate. The freshly prepared hydrazine acetate solution was slowly added to a solution of **9** (7.7 g, 20.66 mmol) in DMF (40 mL). When the reaction was completed, indicated by TLC (hexane/EtOAc 5:5), the reaction mixture was diluted with Et_2_O and washed 1x H_2_O and 1x brine. The organic phase was dried over Na_2_SO_4_, filtered, and the solvent was removed under reduced pressure, obtaining 8 g of crude compound whose purity was checked by ^1^H-NMR analysis. The spectroscopic data are in agreement with those reported in the literature [54].

#### 3.3.3. 1-*O*-Thexyldimethylsilyl-3,4,6-tri-*O*-acetyl-2-deoxy-2-azido-β-D-glucopyranoside **11**

Crude compound **10** (8 g, 20.66 mmol) was dissolved in DCM (200 mL). Imidazole (3.1 g, 45.45 mmol) was added, and the solution was stirred for 10 min at r.t. The solution was cooled to 0 °C, and the thexyldimethylsilyl chloride (4.9 mL, 24.8 mmol) was added. The reaction was followed by TLC (hexane/EtOAc 7:3). After 12 h, TLC indicated the consumption of the starting material; the reaction mixture was then washed with H_2_O and brine. The organic phase was dried over Na_2_SO_4_, filtered, and the solvent was removed under reduced pressure. The crude was purified by flash chromatography (hexane/EtOAc 7:3), leading to **11** (9.04 g, 19 mmol) with 92% yield over two steps. The spectroscopic data are in agreement with those reported in the literature [53].

#### 3.3.4. 1-*O*-Thexyldimethylsilyl-2-deoxy-2-azido-4,6-*O*-benzylidene-β-D-glucopyranoside **12**

Compound **11** (9.01 g, 19.04 mmol) was dissolved in MeOH (170 mL), then NaOMe (308 mg, 5.71 mmol) was added. After 1 h, as indicated by TLC (hexane/EtOAc 3:7), the reaction was completed. Amberlite IR-120 H+ form was added until pH 6, then the mixture was filtered, and the solution was evaporated to dryness. The residue was dried under vacuum, then redissolved in acetonitrile (175 mL); benzaldehyde dimethylacetal (6.3 mL, 41.9 mmol) and a catalytic amount of *p*TSA (724 mg, 3.8 mmol) were added to the reaction solution. After 8 h, TLC (hexane/EtOAc 3:7) indicated the completion of the reaction. Triethylamine (1.5 mL) was added, and the reaction mixture was evaporated. The purity of the compound, checked by ^1^H-NMR, was found to be adequate for direct use in the subsequent reaction. A sample was purified by flash chromatography hexane/EtOAc 7:3) for characterization.

^1^H-NMR (400 MHz, CDCl_3_) δ 7.34–7.10 (m, 5H, H-Arom), 5.33 (s, 1H, CH benzylidene), 4.43 (d, J = 7.7 Hz, 1H, H-1), 4.09 (dd, J = 10.5, 5.0 Hz, 1H, 6-a), 3.58 (t, J = 10.3 Hz, 1H, 6-b), 3.42 (dd, J = 9.3, 1.5 Hz, 1H, H-3), 3.36 (t, J = 9.1 Hz, 1H, H-4), 3.19 (ddd, J = 10.1, 9.1, 5.0 Hz, 1H, H-5), 3.15–3.04 (m, 1H, H-2), 2.52 (brs, 1H, OH), 1.49 (h, J = 7.1 Hz, 1H, CH-TDS), 0.72 (s, 3H, CH_3_-TDS), 0.70 (s, 9H, CH_3_-TDS), 0.02 (s, 3H, CH_3_Si-TDS), 0.00 ppm (s, 3H, CH_3_Si-TDS).

^13^C-NMR (101 MHz, CDCl_3_) δ 136.87-129.36-128.38-126.29 (C-Arom), 102.00 (CH benzylidene), 97.40 (C-1), 80.76 (C-4), 71.92 (C-3), 69.13 (C-2), 68.58 (C-6), 66.26 (C-5), 33.88 (CH-TDS), 24.81 (Cquat-TDS), 19.98 (CH_3_-TDS), 19.82 (CH_3_-TDS), 18.50 (CH_3_-TDS), 18.40 (CH_3_-TDS), −2.11 (CH_3_Si-TDS), −3.18 ppm (CH_3_Si-TDS).

C_21_H_33_N_3_O_5_Si; calcd. mass 435.22; ESI-MS: *m*/*z* 458.43 [M+Na]^+^.

#### 3.3.5. 1-*O*-Thexyldimethylsilyl-2-deoxy-2-azido-3-*O*-acetyl-4,6-*O*-benzylidene-2-deoxy-β-D-glucopyranoside **13**

Crude compound **12** (10.5 g) was acetylated using a procedure analogous to that reported for compound **9** (3.6 mL of acetic anhydride, 117 mg of DMAP, 6.2 mL of pyridine in 90 mL DCM). The crude compound after extraction was purified by filtration on silica gel (hexane/EtOAc, 7:3), to give compound **13** (7.27 g, 15.2 mmol) with an 80% yield over three steps.

^1^H-NMR (400 MHz, CDCl_3_) δ7.48-7.30 (m, 5H, H-Arom), 5.48 (s, 1H, CH benzylidene), 5.13 (t, J = 9.8 Hz, 1H, H-3), 4.69 (d, J = 7.5 Hz, 1H, H-1), 4.29 (dd, J = 10.3, 5.0 Hz, 1H, H-6a), 3.78 (t, J = 10.3 Hz, 1H, H-6b), 3.63 (t, J = 9.5 Hz, 1H, H-4), 3.48 (dd, J = 5.0, 10.3 Hz, 1H, H-5), 3.43-3.36 (dd, J = 7.5, 9.8 Hz, 1H, H-2), 2.13 (s, 3H, CH_3_CO), 1.67 (hept, J = 6.8, 1H, CH-TDS), 0.96-0.78 (m, 12 H, 4xCH_3_-TDS), 0.22 (s, 3H, *C*H_3_Si-TDS), 0.21 ppm (s, *C*H_3_Si-TDS).

^13^C-NMR (101 MHz, CDCl_3_) δ169.88 (CH_3_*C*O), 136.98-129.25-128.38-126.28 (C-Arom), 101.66 (C-benzylidene), 97.61 (C-1), 78.85 (C-4), 71.29 (C-3), 68.68 (C-6), 67.45 (C-2), 66.66 (C-5), 34.01 (*C*H-TDS), 21.02 (CH_3_CO), 20.07-19.94-18.64-18.53 (CH_3_-TDS), −2.03 (CH_3_Si-TDS), −3.09 ppm (*C*H_3_Si-TDS).

C_23_H_35_N_3_O_6_Si; calcd. mass 477.23; ESI-MS: *m*/*z* 500.31 [M+Na]^+^.

#### 3.3.6. 1-*O*-Thexyldimethylsilyl-2-deoxy-2-azido-3-*O*-acetyl-4-*O*-benzyl-2-deoxy-β-D-glucopyranoside **2**

Compound **13** (2.3 g, 4.85 mmol) was dissolved in DCM (95 mL), then freshly activated 4Å MS were added under an Ar atmosphere. The mixture was stirred for 1 h at −78 °C before the addition of Et_3_SiH (2.7 mL, 17 mmol). The mixture was stirred for an additional 15 min, then PhBCl_2_ (0.69 mL, 5.34 mmol) was added. The reaction progress was monitored by TLC (hexane/EtOAc 8:2). When the reaction was completed (3 h), it was quenched at −55 °C by the addition of Et_3_N (1.5 mL) and MeOH (5 mL). The MS were removed by filtration over celite, the organic phase was washed once with NaHCO3 (saturated aqueous solution), thenH2O, brine, and dried over Na2SO4. After filtration, the crude was concentrated in vacuo, then purified by flash chromatography (hexane/EtOAc 8:2) to give pure **2** (2.1 g, 4.5 mmol) in 93% yield.

^1^H-NMR (400 MHz, CDCl_3_) δ 7.42-7.25 (m, 5H, CH Ar), 5.06 (dd, J = 10.4, 9.3 Hz, 1H, H-3), 4.46 (d, J = 7.7, 1H, H-1), 4.63 (d, J = 3.7 Hz, 2H, CH_2_Ph), 3.88 (ddd, J = 12.2, 5.3, 2.7 Hz, 1H, H-6a), 3.74 (ddd, J = 11.9, 8.3, 3.9, 1H, H-6b), 6.65 (t, J = 9.5 Hz, 1H, H-4), 3.42 (ddd, J = 9.8, 4.1, 2.6 Hz, 1H, H-5), 3.30 (dd, J = 10.4, 7.7 Hz, 1H, H-2), 2.05 (s, 3H, CH_3_CO), 1.80 (dd, J = 8.4, 5,4, 1H, OH), 1.67 (hept, J = 6.8, 1H, CH-TDS), 0.92 (s, 3H, CH_3_-TDS), 0.90 (s, 9H, CH_3_-TDS), 0.22 (s, 3H, *C*H_3_Si-TDS), 0.21 ppm (s, 3H, *C*H_3_Si-TDS).

^13^C-NMR (101 MHz, CDCl_3_) δ169.98 (CH_3_*C*O), 137.65 (CquatAr), 128.70, 128.16, 127.94 (CAr), 97.01 (C-1), 75.72 (C-4), 75.40 (C-5), 74.72 (*C*H_2_Ph), 74.14 (C-3), 67.01 (C-2), 61.89 (C-6), 34.07 (*C*H-TDS), 25.30 (Cquat), 21.07 (*C*H_3_CO), 20.11-20.10-18.57-18.54 (*C*H_3_-TDS), 1.96 (*C*H_3_Si-TDS), -3.00 ppm (*C*H_3_Si-TDS).

C_23_H_37_N_3_O_6_Si; calcd. mass 479.25; ESI-MS: *m*/*z* 502.49 [M+Na]^+^.

#### 3.3.7. 1-*S*-(4-Methylphenyl)-2-deoxy-2-phthalimido-3-*O*-acetyl-4,6-*O*-benzylidene-β-D-glucopyranoside **1**

Compound **14** [34] (3.05 g, 6.06 mmol) was dissolved in DCM (30 mL), then DMAP (37 mg, 0.05 mmol) and pyridine (1.96 mL, 24 mmol) were added; the reaction was cooled to 0 °C then acetic anhydride (1.15 mL, 12.1 mmol) was added slowly, then the reaction was raised to r.t. After 2 h TLC (hexane/EtOAc 75:25) indicated the disappearance of the starting material. The reaction was worked up as for compounds **9** and **13**; the product was purified by flash chromatography, using hexane/EtOAc 75:25 as eluent, affording compound **1** (3.1 g) with a yield of 95%.

^1^H NMR (400 MHz, CDCl_3_) δ 7.91–7.83 (m, 2H, CHAr), 7.80–7.70 (m, 2H, CHAr), 7.47–7.40 (m, 2H, CHAr), 7.40–7.32 (m, 3H, CHAr), 7.32–7.24 (m, 2H, CHAr), 7.08 (d, J = 8.0 Hz, 2H, CHAr), 5.88 (t, J = 9.4 Hz, 1H, H-3), 5.76 (d, J = 10.5 Hz, 1H, H-1), 5.53 (s, 1H, CH benzylidene), 4.46–4.38 (m, 1H, H-5), 4.33 (dd, J = 10.6, 9.9 Hz, 1H, H-2), 3.87–3.69 (m, 3H, H-6, H-4), 2.32 (s, 3H, C*H_3_*Ph), 1.87 (s, 3H, C*H_3_*CO).

^13^C NMR (101 MHz, CDCl_3_) δ 170.32 (CH_3_*C*O), 168.01 (*C*(O)N Phth), 167.41 (*C*(O)N Phth), 138.87 (Cquat Ar), 137.01 (Cquat Ar), 134.58 (CAr), 134.35 (CAr), 133.86 (CAr), 131.86 (Cquat Ar), 131.37 (Cquat Ar), 129.90 (CAr), 129.33 (CAr), 128.40 (CAr), 127.35 (Cquat Ar), 126.40 (CAr), 123.87 (CAr), 123.75 (CAr), 101.80 (*C*HPh), 84.15 (C-1), 79.16 (C-4), 70.79 (C-3), 70.66 (C-5), 68.75 (C-6), 54.50 (C-2), 21.33 (*C*H_3_-STol), 20.71 (*C*H_3_CO).

C_30_H_27_NO_7_S; calcd. mass 545.15; ESI-MS: *m*/*z* 568,32 [M+Na]^+^.

#### 3.3.8. 6-*O*-(2-Deoxy-2-phthalimido-3-*O*-acetyl-4,6-*O*-benzylidene-β-D-glucopyranosyl)-β-1-(*O*-thexyldimethylsilyl)-2-deoxy-2-azido-3-*O*-acetyl-4-*O*-benzyl-β-D-glucopyranoside **3**

Compound **1** (3.195 g, 5.85 mmol) and compound **2** (2.58 g, 5.38 mmol) were co-evaporated three times with toluene and stored under vacuum overnight. Then, activated 4Å MS were added under an Ar atmosphere, 50 mL of dry DCM was added, and the mixture was stirred at r.t. for 1 h. Then the reaction was cooled to −20 °C; NIS (1.6 g, 7 mmol) and after 3 min TMSOTf (0.1 mL, 0.538 mmol) were added. The reaction was stirred at this temperature for 2 h, when TLC (hexane/EtOAc 7:3) showed complete consumption of the acceptor. The reaction was quenched by adding 5 mL of triethylamine at −20 °C, then raised to r.t.; the mixture was filtered over celite, and the filtrate concentrated to dryness. The residue was purified by silica gel chromatography (hexane/EtOAc 8:2), affording disaccharide **3** (3.44 g) in a yield of 71%.

^1^H NMR (400 MHz, CDCl_3_) δ 7.77–7.72 (m, 2H, CHAr), 7.67–7.58 (m, 2H, CHAr), 7.48–7.40 (m, 2H, CHAr), 7.40–7.28 (m, 3H, CHAr), 7.24–7.15 (m, 4H, CHAr), 7.00–6.91 (m, 2H, CHAr), 5.82 (dd, J = 10.3, 9.1 Hz, 1H, H-3′), 5.52 (s, 1H, CHPh), 5.49 (d, J = 8.4 Hz, 1H, H-1′), 4.90 (dd, J = 10.5, 8.6 Hz, 1H, H-3), 4.49 (d, J = 7.6 Hz, 1H, H-1), 4.39–4.31 (m, 2H, H-2′, H-6a’), 4.31–4.21 (m, 2H, CH_2_Ph), 3.98 (dd, J = 10.7, 1.7 Hz, 1H, H-6b), 3.85–3.73 (m, 2H, H-4′, H-6b’), 3.73–3.63 (m, 2H, H-6a, H-5′), 3.47–3.33 (m, 2H, H-4, H-5), 3.18 (dd, J = 10.4, 7.7 Hz, 1H, H-2), 1.95 (s, 3H, CH_3_CO), 1.87 (s, 3H, CH_3_CO), 1.63–1.55 (m, 1H, CH-TDS), 0.83 (dd, J = 6.8, 1.3 Hz, 6H, CH_3_-TDSx2), 0.79 (s, 6H, CH_3_-TDSx2), 0.08 (s, 3H, CH_3_Si-TDS), 0.00 (s, 3H, CH_3_Si-TDS).

^13^C NMR (101 MHz, CDCl_3_) δ 170.35 (CH_3_*C*O), 169.87 (CH_3_*C*O), 137.43 (CquatAr), 137.06 (CquatAr), 134.37 (CquatAr), 131.57 (CquatAr), 129.34 (CAr), 128.50 (CAr), 128.41 (CAr), 128.03 (CAr), 127.75 (CAr), 126.40 (CAr), 123.70 (CAr), 101.86 (*C*HPh), 98.42 (C-1′), 97.01 (C-1), 79.34 (C-4′), 76.30 (C-4), 74.52 (*C*H_2_Ph), 74.27 (C-5), 73.95 (C-3), 70.11 (C-3′), 68.81 (C-6′), 68.13 (C-6), 66.72 (C-2), 66.43 (C-5′), 55.43 (C-2′), 34.01 (*C*H-TDS), 24.89 (Cquat-TDS), 21.00 (*C*H_3_CO), 20.69 (*C*H_3_CO), 19.9954 (*C*H_3_-TDS), 19.9854 (*C*H_3_-TDS), 18.5954 (*C*H_3_-TDS), 18.5554 (*C*H_3_-TDS), −1.97 (*C*H_3_Si-TDS), −3.39 (*C*H_3_Si-TDS).

C_46_H_56_N_4_O_13_Si: calcd. mass 900.36; ESI-MS: *m*/*z* 901.76 [M+H]^+^, 923.64 [M+Na]^+^.

#### 3.3.9. General Procedure for the Synthesis of β-Ketoesters

The appropriate carboxylic acid (10 mmol) was dissolved in DCM (100 mL); DCC (11 mmol) and DMAP (3 mmol) were added at r.t. and the reaction was stirred at this temperature for 1 h. A white precipitate formed during the reaction. Then Meldrum’s acid (20 mmol) was dissolved in a DCM/pyridine solution (100 + 3 mL) and stirred at r.t. for 45 min, during which the solution turned pink. This solution was then slowly added to the first flask, and the reaction was stirred at r.t. After 3 h, TLC (hexane/EtOAc 7:3) showed the disappearance of the starting material; the reaction was then filtered, the filtrate washed with 5% HCl and then brine. The organic phase was dried with sodium sulfate, filtered, and evaporated. The crude residue was dissolved in the appropriate alcohol (100 mL, MeOH for **15** and **16** or tBuOH for **17**), then 5% mol of sulfuric acid was added, and the solution was refluxed for 15 h. The reaction was then evaporated, and the crude purified on silica gel (hexane/EtOAc 9:1 to 8:2).

Methyl 3-oxohexadecanoate **15**. Yield: 74%. The spectroscopic data are in agreement with those reported in the literature [55].

Methyl 3-oxoheptadecanoate **16**. Yield: 64%.

^1^H-NMR (400 MHz, Chloroform-*d*) δ 3.73 (s, 3H, OCH_3_), 3.44 (s, 2H, CO-C*H*_2_-CO_2_Me), 2.52 (t, J = 7.4 Hz, 2H, CO-C*H*_2_-CH_2_-Chain), 1.64–1.53 (m, 2H, CO-CH_2_-C*H*_2_-Chain), 1.34–1.17 (m, 22H, chain), 0.88 (t, J = 6.7 Hz, 3H, CH_3_). ^13^C NMR (101 MHz, CDCl_3_) δ 202.98 (*C*O), 167.83 (*C*O_2_Me), 52.44 (CO_2_*C*H_3_), 49.14, 43.22, 32.06, 29.79, 29.72, 29.57, 29.48, 29.32, 29.14, 23.61, 22.82 (*C*H_2_ chain), 14.24 (*C*H_3_). C_18_H_34_O_3_: calcd. mass 298.25; ESI-MS: *m*/*z* 321.47 [M+Na]^+^.

t-Butyl 3-oxoheptadecanoate **17**. Yield: 73%.

^1^H-NMR (400 MHz, CDCl_3_) δ 3.26 (s, 2H, CO-C*H_2_*-CO_2_tBu), 2.44 (t, J = 7.4 Hz, 2H, C*H_2_*), 1.50 (q, J = 7.0 Hz, 2H, CH*_2_*), 1.41 (d, J = 7.3 Hz, 9H, tBu), 1.19 (d, J = 7.8 Hz, 26H, CH_2_ chain), 0.88–0.73 (m, 3H, CH_3_). ^13^C NMR (101 MHz, CDCl_3_) δ 203.21 (*C*O), 166.48 (*C*O_2_tBu), 50.61, 42.89, 31.94, 29.71, 29.69, 29.67, 29.62, 29.50, 29.47, 29.43, 29.40, 29.37, 29.21, 29.14, 29.09, 27.95 (*C*H_3_ tBu), 23.50, 22.69. C_21_H_40_O_3_: calcd. mass 340.30; ESI-MS: *m*/*z* 363.29 [M+Na]^+^.

#### 3.3.10. General Procedure for the Stereoselective Reduction of β-Ketoesters to (*R*)-β-Hydroxyesters

The stereoselective reductions of β-ketoesters were performed employing a slightly modified procedure reported by Ratovelomanana et al. [36]. Briefly, the β-ketoester (3.5 mmol) was dissolved in degassed MeOH (7 mL, 0.5 M) and added to the freshly prepared [(*R*)-BINAP]RuBr_2_ catalyst (0.07 mmol) [36]. The mixture was placed under argon, and then the atmosphere was replaced with hydrogen (from a balloon). The reaction was heated to 50 °C for 16 h, and the progress of the reaction was checked by TLC (hexane/EtOAc 7:3). The reaction was evaporated, and the crude product was purified by flash chromatography (hexane/EtOAc 8:2), affording the respective (R)-β-hydroxyesters.

Methyl 3-hydroxyhexadecanoate **18**. Yield: 89%. The spectroscopic data are in agreement with those reported in the literature [36]. Mosher’s esters analysis [37] and the ^1^H-NMR experiment employing the chiral lanthanide shift reagent Eu(Hfc)_3_ [38] confirmed the stereoselective reduction, affording the (*R*)-enantiomers (see Supporting Information).

Methyl 3-hydroxyheptadecanoate **19**. Yield: 85%.

^1^H NMR (400 MHz, CDCl_3_) δ 4.07–3.93 (m, 1H, H-3), 3.71 (s, 3H, OC*H*_3_), 2.83 (d, J = 4.0 Hz, 1H, OH), 2.52 (dd, J = 16.4, 3.1 Hz, 1H, H-2a), 2.41 (dd, J = 16.4, 9.0 Hz, 1H, H-2b), 1.48–1.38 (m, 2H, H-4), 1.26 (s, 22H, CH_2_ chain), 0.88 (t, J = 6.7 Hz, 3H, CH_3_).

^13^C NMR (101 MHz, CDCl_3_) δ 173.63 (*C*OOMe), 68.16 (C-3), 51.84 (O*C*H_3_), 41.25 (C-2), 36.68 (C-4), 32.05 (*C*H_2_ chain), 29.80 (*C*H_2_ chain), 29.71 (*C*H_2_ chain), 29.65 (*C*H_2_ chain), 29.49 (*C*H_2_ chain), 29.41 (*C*H_2_ chain), 25.61 (*C*H_2_ chain), 22.82 (*C*H_2_ chain), 14.24 (*C*H_3_). C_18_H_36_O_3_: calcd. mass 300.27; ESI-MS: *m*/*z* 300.99 [M+H]^+^.

t-Butyl-3-hydroxyheptadecanoate **20**. Yield: 73%.

^1^H NMR (400 MHz, CDCl_3_) δ 3.98–3.88 (m, 1H, H-3), 3.09 (s, 1H, OH), 2.41 (dd, J = 16.3, 3.2 Hz, 1H, H-2a), 2.30 (dd, J = 16.3, 8.9 Hz, 1H, H-2b), 1.45 (s, 9H, CH_3_ tBu), 1.43–1.34 (m, 2H, H-4), 1.33–1.18 (m, 24H, CH_2_ chain), 0.91–0.83 (m, 3H, CH_3_). ^13^C NMR (101 MHz, CDCl_3_) δ 172.71 (*C*OOtBu), 81.30 (Cquat tBu), 68.04 (C-3), 42.42 (C-2), 36.62 (C-4), 32.07 (*C*H_2_ chain), 29.83 (*C*H_2_ chain), 29.80 (*C*H_2_ chain), 29.78 (*C*H_2_ chain), 29.72 (*C*H_2_ chain), 29.70 (*C*H_2_ chain), 29.49 (*C*H_2_ chain), 28.24 (*C*H_3_ tBu), 25.60 (*C*H_2_ chain), 22.80 (*C*H_2_ chain), 14.23 (*C*H_3_). C_21_H_42_O_3_: calcd. mass 342.31; ESI-MS: *m*/*z* 343.65 [M+H]^+^.

#### 3.3.11. General Procedure for the Reductive Benzylation of the 3-Hydroxyl Group of Fatty Acids and Base-Mediated Ester Hydrolysis

Respective Methyl 3-hydroxyhexadecanoate (**18**) or heptadecanoate (**19**) (0.67 mmol) was dissolved in dry THF (1.3 mL), and the solution was cooled to 0 °C. After the addition of benzaldehyde (2.0 mmol) and hexamethyldisiloxane (4.0 mmol), trimethylsilyl trifluoromethanesulfonate (1.3 mmol) was added dropwise, and the reaction mixture was stirred for 3 h at 0 °C. Then, triethylsilane (2.0 mmol) was added, and after stirring for 2 h at 0 °C, the reaction mixture was diluted with EtOAc, washed with saturated aqueous NaHCO_3_ solution and brine; dried over Na_2_SO_4_, filtered, and concentrated.

To the crude was added THF (3 mL), MeOH (2 mL), and H_2_O (1 mL). To the solution was added LiOH.H_2_O (2.7 mmol), and the reaction was stirred at room temperature for 5 h. The reaction mixture was then diluted with EtOAc, washed with aqueous 1 N HCl, deionized H_2_O, and brine; dried over Na_2_SO_4_, filtered, and concentrated. The crude was purified by flash silica gel column chromatography (hexane/EtOAc 7:3), affording the respective (*R*)-3-(benzyloxy) fatty acids.

(*R*)-3-(benzyloxy)-hexadecanoic acid **4**; Yield: 80%. The spectroscopic data are in agreement with those reported in the literature [27].

(*R*)-3-(benzyloxy)-heptadecanoic acid **6**; Yield: 82%.

^1^H NMR (400 MHz, CDCl_3_) δ 7.28–7.17 (m, 5H, CHAr), 4.56–4.45 (m, 2H, C*H*_2_Ph), 3.81 (ddd, J = 12.2, 6.8, 5.5 Hz, 1H, H-3), 2.58 (dd, J = 15.4, 7.1 Hz, 1H, H-2a), 2.48 (dd, J = 15.4, 5.2 Hz, 1H, H-2b), 1.66–1.43 (m, 2H, H-4), 1.40–1.14 (m, 24H, CH_2_ chain), 0.82 (t, J = 6.6 Hz, 3H, CH_3_). ^13^C NMR (101 MHz, CDCl_3_) δ 177.47 (*C*OOMe), 138.29 (Cquat Ar), 128.52 (CAr), 127.98 (CAr), 127.84 (CAr), 75.87 (C-3), 71.69 (*C*H_2_Ph), 39.69 (C-2), 34.29 (C-4), 32.07 (*C*H_2_ chain), 29.83 (*C*H_2_ chain), 29.82 (*C*H_2_ chain), 29.80 (*C*H_2_ chain), 29.79 (*C*H_2_ chain), 29.73 (*C*H_2_ chain), 29.70 (*C*H_2_ chain), 29.51 (*C*H_2_ chain), 25.26 (*C*H_2_ chain), 22.84 (*C*H_2_ chain), 14.26 (*C*H_3_). C_24_H_40_O_3_: calcd. mass 376.30; ESI-MS: *m*/*z* 399.70 [M+Na]^+^.

#### 3.3.12. t-Butyl (R)-3-(Pentadecanoyloxy)-Heptadecanoate **21**

To the solution of *t*-Butyl (*R*)-3-hydroxy-heptadecanoate **20** (1.04 g, 2.92 mmol) and pentadecanoic acid (0.92 g, 3.79 mmol) in dry CH_2_Cl_2_ (29 mL) was added DIC (904 μL, 5.84 mmol) and DMAP (0.36 g, 2.92 mmol). The reaction was stirred at room temperature for 6 h, and then it was diluted with CH_2_Cl_2_. The diisopropylurea (DIU) precipitate was filtered off, and the filtrate was washed with saturated aqueous NH_4_Cl solution and brine, dried over Na_2_SO_4_, filtered, and concentrated. The crude was purified by flash silica gel column chromatography (hexane/EtOAc = 19:1) to give **21** (1.7 g, quant.)

^1^H NMR (400 MHz, CDCl_3_) δ 5.19 (tt, *J* = 7.3, 5.7 Hz, 1H, H-3), 2.53–2.37 (m, 2H, H-2), 2.29–2.21 (m, 2H, H-2′), 1.58 (qd, J = 11.5, 6.1 Hz, 4H, H-4, H-3′), 1.42 (s, 9H, CH_3_ tBu), 1.25 (d, J = 5.1 Hz, 46H, C*H*_2_ chains), 0.91–0.82 (m, 6H, CH_3_ × 2).

^13^C NMR (101 MHz, CDCl_3_) δ 173.19 (*C*OOtBu), 169.84 (*C*OO C15 chain), 80.82 (Cquat tBu), 70.64 (C-3), 40.75 (C-2), 34.65 (C-2′), 34.19 (C-4), 32.05 (*C*H_2_ chains), 29.84 (*C*H_2_ chains), 29.82 (*C*H_2_ chains), 29.81 (*C*H_2_ chains), 29.79 (*C*H_2_ chains), 29.78 (*C*H_2_ chains), 29.75 (*C*H_2_ chains), 29.68 (*C*H_2_ chains), 29.63 (*C*H_2_ chains), 29.62 (*C*H_2_ chains), 29.53 (*C*H_2_ chains), 29.50 (*C*H_2_ chains), 29.43 (*C*H_2_ chains), 29.31 (*C*H_2_ chains), 28.15 (*C*H_3_ tBu), 25.25 (*C*H_2_ chains), 25.17 (*C*H_2_ chains), 22.83 (*C*H_2_ chains), 14.24 (*C*H_3_). C_36_H_70_O_4_: calcd. mass 566.53; ESI-MS: *m*/*z* 589.63 [M+Na]^+^.

#### 3.3.13. (*R*)-3-(Pentadecanoyloxy)-Heptadecanoic Acid **7**

Compound **21** (1.6 g, 2.82 mmol) was dissolved in CH_2_Cl_2_ (23 mL), and then TFA (6 mL) was added, and the reaction mixture was stirred at room temperature for 12 h. The reaction was diluted with CH_2_Cl_2_, washed with saturated aqueous NaHCO_3_ solution and brine; dried over Na_2_SO_4_, filtered, and concentrated. The crude was purified by flash silica gel column chromatography (hexane/EtOAc = 6:4) to give **7** (1.3 g, 93%).

^1^H NMR (400 MHz, CDCl_3_) δ 5.20 (t, J = 6.5 Hz, 1H, H-3), 2.67–2.51 (m, 2H, H-2), 2.27 (t, J = 7.5 Hz, 2H, H-2′), 1.60 (h, J = 7.2 Hz, 4H, H-4, H-3′), 1.46–1.04 (m, 46H, CH_2_ chains), 0.94–0.80 (m, 6H, CH_3_ x 2). ^13^C NMR (101 MHz, CDCl_3_) δ 176.69 (*C*OOH), 173.44 (*C*OO C15 chain), 70.14 (C-3), 39.06 (C-2), 34.62 (C-2′), 34.11 (C-4), 32.07 (*C*H_2_ chains), 29.86 (*C*H_2_ chains), 29.85 (*C*H_2_ chains), 29.82 (*C*H_2_ chains), 29.81 (*C*H_2_ chains), 29.78 (*C*H_2_ chains), 29.70 (*C*H_2_ chains), 29.64 (*C*H_2_ chains), 29.51 (*C*H_2_ chains), 29.49 (*C*H_2_ chains), 29.43 (*C*H_2_ chains), 29.26 (*C*H_2_ chains), 25.25 (*C*H_2_ chains), 25.15 (*C*H_2_ chains), 22.85 (*C*H_2_ chains), 14.25 (*C*H_3_). C_32_H_62_O_4_: calcd. mass 510.46; ESI-MS: *m*/*z* 533.46 [M+Na]^+^.

#### 3.3.14. 6-*O*-(2-Deoxy-2-((*R*)-3-(benzyloxy)-hexadecanoylamido)-3-*O*-((*R*)-3-(benzyloxy)-hexadecanoyl)-4,6-*O*-benzylidene-β-D-glucopyranosyl)-1-(*O*-thexyldimethylsilyl)-2-deoxy-2-azido-3-*O*-((*R*)-3-(benzyloxy)-hexadecanoyl)-4-*O*-benzyl-β-D-glucopyranoside **23**

Disaccharide **3** (620 mg, 0.688 mmol) was dissolved in absolute ethanol (20 mL) with sonication to obtain a cloudy solution. Then, hydrazine monohydrate (2 mL) was added, and the reaction mixture was stirred at 70 °C for 2 h. After cooling to room temperature, the reaction was diluted with CH_2_Cl_2_, washed with deionized H_2_O and brine; dried over Na_2_SO_4_, filtered, and concentrated.

A mixture of the obtained crude compound **22** and benzyl-protected fatty acid **4** (1.0 g, 2.75 mmol) was co-evaporated with toluene twice and dried in vacuo for 30 min. To the mixture was added dry CH_2_Cl_2_ (5.8 mL) and dry DMF (1.2 mL), and stirred at room temperature. Then EDC.HCl (1.3 g, 6.88 mmol) and DMAP (126 mg, 1.03 mmol) were added, and the reaction was stirred at 45 °C for 12 h. After dilution with CH_2_Cl_2_, the reaction mixture was washed with saturated aqueous NH_4_Cl solution and brine, dried over Na_2_SO_4_, filtered, and concentrated. The crude was purified by flash silica gel column chromatography (Toluene/EtOAc = 19:1) to give **23** (0.9 g, 75%).

^1^H NMR (400 MHz, CDCl_3_) δ 7.52–7.18 (m, 25H, CHAr), 6.32 (d, J = 9.0 Hz, 1H, N*H*), 5.42 (s, 1H, H-7′), 5.31 (t, J = 9.8 Hz, 1H, H-3′), 5.03 (dd, J = 10.4, 9.1 Hz, 1H, H-3), 4.62–4.35 (m, 11H), 4.27 (dd, J = 10.4, 4.9 Hz, 1H), 4.03–3.61 (m, 10H), 3.56 (t, J = 9.4 Hz, 1H), 3.41 (dtd, J = 11.7, 5.8, 3.9 Hz, 2H), 3.26 (dd, J = 10.4, 7.6 Hz, 1H, H-2), 2.73–2.53 (m, 2H), 2.51–2.40 (m, 2H), 2.34–2.22 (m, 2H), 1.73–1.66 (m, 1H, CH TDS), 1.62–1.38 (m, 8H), 1.38–1.10 (m, 70H, CH_2_ chains), 0.96–0.87 (m, 21H, CH_3_ (chains + TDS)), 0.22 (d, J = 3.1 Hz, 6H, CH_3_ TDS). ^13^C NMR (101 MHz, CDCl_3_) δ 171.38, 171.30, 170.80, 138.75, 138.70, 138.46, 137.92, 137.03, 129.15, 128.73, 128.56, 128.54, 128.39, 128.37, 128.29, 128.27, 127.96, 127.89, 127.88, 127.82, 127.57, 127.55, 126.25, 101.61, 101.56, 97.06, 79.04, 76.26, 76.14, 75.88, 75.69, 74.62, 74.30, 74.11, 71.76, 71.63, 71.27, 70.95, 68.78, 67.93, 66.90, 66.53, 54.62, 41.36, 39.88, 34.66, 34.51, 34.07, 33.84, 32.07, 29.85, 29.83, 29.81, 29.79, 29.77, 29.74, 29.73, 29.50, 25.28, 25.26, 25.23, 25.21, 24.98, 22.82, 20.13, 20.08, 18.65, 18.60, 14.23, −1.63, −3.17.

C_103_H_158_N_4_O_15_Si: calcd. mass 1719.15; ESI-MS: *m*/*z* 1742.42 [M+Na]^+^.

#### 3.3.15. General Procedure for Azide Reduction

To a solution of compound **23** (27 mg, 0.016 mmol) in 1,4-dioxane (1 mL) and AcOH (0.1 mL) was added Zn powder (150 mg), and after stirring the reaction for 12 h at room temperature, it was diluted with CH_2_Cl_2_, filtered over celite, washed with saturated aqueous NaHCO_3_ solution and brine; dried over Na_2_SO_4_, filtered and concentrated to obtain the amine as crude which was used in the next reaction without further purification.

#### 3.3.16. 6-*O*-(2-Deoxy-2-((*R*)-3-(benzyloxy)-hexadecanoylamido)-3-*O*-((*R*)-3-(benzyloxy)-hexadecanoyl)-4,6-*O*-benzylidene-β-D-glucopyranosyl)-1-(*O*-thexyldimethylsilyl)-2-deoxy-2-((*R*)-3-(benzyloxy)-heptadecanoylamido)-3-*O*-((*R*)-3-(benzyloxy)-hexadecanoyl)-4-*O*-benzyl-β-D-glucopyranoside **24**

The crude amine obtained after azide reduction (0.019 mmol) was co-evaporated with toluene and dried in vacuo. To the crude was added benzyl-protected fatty acid **6** (11 mg, 0.029 mmol) and HATU (15 mg, 0.038 mmol), and the mixture was dissolved in dry CH_2_Cl_2_ (1 mL) and DMF (0.2 mL). After adding DIPEA (7 µL), the reaction was stirred at room temperature for 7 h. The reaction mixture was then diluted with CH_2_Cl_2_, washed with saturated aqueous NH_4_Cl solution and brine, dried over Na_2_SO_4_, filtered, and concentrated. The crude was purified by size exclusion chromatography (Sephadex^®^ LH-20) (CHCl_3_/MeOH = 1:1) to give **24** (34 mg, 87%).

^1^H NMR (400 MHz, CDCl_3_) δ 7.50–7.18 (m, 25H, CHAr), 6.38 (d, J = 9.0 Hz, 1H, NH), 6.24 (d, J = 9.3 Hz, 1H, NH), 5.42 (s, 1H, H-7′), 5.34 (t, J = 9.8 Hz, 1H, H-3′), 5.14 (dd, J = 10.4, 8.8 Hz, 1H, H-3), 4.63–4.37 (m, 13H), 4.30 (dd, J = 10.5, 4.9 Hz, 1H), 4.02–3.93 (m, 1H), 3.86 (ddt, J = 18.9, 11.9, 6.1 Hz, 4H), 3.78–3.54 (m, 5H), 3.47–3.38 (m, 2H), 2.73–2.50 (m, 2H), 2.49–2.24 (m, 6H), 1.67–1.42 (m, 9H), 1.41–1.10 (m, 105H, CH_2_ chains), 0.96–0.81 (m, 25H, CH_3_ (chains + TDS)), 0.17 (s, 3H, CH_3_ TDS), 0.10 (s, 3H, CH_3_ TDS).

^13^C NMR (101 MHz, CDCl_3_) δ 171.54, 171.38, 171.37, 171.00, 138.70, 138.61, 138.41, 138.39, 138.00, 137.01, 128.69, 128.61, 128.47, 128.36, 128.34, 128.23, 127.91, 127.89, 127.85, 127.83, 127.82, 127.81, 127.79, 127.77, 127.55, 127.52, 126.21, 101.48, 101.44, 96.19, 78.97, 76.27, 76.10, 76.00, 75.88, 75.81, 75.62, 75.54, 74.67, 74.43, 74.16, 71.97, 71.72, 71.63, 71.61, 71.44, 71.21, 70.97, 70.65, 68.72, 68.29, 66.41, 55.95, 54.61, 41.27, 41.13, 39.81, 39.46, 37.15, 34.60, 34.37, 34.35, 34.05, 33.84, 33.76, 32.07, 29.87, 29.86, 29.83, 29.81, 29.80, 29.78, 29.74, 29.71, 29.69, 29.68, 29.66, 29.48, 25.34, 25.26, 25.24, 25.16, 24.80, 22.80, 22.78, 20.21, 20.19, 18.70, 18.69, 14.23, −1.31, −3.16.

C_127_H_198_N_2_O_17_Si: calcd. mass 2051.45; ESI-MS: *m*/*z* 2074.86 [M+Na]^+^.

#### 3.3.17. 6-*O*-(2-Deoxy-2-((*R*)-3-(benzyloxy)-hexadecanoylamido)-3-*O*-((*R*)-3-(benzyloxy)-hexadecanoyl)-4,6-*O*-benzylidene-β-D-glucopyranosyl)-1-(*O*-thexyldimethylsilyl)-2-deoxy-2-((*R*)-3-(pentadecanoyloxy)-heptadecanoylamido)-3-*O*-((*R*)-3-(benzyloxy)-hexadecanoyl)-4-*O*-benzyl-β-D-glucopyranoside **27**

The crude amine obtained after azide reduction (0.016 mmol) was co-evaporated with toluene and dried in vacuo. To the crude was added double fatty acid **7** (12 mg, 0.024 mmol) and HATU (12 mg, 0.031 mmol), and the mixture was dissolved in dry CH_2_Cl_2_ (1 mL) and DMF (0.2 mL). After adding DIPEA (5.5 µL), the reaction was stirred at room temperature for 4 h. The reaction mixture was then diluted with CH_2_Cl_2_, washed with saturated aqueous NH_4_Cl solution and brine, dried over Na_2_SO_4_, filtered, and concentrated. The crude was purified by size exclusion chromatography (Sephadex^®^ LH-20) (CHCl_3_/MeOH = 1:1) to give **27** (29 mg, 85%).

^1^H NMR (400 MHz, CDCl_3_) δ 7.44–7.13 (m, 25H, CHAr), 6.29 (d, J = 8.9 Hz, 1H, NH), 5.75 (d, J = 9.1 Hz, 1H, NH), 5.39 (s, 1H, H-7′), 5.34–5.25 (m, 1H, H-3′), 5.11 (dd, J = 10.4, 8.5 Hz, 1H, H-3), 5.08–5.00 (m, 1H), 4.69 (d, J = 7.6 Hz, 1H, H-1), 4.60–4.33 (m, 10H), 4.31–4.20 (m, 1H), 3.98–3.74 (m, 5H), 3.73–3.51 (m, 4H), 3.51–3.43 (m, 1H), 3.43–3.34 (m, 1H), 2.64 (dd, J = 15.0, 6.4 Hz, 1H), 2.53 (dd, J = 15.9, 7.0 Hz, 1H), 2.47–2.18 (m, 8H), 1.67–1.39 (m, 4H), 1.39–1.07 (m, 101H, CH_2_ chains), 0.92–0.79 (m, 31H, CH_3_ (chains + TDS)), 0.16 (s, 3H, CH_3_ TDS), 0.13 (s, 3H, CH_3_ TDS).

^13^C NMR (101 MHz, CDCl_3_) δ 173.92, 171.71, 171.39, 171.32, 169.30, 138.69, 138.66, 138.49, 138.04, 137.05, 128.73, 128.55, 128.53, 128.51, 128.42, 128.38, 128.36, 128.29, 128.27, 128.25, 127.91, 127.86, 127.83, 127.81, 127.78, 127.76, 127.63, 127.55, 126.27, 126.25, 126.23, 101.59, 101.49, 96.04, 79.00, 76.33, 76.03, 75.67, 75.59, 74.84, 74.55, 74.23, 71.76, 71.43, 71.26, 71.01, 70.91, 68.76, 68.34, 66.49, 56.36, 54.66, 41.87, 41.36, 39.86, 39.69, 34.68, 34.65, 34.35, 34.14, 33.89, 32.07, 32.05, 29.92, 29.91, 29.90, 29.88, 29.86, 29.83, 29.81, 29.79, 29.77, 29.75, 29.74, 29.71, 29.69, 29.58, 29.51, 29.49, 29.38, 25.45, 25.35, 25.28, 25.20, 25.18, 25.15, 24.88, 22.83, 20.24, 20.22, 18.75, 18.73, 18.70, 18.68, 14.25, −1.37, −3.15.

C_135_H_220_N_2_O_18_Si: calcd. mass 2185.61; ESI-MS: *m*/*z* 2208.58 [M+Na]^+^.

#### 3.3.18. General Procedure for Desilylation

The respective TDS-protected substrate (0.017 mmol) was dissolved in dry THF (1.2 mL) and dry pyridine (0.4 mL). To the solution was added HF-pyridine (0.12 mL), and the reaction was stirred at room temperature for 8 h. The reaction mixture was then diluted with CH_2_Cl_2_, washed with saturated aqueous NH_4_Cl solution and brine, dried over Na_2_SO_4_, filtered, and concentrated. The crude was purified by flash silica gel column chromatography (Toluene/EtOAc = 8:2) to give the corresponding desilylated products.

6-*O*-(2-deoxy-2-((*R*)-3-(benzyloxy)-hexadecanoylamido)-3-*O*-((*R*)-3-(benzyloxy)-hexadecanoyl)-4,6-*O*-benzylidene-β-D-glucopyranosyl)-2-deoxy-2-((*R*)-3-(benzyloxy)-heptadecanoylamido)-3-*O*-((*R*)-3-(benzyloxy)-hexadecanoyl)-4-*O*-benzyl-β-D-glucopyranose **25**. Yield: 84%.

^1^H NMR (400 MHz, CDCl_3_) δ 7.40–7.02 (m, 30H, CHAr), 6.30 (d, J = 8.3 Hz, 1H, NH), 6.20 (d, J = 9.4 Hz, 1H, NH), 5.33 (s, 1H, H-7′), 5.27–5.18 (m, 2H, H-3, H-3′), 4.84 (d, J = 3.5 Hz, 1H, H-1), 4.62 (d, J = 8.4 Hz, 1H, H-1′), 4.52–4.19 (m, 11H), 4.06 (td, J = 9.8, 3.5 Hz, 1H), 3.90 (dd, J = 11.7, 1.9 Hz, 1H), 3.83–3.63 (m, 5H), 3.56 (t, J = 9.4 Hz, 1H), 3.45–3.33 (m, 2H), 3.22 (t, J = 9.6 Hz, 1H), 2.63–2.42 (m, 2H), 2.39–2.04 (m, 5H), 1.53–1.30 (m, 8H, CH_2_ chains), 1.29–1.01 (m, 99H, CH, CH_2_ chains), 0.86–0.73 (m, 12H, CH_3_ chains).

^13^C NMR (101 MHz, CDCl_3_) δ 172.00, 171.97, 171.51, 171.46, 138.76, 138.72, 138.70, 137.76, 137.65, 137.06, 128.76, 128.64, 128.61, 128.56, 128.53, 128.48, 128.45, 128.42, 128.40, 128.35, 128.33, 128.00, 127.98, 127.96, 127.94, 127.91, 127.90, 127.87, 127.85, 127.79, 127.61, 127.59, 126.30, 102.28, 101.64, 91.51, 79.04, 76.60, 76.56, 75.69, 75.66, 74.39, 73.50, 71.94, 71.65, 71.55, 71.53, 71.39, 71.20, 70.64, 69.41, 68.81, 66.71, 55.36, 52.75, 42.10, 41.74, 40.00, 39.85, 34.64, 34.50, 34.37, 33.94, 32.11, 29.92, 29.90, 29.89, 29.85, 29.84, 29.81, 29.55, 25.40, 25.38, 25.35, 25.31, 22.87, 14.29.

C_119_H_180_N_2_O_17_: calcd. mass 1909.33; ESI-MS: *m*/*z* 1932.42 [M+Na]^+^.

6-*O*-(2-deoxy-2-((*R*)-3-(benzyloxy)-hexadecanoylamido)-3-*O*-((*R*)-3-(benzyloxy)-hexadecanoyl)-4,6-*O*-benzylidene-β-D-glucopyranosyl)-2-deoxy-2-((*R*)-3-(pentadecanoyloxy)-heptadecanoylamido)-3-*O*-((*R*)-3-(benzyloxy)-hexadecanoyl)-4-*O*-benzyl-D-glucopyranose **28**; Yield: 75%.

^1^H NMR (400 MHz, CDCl_3_) δ 7.47–7.12 (m, 25H. CHAr), 6.38 (d, J = 8.5 Hz, 1H, NH), 5.92 (d, J = 9.2 Hz, 1H, NH), 5.42 (s, 1H, H-7′), 5.35–5.24 (m, 2H, H-3, H-3′), 5.12 (dd, J = 7.4, 4.9 Hz, 1H), 4.93 (d, J = 3.5 Hz, 1H, H-1), 4.71 (d, J = 8.4 Hz, 1H, H-1′), 4.63–4.27 (m, 8H, CH_2_Ph), 4.12–3.98 (m, 2H), 3.95–3.72 (m, 4H), 3.65 (t, J = 9.4 Hz, 1H), 3.55–3.40 (m, 2H), 3.29 (t, J = 9.5 Hz, 1H), 2.71–2.53 (m, 2H), 2.48–2.19 (m, 6H), 1.96 (s, 1H, OH), 1.70–1.40 (m, 10H, CH_2_ chains), 1.39–1.04 (m, 106H, CH_2_ chains), 0.89 (t, J = 6.8 Hz, 15H, CH_3_ chains).

^13^C NMR (101 MHz, CDCl_3_) δ 173.30, 172.30, 171.86, 171.39, 169.80, 138.64, 137.63, 137.57, 136.98, 128.74, 128.51, 128.45, 128.37, 128.34, 128.29, 127.94, 127.88, 127.86, 127.84, 127.82, 127.58, 127.56, 126.24, 102.26, 101.59, 91.25, 78.98, 77.48, 77.16, 76.85, 76.77, 76.53, 75.63, 75.55, 74.47, 73.53, 71.86, 71.43, 71.23, 71.15, 71.13, 71.01, 70.65, 69.32, 68.75, 66.65, 55.23, 52.88, 41.55, 39.93, 39.80, 34.64, 34.57, 34.43, 34.37, 33.83, 32.06, 32.04, 29.87, 29.86, 29.84, 29.82, 29.80, 29.77, 29.75, 29.73, 29.71, 29.59, 29.53, 29.50, 29.38, 25.40, 25.35, 25.28, 25.26, 25.20, 22.82, 14.25.

C_127_H_202_N_2_O_18_: calcd. mass 2043.50; ESI-MS: *m*/*z* 2066.72 [M+Na]^+^.

6-*O*-(2-deoxy-2-((*R*)-3-(benzyloxy)-hexadecanoylamido)-3-*O*-((*R*)-3-(benzyloxy)-hexadecanoyl)-4-(*O*-dibenzylphosphoryl)-6-*O*-benzyl-β-D-glucopyranosyl)-2-deoxy-2-((*R*)-3-(pentadecanoyloxy)-heptadecanoylamido)-3-*O*-((*R*)-3-(benzyloxy)-hexadecanoyl)-4-*O*-benzyl-D-glucopyranose **32**. Yield: 73%.

^1^H NMR (400 MHz, CDCl_3_) δ 7.38–7.12 (m, 35H, CHAr), 6.22 (d, J = 7.9 Hz, 1H, NH), 5.87 (d, J = 9.3 Hz, 1H, NH), 5.38 (t, J = 9.7 Hz, 1H, H-3′), 5.28 (t, J = 9.7 Hz, 1H, H-3), 5.17–5.08 (m, 1H), 4.99–4.83 (m, 6H, H-1, H-1′, (C*H*_2_PhO)_2_PO-), 4.60–4.31 (m, 11H, CH_2_Ph, H-4′), 4.12–4.02 (m, 1H, H-2), 3.99–3.90 (m, 2H), 3.87–3.69 (m, 5H), 3.68–3.45 (m, 4H), 3.25 (t, J = 9.4 Hz, 1H, H-4), 2.62–2.24 (m, 8H), 2.22–2.14 (m, 2H), 1.80 (s, 2H), 1.65–1.40 (m, 3H), 1.25 (d, J = 9.5 Hz, 148H, CH_2_ chains), 0.88 (t, J = 6.7 Hz, 15H, CH_3_ chains).

^13^C NMR (101 MHz, CDCl_3_) δ 173.27, 172.24, 172.04, 171.30, 169.73, 138.78, 138.70, 138.11, 137.83, 137.62, 135.71, 128.67, 128.53, 128.46, 128.38, 128.31, 128.16, 128.11, 127.96, 127.90, 127.86, 127.83, 100.58, 91.28, 76.34, 75.57, 74.52, 74.31, 73.83, 73.60, 71.45, 69.74, 69.66, 68.84, 55.57, 52.85, 41.53, 39.95, 39.00, 34.65, 34.42, 34.30, 34.01, 32.08, 29.87, 29.82, 29.52, 25.36, 22.83, 14.25.

^31^P NMR (162 MHz, CDCl_3_) δ −2.14.

C_141_H_217_N_2_O_21_P: calcd. mass 2305.57; ESI-MS: *m*/*z* 2328.67 [M+Na]^+^.

#### 3.3.19. General Procedure for Phosphorylation of the Anomeric Position

To the respective substrate with a free anomeric hydroxyl group (0.01 mmol) was added dry THF (1 mL), and the solution was cooled to −70 °C. Then, LiHMDS (0.03 mmol) was added, and the reaction was stirred at −70 °C for 30 min. Thereafter, tetrabenzyl pyrophosphate (0.02 mmol) was added, and stirring continued for 1 h while warming from −70 °C to 0 °C. The reaction mixture was diluted with CH_2_Cl_2_, washed with saturated aqueous NH_4_Cl solution and brine, dried over Na_2_SO_4_, filtered, and concentrated. The crude was purified by flash silica gel column chromatography (Toluene/EtOAc = 9:1) to give the corresponding phosphorylated products.

6-*O*-(2-deoxy-2-((*R*)-3-(benzyloxy)-hexadecanoylamido)-3-*O*-((*R*)-3-(benzyloxy)-hexadecanoyl)-4,6-*O*-benzylidene-β-D-glucopyranosyl)-1-*O*-(dibenzylphosphoryl)-2-deoxy-2-((*R*)-3-(benzyloxy)-heptadecanoylamido)-3-*O*-((*R*)-3-(benzyloxy)-hexadecanoyl)-4-*O*-benzyl-α-D-glucopyranoside **26**. Yield: 63%.

^1^H NMR (400 MHz, CDCl_3_) δ 7.44–7.19 (m, 40H, CHAr), 7.02 (d, J = 8.9 Hz, 1H, NH), 6.24 (d, J = 8.7 Hz, 1H, NH), 5.68 (dd, J = 5.4, 3.3 Hz, 1H, H-1), 5.40 (s, 1H, H-7′), 5.34–5.23 (m, 2H, H-3, H-3′), 5.08–4.89 (m, 5H), 4.70 (d, J = 8.4 Hz, 1H, H-1′), 4.61–4.24 (m, 17H), 4.08–3.95 (m, 1H), 3.87–3.67 (m, 5H), 3.62 (t, J = 9.5 Hz, 1H), 3.54 (t, J = 9.6 Hz, 1H), 3.40–3.32 (m, 1H), 2.66 (dd, J = 15.0, 6.6 Hz, 1H), 2.54 (dd, J = 16.1, 7.3 Hz, 1H), 2.46–2.11 (m, 6H), 2.03–1.93 (m, 1H), 1.86–1.68 (m, 2H), 1.60–1.41 (m, 2H), 1.37–1.07 (m, 95H, CH_2_ chains), 0.89 (t, J = 6.9 Hz, 12H, CH_3_ chains).

^13^C NMR (101 MHz, CDCl_3_) δ 172.04, 171.71, 171.45, 171.36, 138.78, 138.72, 138.59, 138.55, 137.41, 137.10, 128.94, 128.92, 128.84, 128.83, 128.62, 128.57, 128.55, 128.53, 128.51, 128.46, 128.41, 128.38, 128.37, 128.34, 128.32, 128.29, 128.26, 128.21, 128.13, 128.10, 127.94, 127.91, 127.86, 127.83, 127.80, 127.69, 127.64, 127.49, 126.24, 101.49, 100.99, 79.16, 76.09, 75.67, 75.43, 75.34, 74.92, 71.54, 71.27, 71.09, 71.04, 70.11, 70.06, 70.01, 66.56, 54.37, 52.32, 41.46, 41.24, 39.94, 39.81, 34.70, 34.35, 34.08, 32.07, 29.87, 29.85, 29.83, 29.81, 29.78, 29.51, 25.29, 22.83, 14.27.

^31^P NMR (162 MHz, CDCl_3_) δ −3.10.

C_133_H_193_N_2_O_20_P: calcd. mass 2169.39; ESI-MS: *m*/*z* 2192.68 [M+Na]^+^.

6-*O*-(2-deoxy-2-((*R*)-3-(benzyloxy)-hexadecanoylamido)-3-*O*-((*R*)-3-(benzyloxy)-hexadecanoyl)-4,6-*O*-benzylidene-β-D-glucopyranosyl)-1-(*O*-dibenzylphosphoryl)-2-deoxy-2-((*R*)-3-(pentadecanoyloxy)-heptadecanoylamido)-3-*O*-((*R*)-3-(benzyloxy)-hexadecanoyl)-4-*O-*benzyl-α-D-glucopyranose **29**. Yield: 65%.

^1^H NMR (400 MHz, CDCl_3_) δ 7.43–7.12 (m, 35H, CHAr), 6.99 (d, J = 9.0 Hz, 1H, NH-1), 5.99 (d, J = 8.6 Hz, 1H NH-2), 5.63 (dd, J = 5.5, 3.2 Hz, 1H, H-1), 5.38 (s, 1H, H-7′), 5.33–5.18 (m, 2H, H-3′, H-3), 5.15–4.97 (m, 5H, ((CH_2_Ph)O)_2_PO-), H-3′ C17 chain), 4.67 (d, J = 8.4 Hz, 1H, H-1′), 4.60–4.34 (m, 8H, CH_2_Ph), 4.28 (dd, J = 10.5, 4.9 Hz, 1H, H-6′a), 4.19 (tt, J = 8.4, 4.3 Hz, 1H, H-2), 4.05–3.97 (m, 2H, H-5, H-2′), 3.87–3.68 (m, 6H, H-6′b, H-6a,b, H-3′ C16 chains), 3.60 (t, J = 9.4 Hz, 1H, H-4′), 3.52 (t, J = 9.6 Hz, 1H, H-4), 3.36 (dt, J = 9.7, 4.9 Hz, 1H, H-5′), 2.65 (dd, J = 15.0, 6.6 Hz, 1H), 2.55 (dd, J = 16.0, 7.3 Hz, 1H), 2.50–2.35 (m, 3H), 2.34–2.18 (m, 4H), 2.17–2.07 (m, 1H), 1.66–1.37 (m, 5H, CH_2_ chains), 1.36–0.99 (m, 112H, CH_2_ chains), 0.88 (t, J = 6.8 Hz, 15H, CH_3_ chains).

^13^C NMR (101 MHz, CDCl_3_) δ 173.35, 172.47, 171.71, 171.34, 169.96, 138.74, 138.69, 138.54, 137.41, 137.07, 135.56, 135.50, 128.95, 128.94, 128.85, 128.83, 128.61, 128.53, 128.41, 128.33, 128.24, 128.18, 128.14, 127.91, 127.89, 127.84, 127.81, 127.65, 127.48, 126.22, 101.47, 100.99, 96.05, 79.13, 76.07, 75.65, 75.40, 75.20, 74.97, 73.64, 72.46, 71.92, 71.44, 71.25, 71.01, 70.58, 70.14, 70.11, 70.09, 70.05, 68.74, 66.53, 54.35, 52.60, 52.51, 41.41, 41.24, 39.92, 39.73, 34.68, 34.50, 34.32, 34.27, 33.94, 32.05, 29.87, 29.85, 29.84, 29.80, 29.78, 29.76, 29.75, 29.50, 25.33, 25.27, 25.13, 22.82, 14.27.

^31^P NMR (162 MHz, CDCl_3_) δ −3.05.

C_141_H_215_N_2_O_21_P: calcd. mass 2303.56; ESI-MS: *m*/*z* 2326.52 [M+Na]^+^.

#### 3.3.20. 6-*O*-(2-Deoxy-2-((*R*)-3-(benzyloxy)-hexadecanoylamido)-3-*O*-((*R*)-3-(benzyloxy)-hexadecanoyl)-6-*O*-benzyl-β-D-glucopyranosyl)-1-*O*-thexyldimethylsilyl-2-deoxy-2-((*R*)-3-(pentadecanoyloxy)-heptadecanoylamido)-3-*O*-((*R*)-3-(benzyloxy)-hexadecanoyl)-4-*O*-benzyl-β-D-glucopyranoside **30**

Dry CH_2_Cl_2_ (4 mL) was added to a mixture of compound **27** (87 mg, 0.040 mmol) with activated MS4Å, and the suspension was stirred at room temperature for 3 h before cooling to -78 °C. Then triethylsilane (51 µL, 0.32 mmol) and triflic acid (35 µL, 0.40 mmol) were added successively to the reaction mixture. After stirring the reaction for 3 h at -78 °C, triethylamine (58 µL) and MeOH (0.4 mL) were added, and stirring continued for 10 min. The reaction was then warmed to room temperature, diluted with CH_2_Cl_2_, washed with saturated aqueous NaHCO_3_ solution and brine, dried over Na_2_SO_4_, filtered, and concentrated. The crude was purified by flash silica gel column chromatography (Toluene/EtOAc = 9:1) to give **30** (62 mg, 70%).

^1^H NMR (400 MHz, CDCl_3_) δ 7.39–7.12 (m, 25H, CHAr), 6.16 (d, J = 8.8 Hz, 1H, NH-1′), 5.68 (d, J = 9.2 Hz, 1H, NH-1), 5.07 (m, 3H, H-3′, H-3, H-3′ C17 chain), 4.64 (d, J = 7.8 Hz, 1H, H-1), 4.59–4.38 (m, 10H), 3.91–3.59 (m, 8H), 3.55 (t, J = 9.1 Hz, 1H), 3.46 (dt, J = 9.6, 4.2 Hz, 2H), 2.62 (dd, J = 14.8, 7.6 Hz, 1H), 2.56–2.16 (m, 8H), 1.68–1.47 (m, 3H), 1.25 (dd, J = 7.3, 3.5 Hz, 125H, CH_2_ chains), 0.94–0.77 (m, 27H, CH_3_ chains, CH_3_ TDS), 0.14 (s, 3H, CH_3_ TDS), 0.11 (s, 3H, CH_3_ TDS).

^13^C NMR (101 MHz, CDCl_3_) δ 173.88, 172.41, 171.74, 171.23, 169.25, 138.69, 138.53, 138.34, 138.04, 137.99, 128.68, 128.57, 128.47, 128.01, 127.93, 127.87, 127.83, 127.81, 127.78, 127.61, 101.09, 96.08, 76.32, 76.12, 76.02, 75.96, 75.56, 75.05, 74.70, 74.22, 74.20, 73.81, 71.41, 71.30, 71.08, 70.97, 70.89, 70.53, 68.10, 56.44, 53.77, 41.82, 41.54, 39.91, 39.65, 34.68, 34.35, 34.30, 34.12, 33.98, 32.07, 29.88, 29.86, 29.85, 29.83, 29.81, 29.80, 29.78, 29.51, 25.45, 25.34, 25.29, 25.15, 24.85, 22.83, 20.20, 18.71, 14.30, −1.35, −3.17.

C_135_H_222_N_2_O_18_Si: calcd. mass 2187.63; ESI-MS: *m*/*z* 2210.42 [M+Na]^+^.

#### 3.3.21. 6-*O*-(2-Deoxy-2-((*R*)-3-(benzyloxy)-hexadecanoylamido)-3-*O*-((*R*)-3-(benzyloxy)-hexadecanoyl)-4-(*O*-dibenzylphosphoryl)-6-*O*-benzyl-β-D-glucopyranosyl)-1-(*O*-thexyldimethylsilyl)-2-deoxy-2-((*R*)-3-(pentadecanoyloxy)-heptadecanoylamido)-3-*O*-((*R*)-3-(benzyloxy)-hexadecanoyl)-4-*O*-benzyl-D-glucopyranose **31**

The mixture of Compound **30** (13 mg, 0.006 mmol) and *1H*-tetrazole (4 mg, 0.06 mmol) was dissolved in dry CH_2_Cl_2_ (0.6 mL) and MeCN (0.1 mL), and the solution was cooled to 0 °C. Dibenzyl *N*,*N*-diisopropylphosphoramidite (5 µL, 0.015 mmol) was added, and the reaction was stirred at room temperature for 2 h. Then, *m*CPBA (4 mg, 0.023 mmol) was added and continued stirring for 30 min. The reaction mixture was diluted with CH_2_Cl_2_, washed with saturated aqueous NaHCO_3_ solution and brine; dried over Na_2_SO_4_, filtered, and concentrated. The crude was purified by size exclusion chromatography (Sephadex^®^ LH-20) (CHCl_3_/MeOH = 1:1) to give **31** (13 mg, 87%).

^1^H NMR (400 MHz, CDCl_3_) δ 7.38–7.13 (m, 35H, CHAr), 6.06 (d, J = 8.2 Hz, 1H, NH), 5.64 (d, J = 9.3 Hz, 1H, NH), 5.42 (t, J = 9.7 Hz, 1H, H-3′), 5.16–5.00 (m, 2H, H-3, H-3′ C17 chain), 4.92–4.84 (m, 4H, ((CH_2_Ph)O)_2_PO-)), 4.65 (d, J = 8.3 Hz, 1H, H-1′), 4.61 (d, J = 7.7 Hz, 1H, H-1), 4.55–4.31 (m, 12H, CH_2_Ph), 3.91–3.43 (m, 8H), 2.60–2.10 (m, 9H), 1.81–1.67 (m, 2H), 1.67–1.38 (m, 4H), 1.38–1.03 (m, 95H, CH_2_ chains), 0.97–0.73 (m, 32H, CH_3_ chains, CH_3_ TDS), 0.15 (s, 3H, CH_3_ TDS), 0.10 (s, 3H, CH_3_ TDS).

^13^C NMR (101 MHz, CDCl_3_) δ 173.87, 171.73, 171.50, 171.39, 169.23, 138.82, 138.72, 138.66, 138.45, 137.97, 135.80, 128.65, 128.44, 128.39, 128.14, 128.07, 127.98, 127.83, 127.71, 127.64, 127.59, 100.60, 96.12, 76.25, 76.15, 75.56, 75.18, 74.64, 74.32, 74.24, 73.53, 72.78, 71.41, 71.07, 71.00, 70.92, 69.75, 69.69, 69.62, 69.57, 68.88, 68.63, 56.50, 55.24, 41.85, 41.55, 39.66, 39.08, 34.69, 34.37, 34.15, 32.07, 29.87, 29.82, 29.51, 25.46, 25.40, 25.35, 25.16, 22.83, 20.23, 18.72, 14.24, −1.36, −3.15.

^31^P NMR (162 MHz, CDCl_3_) δ −2.11.

C_149_H_235_N_2_O_21_PSi: calcd. mass 2447.69; ESI-MS: *m*/*z* 2470.80 [M+Na]^+^.

#### 3.3.22. General Procedure for Hydrogenolysis

The respective substrate (0.027 mmol) was dissolved in a mixture of CH_2_Cl_2_ (1.2 mL) and MeOH (1.2 mL), and to the solution was added AcOH (0.12 mL) and Pd(OH)_2_/C catalyst (120 mg), and the reaction was stirred for 2 days under a hydrogen gas atmosphere using a balloon at room temperature. The reaction mixture was neutralized with Et_3_N (0.36 mL), and the palladium was filtered off using a micropore membrane filter. The filtrate was co-evaporated with toluene and concentrated in a rotary evaporator. The crude was purified by size exclusion chromatography (Sephadex^®^ LH-20) (CHCl_3_/MeOH = 1:1) to obtain the corresponding final fully deprotected lipid A compound.

Tetra C-1; quantitative yield.

^1^H NMR (400 MHz, CDCl_3_/MeOD) δ 5.37–5.28 (m, 1H), 5.21 (s, 1H), 5.08–4.93 (m, 4H), 4.89–4.75 (m, 1H), 4.66 (d, J = 8.5 Hz, 1H), 4.38 (dd, J = 14.6, 9.5 Hz, 1H), 3.68–3.59 (m, 1H), 3.46 (t, J = 9.5 Hz, 1H), 3.39–3.28 (m, 9H), 3.26–3.17 (m, 5H), 3.01 (q, J = 7.3 Hz, 1H), 2.87 (q, J = 7.2 Hz, 16H), 2.43–1.99 (m, 10H), 1.95–1.79 (m, 1H), 1.50–0.89 (m, 90H), 0.74 (t, J = 6.8 Hz, 15 H).

^13^C NMR (101 MHz, CDCl_3_/MeOD) δ 173.60, 173.27, 172.76, 172.58, 129.88, 129.61, 75.75, 68.85, 68.75, 68.37, 68.29, 68.15, 67.99, 62.42, 61.18, 52.52, 42.06, 36.98, 31.81, 29.65, 29.62, 29.59, 29.56, 29.54, 29.52, 29.47, 29.45, 29.26, 29.24, 29.22, 29.18, 25.49, 25.45, 22.55, 13.88, 10.81, 7.21.

^31^P NMR (162 MHz, CDCl_3_/MeOD) δ 2.81.

HR-MS: C_77_H_147_N_2_O_20_P: calcd. mass 1451.03; ESI-qTOF MS: *m*/*z* 1450.0225 [M-H]^−^.

Penta C-1; quantitative yield.

^1^H NMR (400 MHz, CDCl_3_) δ 5.27–5.13 (m, 1H), 5.05–4.93 (m, 2H), 4.79 (t, J = 9.8 Hz, 2H), 4.58 (d, J = 8.5 Hz, 1H), 3.90–3.70 (m, 3H), 3.68–3.57 (m, 2H), 3.53 (d, J = 12.9 Hz, 2H), 3.44 (t, J = 9.5 Hz, 3H), 3.37–3.26 (m, 1H), 3.25–3.18 (m, 9H), 2.99 (q, J = 7.5 Hz, 6H), 2.85 (q, J = 7.2 Hz, 21H), 2.39–1.96 (m, 21H), 1.86 (d, J = 5.1 Hz, 2H), 1.52–1.00 (m, 80H), 0.72 (t, J = 6.7 Hz, 15H).

^13^C NMR (101 MHz, CDCl_3_) δ 173.73, 173.54, 173.01, 172.80, 171.19, 162.64, 130.06, 129.77, 71.03, 68.80, 68.67, 68.35, 68.09, 51.95, 51.79, 51.54, 46.08, 42.54, 42.05, 41.34, 37.47, 37.17, 36.64, 35.88, 34.54, 34.18, 34.14, 34.07, 31.99, 31.97, 29.85, 29.83, 29.81, 29.78, 29.75, 29.73, 29.72, 29.70, 29.44, 29.42, 27.25, 27.21, 25.94, 25.80, 25.73, 25.61, 25.52, 25.37, 25.12, 24.99, 24.98, 22.73, 14.12, 14.10, 11.03, 8.58.

^31^P NMR (162 MHz, CDCl_3_) δ 1.99.

HR-MS: C_92_H_175_N_2_O_21_P: calcd. mass 1675.24; ESI-qTOF MS: *m*/*z* 1674.2378 [M-H]^−^.

Penta C-4′; quantitative yield.

^1^H NMR (400 MHz, CDCl_3_) δ 5.09–5.04 (m, 1H), 4.96–4.84 (m, 2H), 4.77 (d, J = 3.5 Hz, 0H), 4.11 (s, 1H), 3.96 (q, J = 10.0 Hz, 1H), 3.91–3.82 (m, 1H), 3.82–3.74 (m, 2H), 3.74–3.66 (m, 1H), 3.56–3.43 (m, 1H), 3.32–3.16 (m, 1H), 2.89 (q, J = 7.3 Hz, 7H), 2.74 (q, J = 7.3 Hz, 5H), 2.63–2.53 (m, 1H), 2.26–2.08 (m, 3H), 2.02 (q, J = 6.3 Hz, 3H), 1.94 (t, J = 7.7 Hz, 2H), 1.82–1.67 (m, 3H), 1.43–1.26 (m, 6H), 1.26–0.79 (m, 120H), 0.61 (t, J = 6.7 Hz, 15H).

^13^C NMR (101 MHz, CDCl_3_) δ 173.52, 173.05, 172.81, 172.11, 170.69, 101.53, 91.10, 75.49, 74.04, 73.78, 70.81, 70.25, 70.05, 68.84, 68.35, 68.22, 60.12, 53.73, 51.81, 43.49, 42.17, 41.92, 41.75, 40.80, 36.98, 36.87, 34.14, 33.80, 31.61, 29.38, 29.03, 25.32, 25.14, 24.91, 24.74, 22.33, 13.53, 10.56.

^31^P NMR (162 MHz, CDCl_3_) δ 4.90.

HR-MS: C_92_H_175_N_2_O_21_P: calcd. mass 1675.24; ESI-qTOF MS: *m*/*z* 1674.2343 [M-H]^−^.

## 4. Conclusions

In this work, we underscore the therapeutic potential of microbiota-inspired glycolipids as modulators of innate immunity. By synthesizing and computationally evaluating a panel of *Bacteroides fragilis*-derived lipid A analogs, we identified **Tetra C-1** (Figure 2) as a promising TLR4 antagonist with superior binding characteristics compared to Eritoran, a well-established antagonist that we used as a benchmark. This indicates a potentially superior antagonistic profile, meriting further investigation. As a next step, we will evaluate our designed *Bacteroides fragilis*-derived glycolipids both in vitro and in vivo, using established cell line models and murine models of LPS-induced inflammation—systems previously employed to assess the immunomodulatory effects of *B. fragilis*-derived lipids. These models will enable quantification of cytokine levels (e.g., TNF-α, IL-6), assessment of epithelial barrier integrity, and analysis of innate immune modulation. A direct comparison with Eritoran and other known TLR4 ligands as reference compounds in these models could validate our findings and support the development of *B. fragilis*-derived glycolipids as therapeutic agents for inflammatory diseases and immunomodulation. Our findings pave the way for the rational design of novel anti-inflammatory agents and vaccine adjuvants, leveraging the immunomodulatory properties of commensal-derived molecules to target TLR4-mediated pathways in inflammatory diseases. This study marks a further advance toward the development of novel therapeutics targeting TLR4 in pursuit of next-generation immunomodulators and vaccine adjuvants inspired by the human microbiome.

## Figures and Tables

**Table 1:** Screening of conditions for condensation of fatty acid **4** with partially deprotected disaccharide **22**.

Entry	Reaction Reagents (Equivalents)	Equivalents of Fatty Acids	Reaction Concentration (M)	Yield (%)
1	HBTU (6.0 eq.), DIPEA (10 eq.), DMAP (3.0 eq.)	4.5	0.05	63
2	HATU (6.0 eq.), DIPEA (10 eq.), DMAP (3.0 eq.)	4.5	0.05	64
3	EDC HCl (6.0 eq.), DMAP (1.5 eq.)	4.5	0.05	66
4	EDC HCl (10. eq.), DMAP (1.5 eq.)	4.0	0.1	75

**Table 2:** MM-GBSA ranking of the antagonist-like binding mode of Tetra-C1, Eritoran, and lipid IVa. Autodock Vina score and disaccharide RMSD value are also reported.

Compound	RMSD * (Å)	Autodock Score (kcal/mol)	MM-GBSA (kcal/mol)
Tetra-C1	0.52	−7.65	−129.79
lipid IVa	0.80	−7.84	−126.09
Eritoran	3.51	−7.69	−83.74

* reference structure = lipid IVa X-ray structure PDB ID 2E59.

## Data Availability

Data are contained within this article or in Supplementary Materials.

## Supplementary Materials

The following supporting information can be downloaded at https://www.mdpi.com/article/10.3390/molecules30193927/s1. NMR and MS spectra of synthesized compounds.

## Abbreviations

LPS, lipopolysaccharides; TLRs, Toll-like receptors; TLR1-2-4, Toll-like receptor 1, 2 or 4; MD-2, Myeloid Differentiation factor 2; MPLA, monophosphoryl lipid A; Th1, T-helper; CD4, Cluster of Differentiation 4; PSA, polysaccharide A; IL-10, Interleukin 10; NMR, Nuclear Magnetic Resonance; LC, Liquid chromatography; MS, Mass Spectrometry; DMAP, Dimethylaminopyridine; Py, Pyridine; AcOH, Acetic Acid; DMF, Dimethylformamide; TDSCl, Thexyl-dimethyl silyl chloride; pTSA, para-Toluensulfonic acid; THF, Tetrahydrofuran; NIS, N-Iodosuccinimide; TMSOTf, Trimethylsilyl trifluoromethanesulfonate; DCC, Dicyclohexylcarbodiimide; BINAP, 2,2′-bis(diphenylphosphino)-1,1′-binaphthyl; MTPA, α-methoxy-α-trifluoromethylphenylacetic acid; DIC, Diisopropylcarbodiimide; TFA, trifluoroacetic acid; LiHDMS, Lithium bis(trimethylsilyl)amide; HBTU, Hexafluorophosphate Benzotriazole Tetramethyl Uronium; DIPEA, N,N-Diisopropylethylamine; HATU, 1-[Bis(dimethylamino)methylene]-1H-1,2,3-triazolo [4,5-b]pyridinium 3-oxide hexafluorophosphate; EDC, 1-Ethyl-3-(3-dimethylaminopropyl)carbodiimide; PDB, Protein DataBank; RMSD, Root mean square deviation; MM-GBSA, Molecular Mechanics-Generalized Born Surface Area; EtOAc, Ethyl acetate; DCM, dichloromethane; TLC, Thin-Layer Chromatography; ESI, ElectroSpay Ionization; mCPBA, meta-chloroperbenzoic acid.
