# Supplementary material for: Synthesis and Docking Studies of Glycolipids Inspired by Bacteroides fragilis Lipid A
Source: Molecules. 2025 Sep 30;30(19):3927. doi: 10.3390/molecules30193927 (PMC12525944; doi:10.3390/molecules30193927)
Supplement: Supplementary file 1 [file molecules-30-03927-s001.zip › molecules-3837152-supplementary.pdf]

molecules

Article

Synthesis and Docking Studies of Glycolipids Inspired by Bacteroides fragilis Lipid A

Davie Kenneth [1,†], Cristina Manuela Santi [1,2,†], Francesca Tanda [1], Alessia Izzo [1], Monica Civera [1], Giuseppe D'Orazio [1,*] and Luigi Lay [1,*]

Supplementary Materials

- **Figures S1 – S13**
- **Table S1 – S13**
- **NMR and MS spectra of synthesized compounds.**

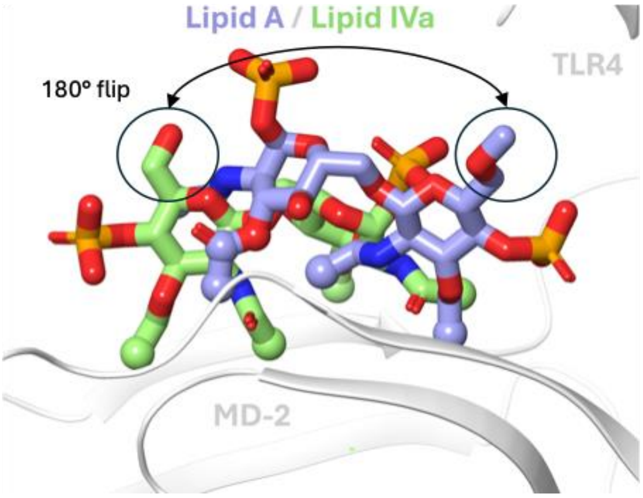

Figure S1. Alignment of the X-ray structures of the hMD-2 binding site (grey) bound to the agonist (purple, 3FXI.pdb) and antagonist (green, 2E59.pdb) ligands. Only the disaccharide moieties are shown while the acyl chains have been omitted for clarity. The RMSD between the heavy atoms of the disaccharide core is 7.17 Å.

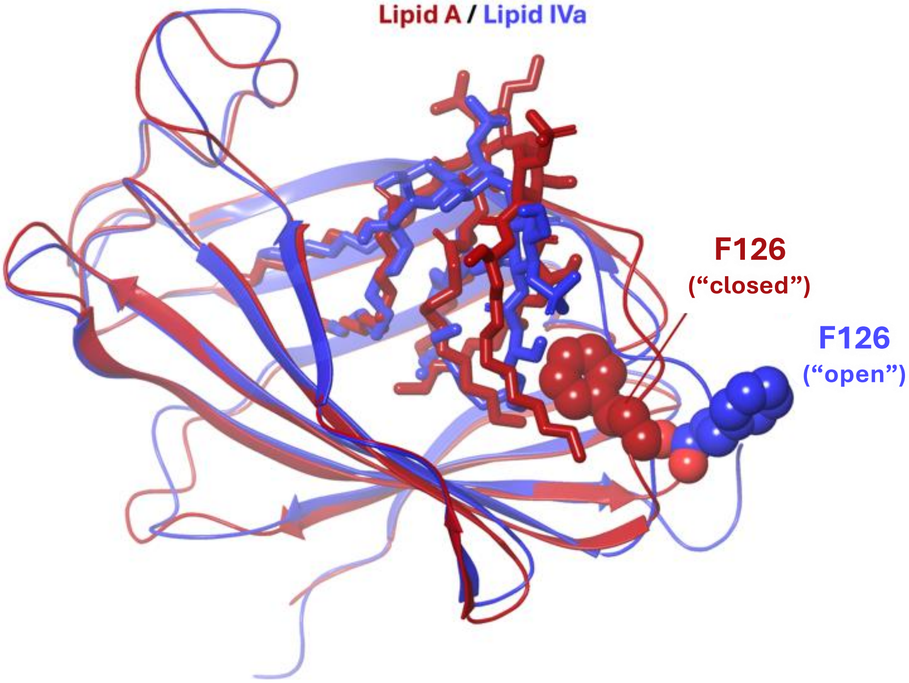

Figure S2. Superposition of MD-2 protein X-ray structure in the agonist (red, 3FXI.pdb) and antagonist conformation (blue, 2E59.pdb). F126 dual conformation is highlighted as CPK spheres and ligands as tube representation.

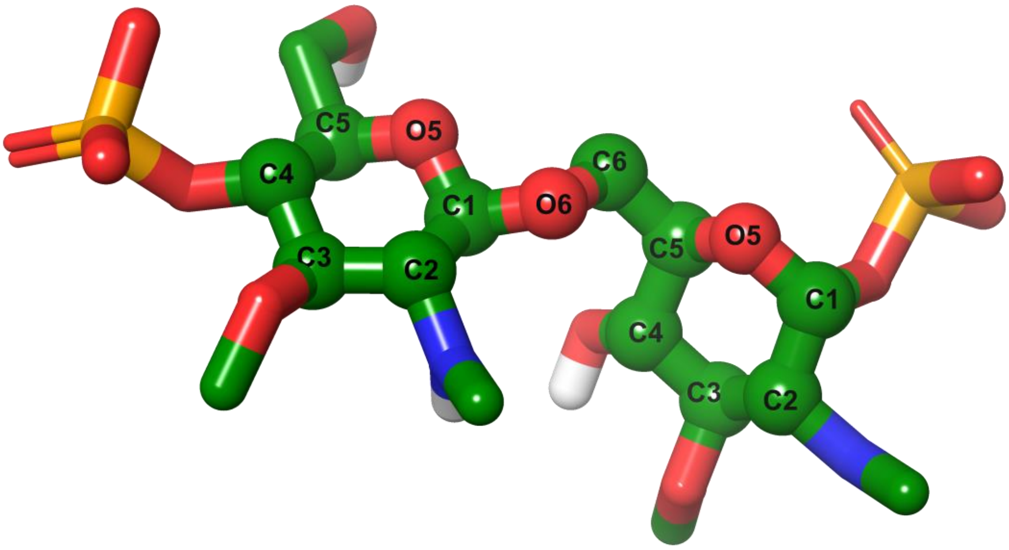

Figure S3. Selection of the heavy atoms of the disaccharide used for the RMSD calculation. The selected atoms are represented as spheres.

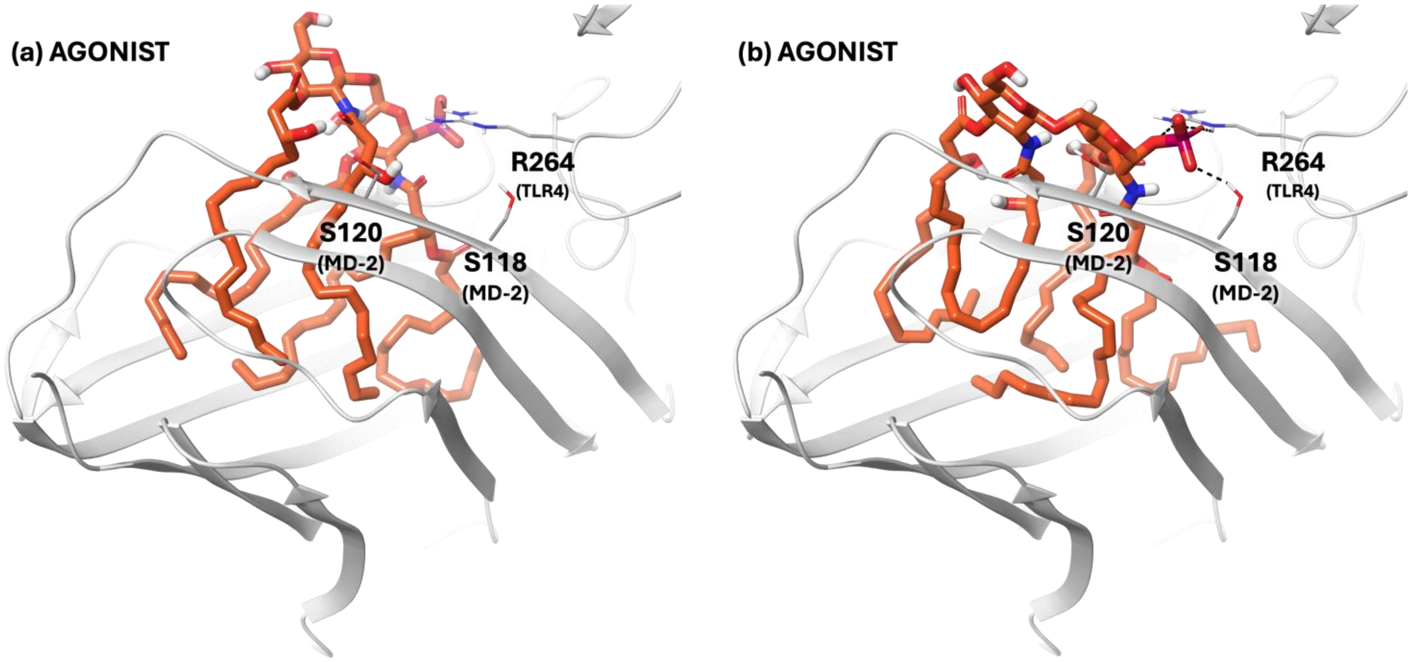

Figure S4. Representative docking poses of **Penta-C1** (shown in orange) in the agonist MD-2/TLR4 model. Volume-overlap clustering identified two distinct families of docking poses, shown in panels (a–b).

(a) Representative binding mode of cluster 1. The disaccharide moiety is poorly aligned with the lipid A X-ray reference (average RMSD = 8.82 ± 0.66 Å). All acyl chains are buried within the MD-2 pocket, with no solvent-exposed chain, a conformation incompatible with an agonist-like pose. A salt bridge with residue R264 is observed.

(b) Representative binding mode of cluster 2. Similarly, the disaccharide shows suboptimal alignment (average RMSD = 6.21 ± 0.19 Å), and all acyl chains are fully buried, again inconsistent with an agonist-like conformation. In this cluster, the ligand establishes both the salt bridge with R264 and a hydrogen bond with residue S118.

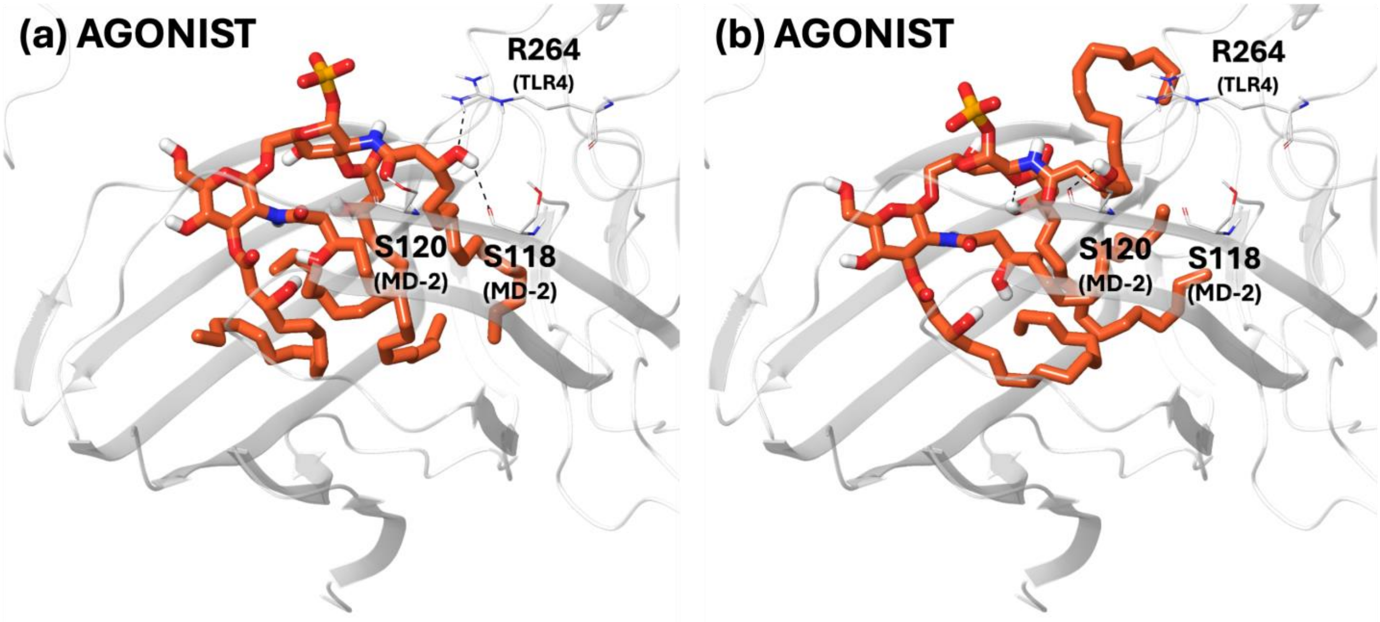

Figure S5. Representative docking poses of **Tetra-C1** (shown in orange) in the agonist MD-2/TLR4 model. Volume-overlap clustering identified two distinct binding families, shown in panels (a–b).

(a) Representative binding mode of cluster 1. The disaccharide moiety is poorly aligned with the lipid A X-ray reference (average RMSD = 8.84 ± 0.72 Å). All acyl chains are buried within the MD-2 pocket, with no solvent-exposed chain, a conformation incompatible with an agonist-like pose. The ligand forms hydrogen bonds with R264 (through one acyl chain oxygen rather than the phosphate group) and with residue S118.

(b) Representative binding mode of cluster 2. The disaccharide shows the poorest alignment (average RMSD = 11.10 ± 0.63 Å). One acyl chain protrudes from the MD-2 cavity in an orientation incompatible with the canonical agonist-like conformation. No key interactions with R264 or other stabilizing residues are observed.

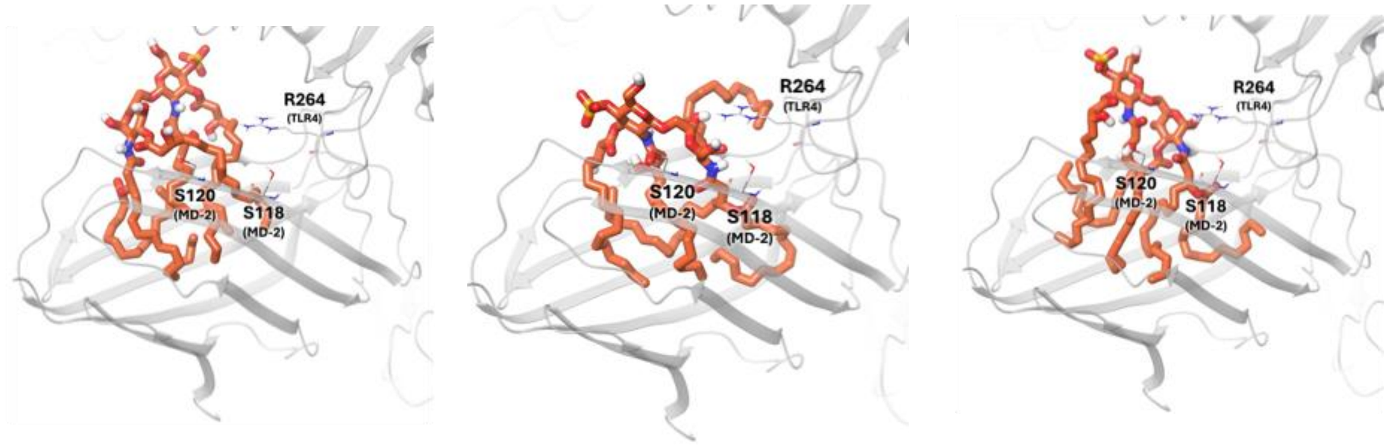

Figure S6. Representative binding modes of **Penta-C4′** in the agonist MD-2/TLR4 model. Volume-overlap clustering identified three families of docking poses, shown in panels (a–c). **(a)** Representative binding mode of cluster 1. The disaccharide displays poor alignment with the crystallographic reference of lipid A (RMSD = 8.82 ± 0.37 Å), and all acyl chains are fully buried within the MD-2 hydrophobic pocket. No key interactions with R264 or other stabilizing residues are observed. **(b)** Representative binding mode of Cluster 2. The sugar moiety is not aligned to lipid A (RMSD = 6.48 ± 0.25 Å), and one acyl chain protrudes from the pocket in an orientation incompatible with receptor dimerization. No key interactions with R264 or other stabilizing residues are observed. **(c)** Representative binding mode of Cluster 3. All the acyl chains are enclosed and the phosphate group is solvent-exposed, preventing the formation of the key interaction with residue R264. The sugar alignment is again inconsistent with the reference ligand binding mode (RMSD = 8.55 ± 0.74 Å) No key interactions with R264 or other stabilizing residues are observed.

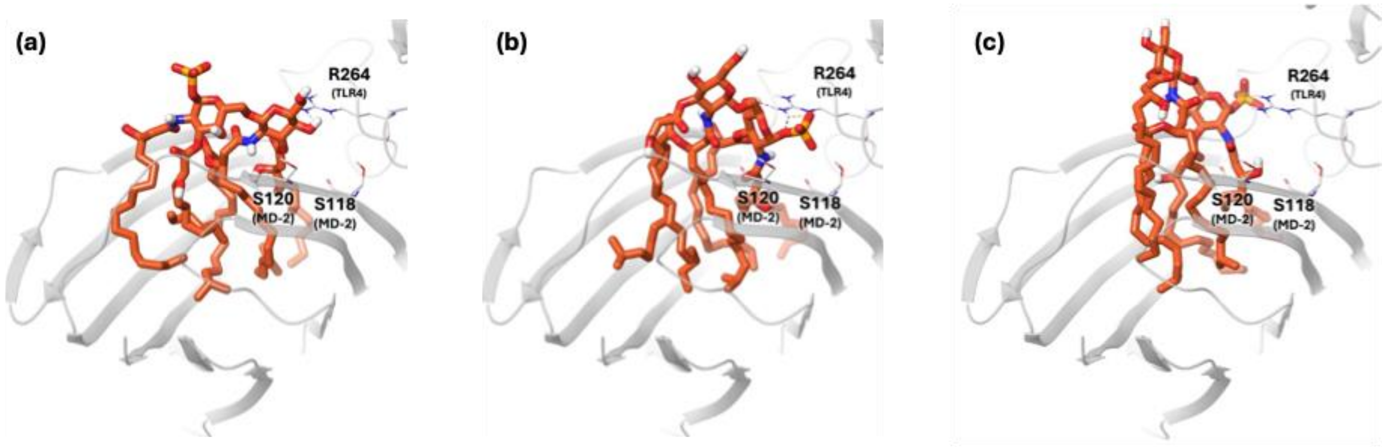

Figure S7. Representative binding modes of **Penta-C1*** in the agonist MD-2/TLR4 model. Volume-overlap clustering identified three families of docking poses, shown in panels (a–c). **(a)** Representative binding mode of cluster 1. The sugar moiety is well aligned with the crystallographic reference (RMSD = 1.05 ± 0.36 Å). The phosphate group is solvent-exposed, precluding the key interaction with R264 and all the acyl chains are buried within the binding pocket, preventing formation of the interface necessary for agonist activity. **(b)** Representative binding mode of cluster 2. Here, the phosphate is correctly oriented towards R264, establishing the aspected salt bridges, but the sugar alignment is poor (RMSD = 5.84 ± 0.06 Å), and all acyl chains are buried, deviating from the expected agonist conformation. **(c)** Representative binding mode of of Cluster 3. The sugar is misaligned (RMSD = 10.37 ± 0.39 Å), no key interactions are maintained, and the acyl chains remain buried, inconsistent with agonist activity.

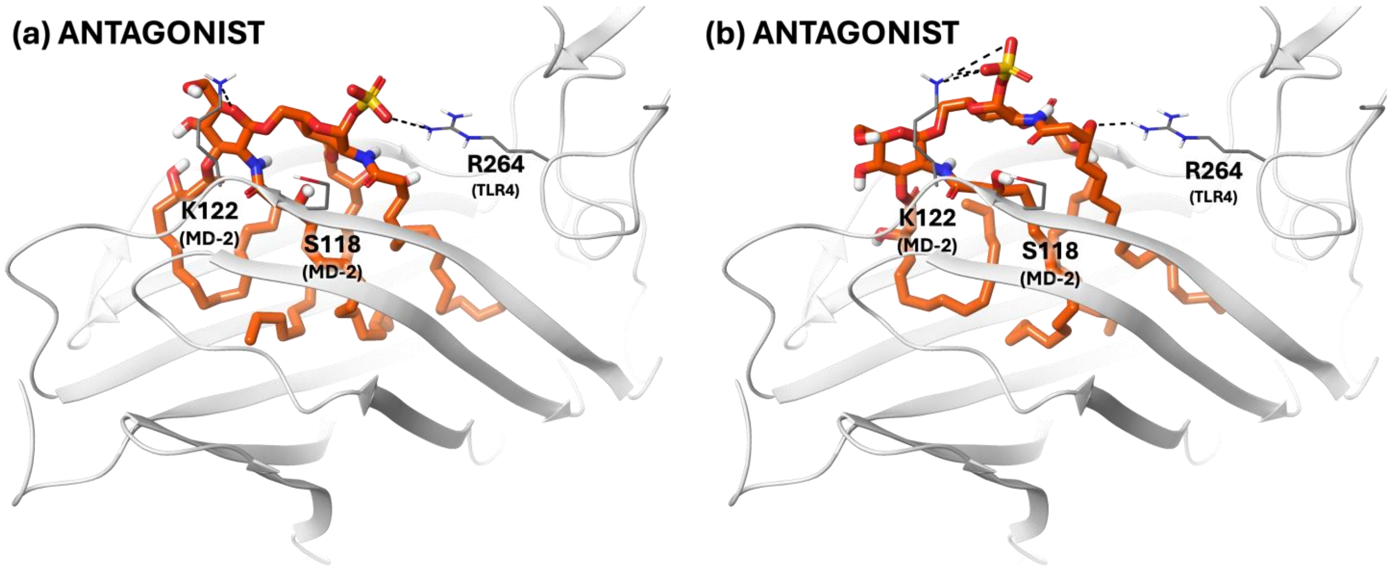

Figure S8. Representative docking poses of **Tetra-C1** (shown in orange) in the antagonist MD-2/TLR4 model. Volume-overlap clustering identified two distinct binding families, shown in panels (a–b).
(a) Representative binding mode of cluster 1. The disaccharide moiety closely aligns with the lipid IVa X-ray reference (average RMSD = 0.56 ± 0.21 Å). All acyl chains are accommodated within the MD-2 pocket, consistent with an antagonist-like conformation. The ligand establishes a salt bridge with residue R264 and a hydrogen bond with K122.
(b) Representative binding mode of cluster 2. In this luster, the disaccharide alignment is less optimal (average RMSD = 3.89 ± 0.74 Å). All acyl chains remain buried within the hydrophobic cavity, but no relevant stabilizing interactions are detected, as the observed hydrogen bond is not phosphate-mediated.

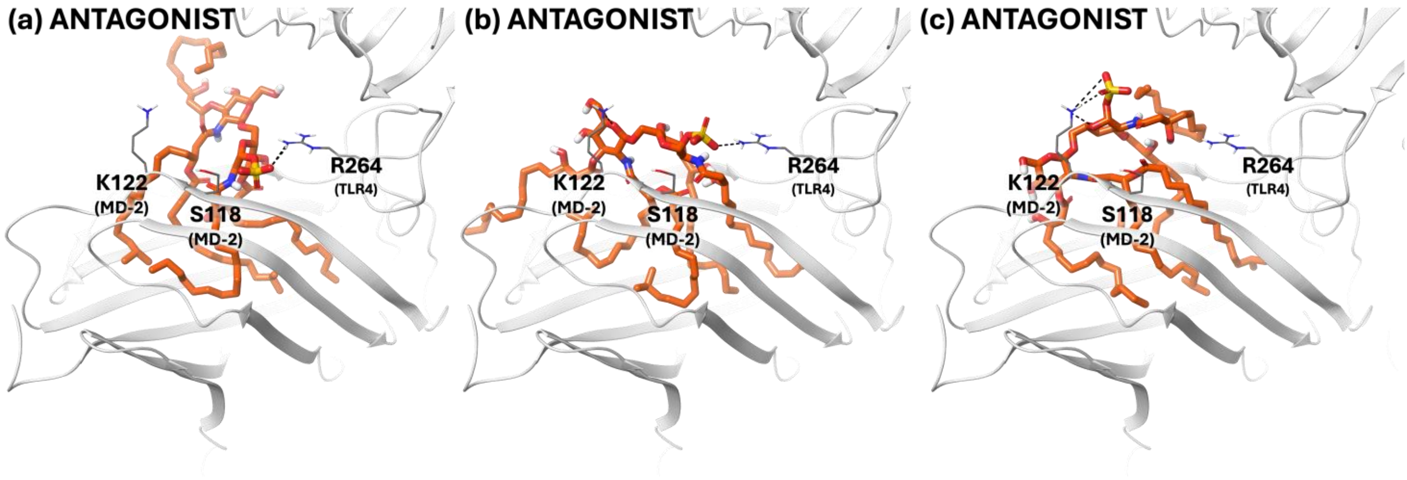

Figure S9. Representative docking poses of **Penta-C1*** (shown in orange) in the antagonist MD-2/TLR4 model. Volume-overlap clustering identified three distinct binding families, shown in panels (a–c).
(a) Representative binding mode of cluster 1. The disaccharide moiety is poorly aligned with the lipid IVa X-ray reference (average RMSD = 7.43 ± 0.42 Å). One acyl chain protrudes from the MD-2 pocket, while the ligand establishes a salt bridge with residue R264 and a hydrogen bond with K122.
(b) Representative binding mode of cluster 2. In this cluster, the disaccharide exhibits a good alignment with the reference structure (average RMSD = 2.48 ± 1.49 Å). Despite the improved alignment, one acyl chain remains exposed outside the cavity. A salt bridge with R264 is observed.
(c) Representative binding mode of cluster 3. The disaccharide shows suboptimal alignment (average RMSD = 4.69 ± 0.68 Å). Again, one acyl chain protrudes from the MD-2 pocket. The ligand forms a salt bridge with K122, though this interaction does not contribute to a stable antagonist-like conformation.

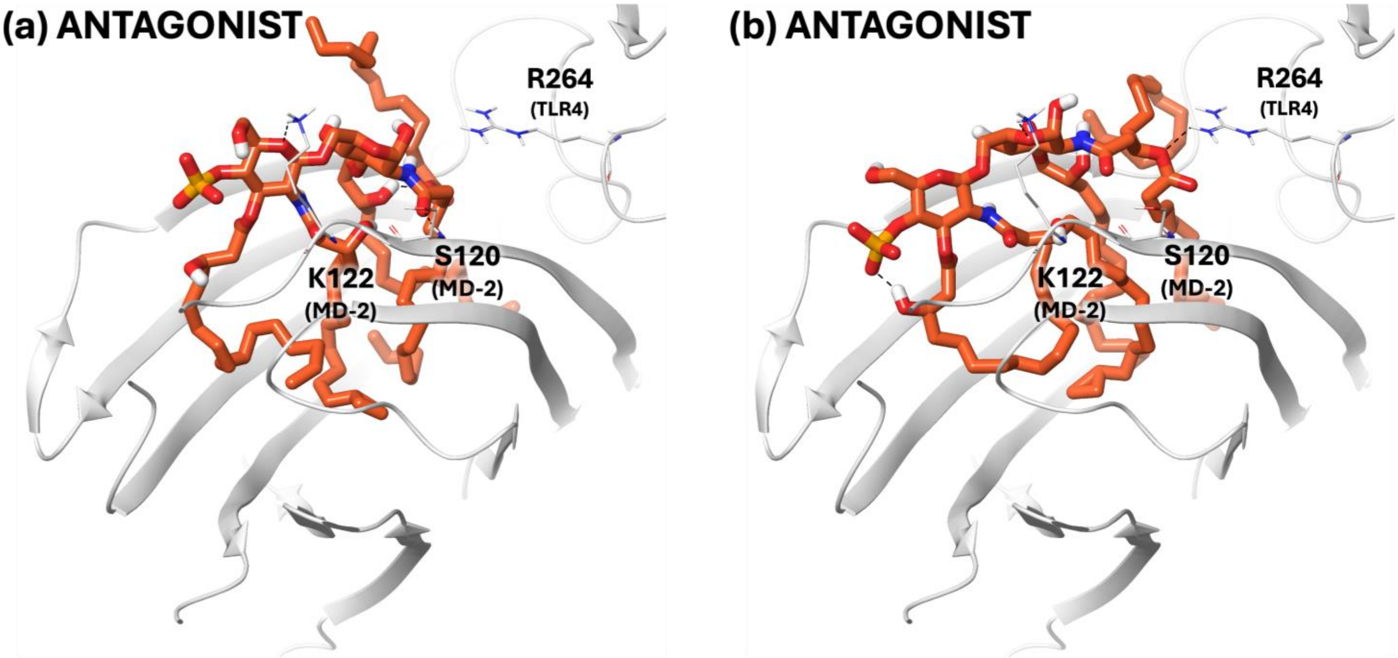

Figure S10. Representative docking poses of **Penta-C4′** (shown in orange) in the antagonist MD-2/TLR4 model. Volume-overlap clustering identified two distinct binding families, shown in panels (a–b).

(a) Representative binding mode of cluster 1. The disaccharide moiety is poorly aligned with the lipid IVa X-ray reference (average RMSD = 4.02 ± 0.63 Å). One acyl chain protrudes from the MD-2 cavity, and no relevant stabilizing interactions are detected.

(b) Representative binding mode of cluster 2. In this cluster, the disaccharide exhibits a good alignment with the reference (average RMSD = 0.64 ± 0.26 Å). However, the phosphate group remains solvent-exposed, preventing the formation of the key salt bridge with residue R264. One acyl chain also protrudes from the pocket, further deviating from a canonical antagonist-like binding mode.

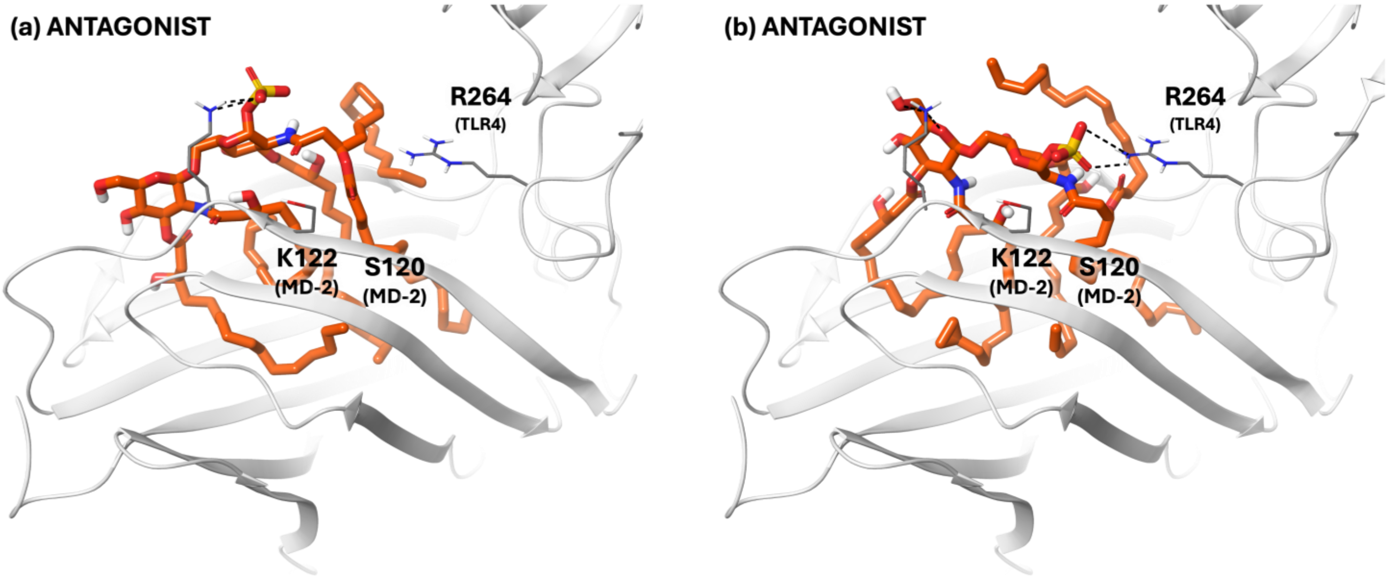

Figure S11. Representative docking poses of **Penta-C1** (shown in orange) in the antagonist MD-2/TLR4 model. Volume-overlap clustering identified two distinct binding families, shown in panels (a–b).

(a) Representative binding mode of cluster 1. The disaccharide moiety is poorly aligned with the lipid IVa X-ray reference (average RMSD = 4.09 ± 0.40 Å). One acyl chain protrudes from the MD-2 cavity, and the phosphate establishes a contact with residue K122, though this interaction does not contribute to a stable antagonist-like conformation.

(b) Representative binding mode of cluster 2. In this cluster, the disaccharide aligns well with the reference (average RMSD = 0.57 ± 0.12 Å). The phosphate group, however, remains solvent-exposed. Importantly, even though the interaction with R264 is conserved, one acyl chain protrudes from the pocket, suggesting that this compound does not adopt a stable antagonist-like conformation.

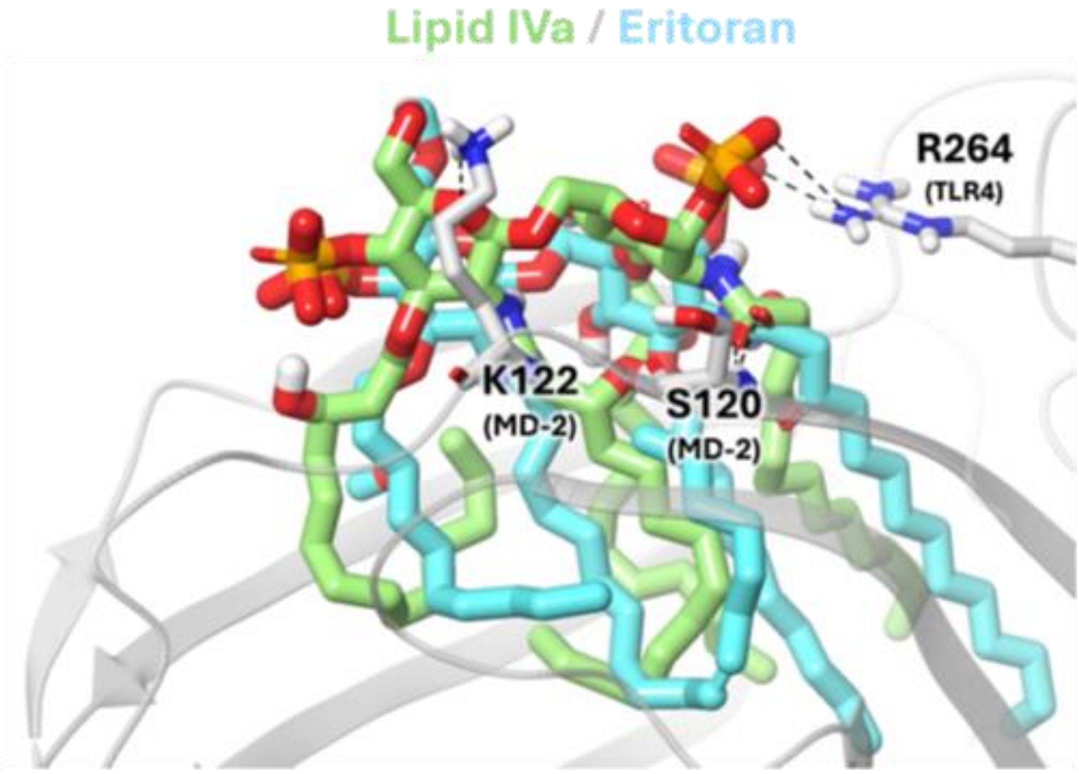

Figure S12. Alignment of the X-ray structures of the hMD-2 binding site (grey) bound to the antagonist Eritoran (blue, PDB ID: 2Z65) and the partial agonist Lipid IVa (green, PDB ID: 2E59). The RMSD of the heavy atoms of the disaccharide core is 3.25 Å.

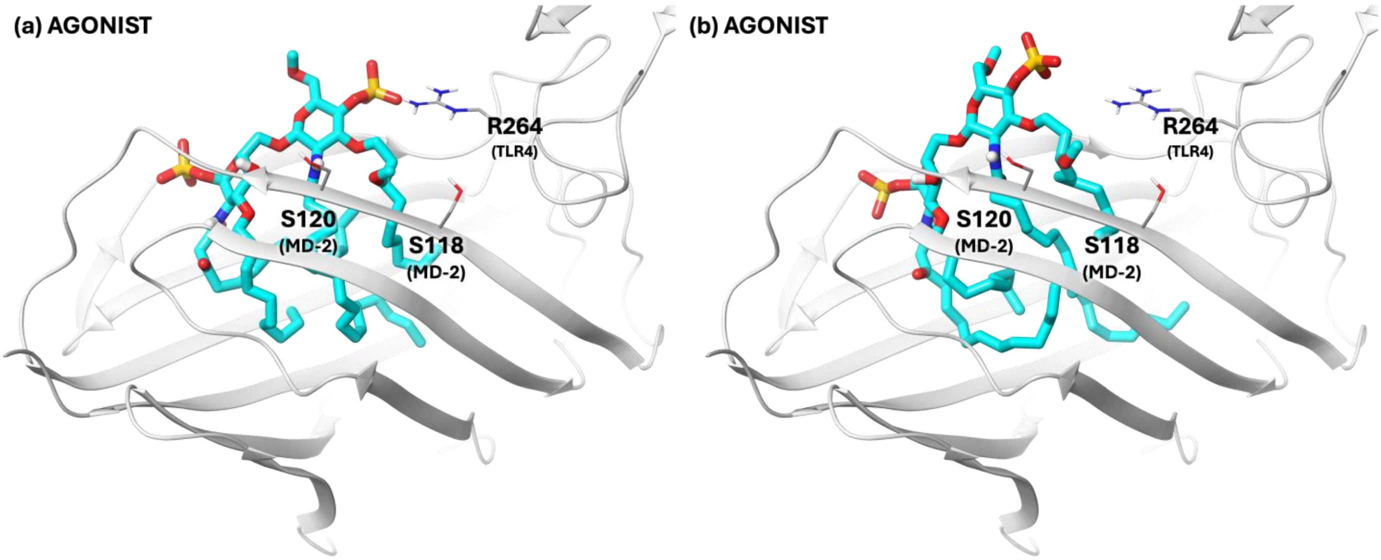

Figure S13. Representative docking poses of **Eritoran** (shown in orange) in the agonist MD-2/TLR4 model. Volume-overlap clustering identified two distinct binding families, shown in panels (a–b).

(a) Representative binding mode of cluster 1. The disaccharide moiety is poorly aligned with the lipid A X-ray reference (average RMSD = 8.58 ± 0.62 Å). All acyl chains are buried within the MD-2 pocket, and no stabilizing interactions are observed, indicating an incompatible agonist-like conformation.

(b) Representative binding mode of cluster 2. Similarly, the disaccharide alignment is poor (average RMSD = 7.84 ± 0.53 Å), with all acyl chains fully buried and no relevant interactions detected, further confirming the incompatibility with an agonist-like binding mode.

	Pose	RMSD (Å)	Autodock score (kcal/mol)
**Cluster 1**	1	8,08	-7,018
	3	9,36	-6,994
	4	8,8	-6,978
	5	10,22	-6,946
	6	8,55	-6,927
	22	8,71	-6,906
	23	8,89	-6,895
	25	8,47	-6,828
	41	9,07	-6,809
	27	7,98	-6,806
	29	8,65	-6,764
	30	9,23	-6,762
	7	7,5	-6,759
	42	8,79	-6,750
	9	8,94	-6,743
	10	8,2	-6,719
	32	9,7	-6,698
	12	9,07	-6,677
	44	7,81	-6,669
	13	8,88	-6,668
	45	8,01	-6,654
	46	8,97	-6,629
	34	8,51	-6,623
	47	9,62	-6,623
	16	9,37	-6,508
	53	8,79	-6,479
	19	10,04	-6,466
	**Average**	**8,82**	**-6,75**
	**Standard dev.**	**0,66**	**0,15**
**Cluster2**	21	5,96	-7,216
	24	6,43	-6,884
	26	6,2	-6,811
	28	6,32	-6,791
	8	6,25	-6,749
	31	5,92	-6,718
	11	5,84	-6,700
	33	6,26	-6,675
	14	6,16	-6,648
	35	6,24	-6,611

	48	6,48	-6,573
	49	6,03	-6,560
	17	6,16	-6,502
	37	6,17	-6,498
	18	6,27	-6,491
	51	6,33	-6,485
	38	6,39	-6,443
	20	6,14	-6,416
	39	6,55	-6,405
	56	6,35	-6,357
	57	6,24	-6,351
	58	6,3	-6,342
	60	5,9	-6,205
	**Average**	**6,21**	**-6,58**
	**Standard dev.**	**0,19**	**0,22**

Table S1. Analysis for compound **Penta-C1** in the agonist form**.** Summary of docking poses after clustering. For each cluster, individual poses are listed with the corresponding pose number, AutoDock score (kcal/mol) and RMSD (Å) calculated with respect to the heavy atoms (14 atoms total, Figure S3) of the disaccharide. Mean and standard deviation values for each metric are reported in **bold** at the end of each cluster section. Poses from different docking replicates are color-coded as follows: green for replicate 1, orange for replicate 2, and blue for replicate 3.

	Pose	RMSD (Å)	Autodock score (kcal/mol)
**Cluster 1**	22	8,68	-7,498
	1	9,08	-7,426
	2	10,66	-7,370
	24	8,93	-7,317
	25	8,48	-7,290
	3	9,46	-7,245
	5	10,32	-7,114
	6	8,61	-7,091
	35	8,73	-7,088
	43	8,69	-6,975
	8	7,73	-6,885
	45	8,72	-6,880
	9	8,76	-6,869
	46	8,92	-6,783
	11	7,98	-6,728
	12	8,47	-6,683
	15	8,22	-6,631
	31	8,66	7,187
	**Average**	**8,84**	**-6,26**
	**Standard dev.**	**0,72**	**3,37**
**Cluster 2**	21	11,00	-7,59
	26	11,99	-7,28
	28	11,92	-7,24
	29	10,25	-7,23
	30	11,02	-7,22
	32	10,23	-7,18
	34	11,81	-7,15
	36	10,89	-7,07
	10	11,10	-6,79
	40	11,16	-6,73
	50	10,63	-6,70
	17	11,64	-6,49
	19	11,61	-6,35
	20	10,19	-6,30
	**Average**	**11,10**	**-6,95**
	**Standard dev.**	**0,63**	**0,39**

Table S2. Analysis for compound **Tetra-C1** in the agonist form. Summary of docking poses after clustering. For each cluster, individual poses are listed with the corresponding pose number, AutoDock score (kcal/mol) and RMSD (Å) calculated with respect to the heavy atoms (14 atoms total, Figure S3) of the disaccharide. Mean and standard

deviation values for each metric are reported in **bold** at the end of each cluster section. Poses from different docking replicates are color-coded as follows: green for replicate 1, orange for replicate 2, and blue for replicate 3.

	Pose	RMSD (Å)	Autodock score (kcal/mol)
**Cluster 1**	23	8,93	-7,190
	1	8,8	-6,650
	43	8,93	-6,554
	2	9,2	-6,493
	4	7,88	-6,235
	5	8,99	-6,228
	8	8,91	-6,114
	13	8,82	-6,017
	15	8,9	-5,946
	**Average**	8,82	-6,38
	**Standard dev.**	0,37	0,39
**Cluster 2**	21	6,61	-7,250
	41	6,61	-7,250
	22	6,48	-7,217
	42	6,48	-7,217
	25	6,35	-7,120
	45	6,35	-7,120
	28	6,14	-7,068
	48	6,14	-7,068
	30	6,74	-7,058
	50	6,74	-7,058
	32	6,11	-6,794
	38	6,88	-6,794
	52	6,11	-6,794
	58	6,88	-6,794
	3	6,35	-6,245
	6	6,49	-6,144
	7	6,79	-6,129
	9	6,63	-6,112
	10	6,65	-6,057
	14	6,19	-5,967
	16	6,43	-5,932
	**Average**	6,48	-6,72
	**Standard dev.**	0,25	0,49
**Cluster 3**	29	8,68	-7,063
	49	8,68	-7,063
	33	8,49	-6,941
	53	8,49	-6,941
	34	7,59	-6,912

	54	7,59	-6,912
	35	9,35	-6,873
	55	9,35	-6,873
	36	9	-6,870
	56	9	-6,870
	37	7,49	-6,863
	57	7,49	-6,863
	39	8,78	-6,788
	59	8,78	-6,788
	40	9,81	-6,688
	60	9,81	-6,688
	11	7,66	-6,041
	12	9,29	-6,040
	17	8,48	-5,921
	18	8,62	-5,877
	19	7,77	-5,842
	20	7,98	-5,754
	**Average**	8,55	-6,61
	**Standard dev.**	0,74	0,45

Table S3. Analysis for compound **Penta-C4'** in the agonist form**.** Summary of docking poses after clustering. For each cluster, individual poses are listed with the corresponding pose number, AutoDock score (kcal/mol) and RMSD (Å) calculated with respect to the heavy atoms (14 atoms total, Figure S3) of the disaccharide. Mean and standard deviation values for each metric are reported in **bold** at the end of each cluster section. Poses from different docking replicates are color-coded as follows: green for replicate 1, orange for replicate 2, and blue for replicate 3.

	Pose	RMSD (Å)	Autodock score (kcal/mol)
**Cluster 1**	2	1,22	-7,206
	5	1,27	-7,135
	6	1,08	-7,119
	9	0,87	-7,052
	11	1,53	-6,995
	24	0,69	-6,940
	13	0,93	-6,924
	25	0,98	-6,922
	15	0,89	-6,888
	17	1,65	-6,805
	18	0,67	-6,797
	29	0,85	-6,782
	30	1,74	-6,763
	31	1,12	-6,734
	42	1,40	-6,723
	20	1,39	-6,668
	37	0,77	-6,654
	38	0,87	-6,591
	39	0,88	-6,483
	51	0,50	-6,289
	54	0,72	-6,228
	55	0,51	-6,226
	57	1,60	-6,174
	**Average**	**1,05**	**-6,743**
	**Standard dev.**	**0,36**	**0,299**
**Cluster 2**	3	5,78	-7,178
	7	5,79	-7,090
	8	5,83	-7,079
	26	5,83	-6,913
	27	5,97	-6,865
	46	5,83	-6,374
	59	5,86	-6,114
	**Average**	**5,84**	**-6,802**
	**Standard dev.**	**0,06**	**0,403**
**Cluster 3**	10	10,45	-7,051
	12	10,59	-6,987
	14	10,58	-6,901
	16	10,18	-6,866
	35	11,20	-6,694

	36	10,09	-6,689
	43	10,26	-6,481
	40	10,07	-6,465
	44	10,81	-6,446
	45	10,71	-6,404
	49	9,99	-6,296
	52	9,79	-6,272
	56	10,13	-6,222
	**Average**	**10,37**	**-6,598**
	**Standard dev.**	**0,39**	**0,284**

Table S4. Analysis for compound **Penta-C1*** in the agonist form**.** Summary of docking poses after clustering. For each cluster, individual poses are listed with the corresponding pose number, AutoDock score (kcal/mol) and RMSD (Å) calculated with respect to the heavy atoms (14 atoms total, Figure S3) of the disaccharide. Mean and standard deviation values for each metric are reported in **bold** at the end of each cluster section. Poses from different docking replicates are color-coded as follows: green for replicate 1, orange for replicate 2, and blue for replicate 3.

	Pose	MM-GBSA (kcal/mol)	RMSD (Å)	Autodock score (kcal/mol)
**Cluster 1**	1	-129,79	0,52	-7,645
	41	-125,75	0,25	-7,532
	42	-132,72	0,32	-7,521
	21	-106,82	0,34	-7,423
	22	-124,01	1,05	-7,405
	43	-126,63	0,51	-7,348
	23	-122,44	0,62	-7,338
	2	-118,17	0,85	-7,337
	3	-99,63	0,87	-7,307
	24	-118,04	0,27	-7,283
	4	-119,48	0,49	-7,172
	5	-125,49	0,69	-7,167
	6	-115,59	0,29	-7,126
	7	-113,51	0,36	-7,083
	8	-120,76	0,50	-7,073
	25	-127,47	0,64	-7,056
	26	-125,68	0,43	-7,049
	28	-119,31	0,50	-7,029
	44	-107,28	0,59	-6,995
	45	-131,97	0,81	-6,983
	9	-100,64	0,30	-6,971
	11	-109,29	0,51	-6,896
	12	-122,50	0,71	-6,871
	48	-107,24	0,59	-6,862
	29	-114,42	0,87	-6,793
	30	-74,74	0,54	-6,775
	13	-145,48	0,55	-6,714
	50	-100,37	0,80	-6,657
	51	-96,21	0,37	-6,622
	32	-104,98	0,75	-6,548
	**Average**	**-116,10**	**0,56**	**-7,086**
	**Standard dev.**	**14,09**	**0,21**	**0,287**
**Cluster 2**	10	-135,51	3,13	-6,905
	49	-116,68	4,16	-6,857
	15	-127,58	3,38	-6,613
	52	-129,03	4,11	-6,608
	31	-126,46	4,59	-6,603
	16	-134,24	3,57	-6,576
	17	-100,35	4,96	-6,524

	33	-124,64	3,76	-6,490
	35	-138,93	2,58	-6,424
	36	-117,01	3,35	-6,359
	37	-118,07	3,84	-6,275
	38	-100,69	3,87	-6,249
	20	-115,26	5,25	-6,113
	**Average**	**-121,88**	**3,89**	**-6,507**
	**Standard dev.**	**12,12**	**0,74**	**0,228**

Table S5. Analysis for compound **Tetra-C1** in the antagonist form**.** Summary of docking poses after clustering. For each cluster, individual poses are listed with the corresponding pose number, AutoDock score (kcal/mol), MM-GBSA binding free energy (kcal/mol), and RMSD (Å) calculated with respect to the heavy atoms (14 atoms total, Figure S3) of the disaccharide. Mean and standard deviation values for each metric are reported in **bold** at the end of each cluster section. Poses from different docking replicates are color-coded as follows: green for replicate 1, orange for replicate 2, and blue for replicate 3.

	Pose	MM-GBSA (kcal/mol)	RMSD (Å)	Autodock score (kcal/mol)
**Cluster 1**	44	-121,10	7,39	-6,672
	49	-126,74	7,62	-6,496
	23	-132,88	7,87	-6,471
	57	-98,90	7,70	-6,349
	8	-121,46	7,72	-6,239
	9	-81,31	7,24	-6,221
	10	-121,63	7,36	-6,200
	11	-126,97	6,54	-6,195
	**Average**	**-116,37**	**7,43**	**-6,355**
	**Standard dev.**	**17,33**	**0,42**	**0,175**
**Cluster 2**	41	-141,95	0,53	-6,892
	42	-128,81	0,58	-6,822
	43	-122,83	2,24	-6,809
	1	-117,02	1,24	-6,702
	2	-107,33	0,60	-6,601
	3	-131,23	1,76	-6,595
	4	-125,67	2,55	-6,403
	6	-112,70	3,37	-6,284
	60	-113,93	3,25	-6,186
	36	-109,76	3,99	-6,121
	13	-109,69	5,02	-6,102
	14	-86,42	2,70	-6,099
	18	-115,14	4,35	-5,945
	**Average**	**-117,11**	**2,48**	**-6,428**
	**Standard dev.**	**13,66**	**1,49**	**0,325**
**Cluster 3**	22	-120,33	3,59	-6,542
	50	-120,55	4,99	-6,486
	51	-105,86	5,55	-6,484
	55	-110,75	3,93	-6,402
	56	-88,76	5,33	-6,397
	24	-74,59	4,79	-6,366
	59	-85,76	5,13	-6,299
	27	-142,91	3,44	-6,276
	37	-70,25	4,75	-6,012
	38	-120,45	4,83	-5,899
	19	-97,42	4,71	-5,841
	39	-77,83	5,19	-5,799
	**Average**	**-101,29**	**4,69**	**-6,234**
	**Standard dev.**	**22,53**	**0,68**	**0,270**

Table S6. Analysis for compound **Penta-C1*** in the antagonist form**.** Summary of docking poses after clustering. For each cluster, individual poses are listed with the corresponding pose number, AutoDock score (kcal/mol), MM-GBSA binding free energy (kcal/mol), and RMSD (Å) calculated with respect to the heavy atoms (14 atoms total, Figure S3) of the disaccharide. Mean and standard deviation values for each metric are reported in **bold** at the end of each cluster section. Poses from different docking replicates are color-coded as follows: green for replicate 1, orange for replicate 2, and blue for replicate 3.

	Pose	MM-GBSA (kcal/mol)	RMSD (Å)	Autodock score (kcal/mol)
**Cluster 1**	2	-112,77	3,87	-6,959
	3	-87,98	3,67	-6,853
	5	-106,26	5,97	-6,789
	23	-100,43	4,04	-6,777
	7	-104,82	3,92	-6,726
	24	-80,37	4,03	-6,703
	25	-126,22	3,35	-6,663
	27	-122,19	3,29	-6,610
	14	-87,86	4,31	-6,604
	41	-110,23	3,58	-6,598
	15	-94,88	4,72	-6,581
	28	-113,97	3,87	-6,570
	29	-123,92	4,10	-6,557
	42	-105,04	3,84	-6,535
	30	-101,94	2,97	-6,528
	43	-128,78	4,11	-6,504
	44	-112,16	4,48	-6,458
	32	-117,66	4,12	-6,453
	45	-127,03	5,15	-6,453
	18	-94,13	3,89	-6,423
	34	-118,02	4,25	-6,380
	36	-98,40	3,56	-6,361
	46	-118,51	3,75	-6,313
	47	-129,76	3,04	-6,271
	38	-125,20	4,04	-6,198
	49	-119,42	4,58	-6,184
	50	-105,97	4,63	-6,177
	51	-72,15	4,32	-6,108
	52	-120,15	3,04	-6,096
	53	-108,13	5,10	-6,085
	54	-121,95	3,83	-6,079
	55	-113,10	3,44	-5,966
	56	-138,52	4,28	-5,951
	57	-130,95	3,50	-5,858
	**Average**	**-111,14**	**4,02**	**-6,423**
	**Standard dev.**	**15,36**	**0,63**	**0,278**
**Cluster 2**	1	-119,25	0,54	-7,082
	21	-138,96	0,98	-7,077
	22	-96,78	0,63	-6,851

	4	-92,30	0,29	-6,841
	6	-111,72	0,90	-6,773
	10	-130,65	0,79	-6,637
	11	-126,94	0,38	-6,613
	**Average**	**-116,66**	**0,64**	**-6,839**
	**Standard dev.**	**17,41**	**0,26**	**0,188**

Table S7. Analysis for compound **Penta-C4'** in the antagonist form. Summary of docking poses after clustering. For each cluster, individual poses are listed with the corresponding pose number, AutoDock score (kcal/mol), MM-GBSA binding free energy (kcal/mol), and RMSD (Å) calculated with respect to the heavy atoms (14 atoms total, Figure S3) of the disaccharide. Mean and standard deviation values for each metric are reported in bold at the end of each cluster section. Poses from different docking replicates are color-coded as follows: green for replicate 1, orange for replicate 2, and blue for replicate 3.

	Pose	MM-GBSA (kcal/mol)	RMSD (Å)	Autodock score (kcal/mol)
**Cluster 1**	44	-126	4,52	-6,747
	46	-110,99	5	-6,686
	25	-148,96	3,65	-6,671
	26	-117,38	4,24	-6,669
	27	-127,05	3,95	-6,669
	49	-131,23	4,2	-6,643
	30	-116,32	3,65	-6,637
	50	-121,97	4,46	-6,583
	52	-138,47	4,05	-6,54
	33	-116,85	4	-6,511
	54	-107,17	3,34	-6,508
	34	-116,97	4,4	-6,478
	56	-117,12	3,61	-6,477
	1	-120,36	4,5	-6,451
	3	-113,41	3,79	-6,425
	4	-122,31	4,21	-6,405
	5	-139,08	4,71	-6,386
	36	-122,86	4,41	-6,372
	58	-130,05	4,45	-6,29
	6	-134,21	4,42	-6,275
	60	-111,22	3,98	-6,238
	38	-110,4	3,69	-6,204
	7	-124,74	3,64	-6,191
	8	-123,28	3,96	-6,132
	11	-119,56	3,8	-6,018
	13	-128,63	4,35	-6,004
	14	-97,88	3,67	-5,985
	17	-118,56	3,79	-5,917
	**Average**	**-121,89**	**4,09**	**-6,40**
	**Standard dev.**	**10,68**	**0,40**	**0,24**
**Cluster 2**	21	-106,6	0,48	-7,177
	22	-110,31	0,34	-7,103
	23	-120,91	0,52	-7,021
	41	-123,87	0,47	-7,01
	42	-117,86	0,51	-6,951
	24	-116,32	0,59	-6,855
	43	-125,28	0,53	-6,853
	45	-119,33	0,56	-6,713
	47	-122,8	0,86	-6,652

	28	-131,91	0,58	-6,641
	29	-126,09	0,67	-6,641
	31	-103,56	0,65	-6,636
	32	-130,99	0,65	-6,571
	55	-110,09	0,63	-6,483
	35	-117,18	0,61	-6,447
	2	-124,44	0,43	-6,446
	**Average**	**-119,22**	**0,57**	**-6,76**
	**Standard dev.**	**8,29**	**0,12**	**0,24**

Table S8. Analysis for compound **Penta-C1** in the antagonist form**.** Summary of docking poses after clustering. For each cluster, individual poses are listed with the corresponding pose number, AutoDock score (kcal/mol), MM-GBSA binding free energy (kcal/mol), and RMSD (Å) calculated with respect to the heavy atoms (14 atoms total, Figure S3) of the disaccharide. Mean and standard deviation values for each metric are reported in **bold** at the end of each cluster section. Poses from different docking replicates are color-coded as follows: green for replicate 1, orange for replicate 2, and blue for replicate 3.

	Pose	RMSD (Å)	Autodock score (kcal/mol)
**Cluster 1**	1	8,29	-7,809
	43	8,24	-7,608
	44	8,29	-7,546
	45	7,94	-7,409
	47	9,31	-7,340
	3	8,83	-7,241
	49	8,18	-7,233
	53	9,16	-7,060
	4	7,32	-7,036
	5	9,25	-6,959
	55	7,59	-6,863
	6	8,56	-6,829
	7	8,20	-6,742
	58	8,01	-6,736
	21	7,76	-6,657
	9	9,12	-6,649
	10	9,18	-6,642
	22	8,19	-6,554
	12	9,27	-6,523
	24	9,11	-6,501
	13	9,07	-6,493
	14	9,03	-6,419
	25	8,54	-6,399
	15	9,00	-6,354
	26	8,36	-6,279
	27	7,29	-6,241
	28	7,80	-6,225
	29	9,16	-6,211
	31	9,09	-5,952
	32	8,56	-5,938
	36	9,17	-5,790
	37	9,16	-5,758
	40	9,21	-5,450
	**Average**	**8,58**	**-6,650**
	**Standard dev.**	**0,62**	**0,566**
**Cluster 2**	48	7,65	-7,244
	50	7,81	-7,071
	51	8,72	-7,070
	52	7,12	-7,065

	54	8,50	-6,939
	57	7,55	-6,830
	11	7,44	-6,591
	60	8,13	-6,589
	23	7,42	-6,535
	16	7,36	-6,347
	17	7,39	-6,340
	18	7,66	-6,230
	20	8,76	-6,020
	33	7,43	-5,916
	34	7,50	-5,907
	35	8,35	-5,854
	39	8,48	-5,716
	**Average**	**7,84**	**-6,486**
	**Standard dev.**	**0,53**	**0,495**

Table S9. Analysis for **Eritoran** in the agonist form. Summary of docking poses after clustering. For each cluster, individual poses are listed with the corresponding pose number, AutoDock score (kcal/mol) and RMSD (Å) calculated with respect to the heavy atoms (14 atoms total, Figure S3) of the disaccharide. Mean and standard deviation values for each metric are reported in bold at the end of each cluster section. Poses from different docking replicates are color-coded as follows: green for replicate 1, orange for replicate 2, and blue for replicate 3.

	Pose	MM-GBSA (kcal/mol)	RMSD (Å)	Autodock score (kcal/mol)
**Cluster 1**	42	-83,74	3,51	-7,692
	45	-76,85	5,64	-7,409
	46	-59,30	4,73	-7,341
	50	-80,09	3,00	-7,071
	1	-70,52	3,96	-6,620
	23	-75,70	5,03	-6,535
	24	-70,39	5,83	-6,501
	25	-84,75	3,14	-6,399
	4	-73,16	5,93	-6,134
	5	-81,40	4,16	-6,062
	6	-53,29	4,58	-5,988
	11	-92,73	3,78	-5,914
	36	-76,00	4,16	-5,790
	40	-96,73	3,40	-5,450
	**Average**	**-76,76**	**4,35**	**-6,493**
	**Standard dev.**	**11,60**	**0,98**	**0,670**
**Cluster 2**	52	-97,84	5,29	-7,065
	27	-78,40	5,27	-6,241
	28	-78,29	5,42	-6,225
	32	-65,43	5,54	-5,938
	10	-64,32	5,30	-5,932
	35	-52,90	5,51	-5,854
	13	-65,00	5,31	-5,831
	15	-67,93	5,22	-5,814
	38	-86,16	5,39	-5,750
	39	-62,89	5,36	-5,716
	19	-63,69	5,42	-5,679
	**Average**	**-71,17**	**5,37**	**-6,004**
	**Standard dev.**	**12,75**	**0,10**	**0,398**
**Cluster 3**	41	-86,09	5,12	-7,721
	43	-80,93	5,19	-7,608
	44	-66,94	5,16	-7,546
	47	-68,91	5,11	-7,340
	48	-77,61	5,00	-7,244
	53	-72,59	4,95	-7,060
	54	-55,05	5,06	-6,939
	58	-68,61	4,97	-6,736
	59	-55,66	4,98	-6,627
	60	-54,31	4,97	-6,589

	26	-88,22	5,06	-6,279
	30	-60,80	5,01	-6,144
	7	-74,26	5,10	-5,982
	8	-79,01	5,11	-5,977
	33	-79,01	5,13	-5,916
	37	-78,72	4,98	-5,758
	18	-69,21	4,95	-5,712
	20	-69,46	5,12	-5,626
	**Average**	**-71,41**	**5,05**	**-6,600**
	**Standard dev.**	**10,20**	**0,08**	**0,716**

Table S10. Analysis for **Eritoran** in the antagonist form**.** Summary of docking poses after clustering. For each cluster, individual poses are listed with the corresponding pose number, AutoDock score (kcal/mol), MM-GBSA binding free energy (kcal/mol), and RMSD (Å) calculated with respect to the heavy atoms (14 atoms total, Figure S3) of the disaccharide. Mean and standard deviation values for each metric are reported in **bold** at the end of each cluster section. Poses from different docking replicates are color-coded as follows: green for replicate 1, orange for replicate 2, and blue for replicate 3.

	Pose	RMSD (Å)	Autodock score (kcal/mol)
**Cluster 1**	41	0,66	-7,697
	42	0,89	-7,514
	43	0,41	-7,461
	44	0,32	-7,415
	45	0,44	-7,412
	46	0,56	-7,381
	47	0,54	-7,370
	21	0,42	-7,353
	48	0,35	-7,291
	22	0,56	-7,149
	49	0,45	-7,146
	23	0,81	-7,091
	24	0,54	-7,085
	50	0,67	-7,085
	51	0,29	-7,078
	52	0,32	-7,022
	1	0,35	-6,856
	2	0,74	-6,839
	59	0,57	-6,760
	4	0,29	-6,631
	6	0,56	-6,612
	7	0,56	-6,611
	8	0,59	-6,554
	9	0,25	-6,527
	10	0,44	-6,461
	14	0,61	-6,283
	31	0,52	-6,142
	33	0,61	-6,118
	20	0,64	-5,906
	**Average**	**0,52**	**-6,926**
	**Standard dev.**	**0,16**	**0,470**

Table S11. Analysis for **Lipid A** in the agonist form. Summary of docking poses reproducing the X-ray conformation. For each selected pose, the pose number, AutoDock score (kcal/mol) and RMSD (Å) with respect to the heavy atoms (14 atoms total, Figure S3) of the disaccharide in the X-ray structure are reported. Mean and standard deviation values for each metric are shown in bold at the end of the list. Poses from different docking replicates are color-coded as follows: green for replicate 1, orange for replicate 2, and blue for replicate 3.

	Pose	MM-GBSA (kcal/mol)	RMSD (Å)	Autodock score (kcal/mol)
**Cluster 1**	1	-126,09	0,80	-7,839
	2	-128,31	1,58	-7,745
	3	-134,49	0,87	-7,727
	4	-103,42	0,93	-7,657
	5	-110,41	0,74	-7,581
	6	-104,62	0,96	-7,422
	8	-122,49	1,38	-7,250
	41	-108,65	1,02	-7,228
	9	-105,45	0,69	-7,178
	42	-107,38	0,73	-7,055
	12	-113,63	0,95	-6,997
	21	-114,30	1,00	-6,975
	14	-90,79	0,65	-6,963
	43	-115,97	0,96	-6,949
	44	-128,31	1,36	-6,934
	45	-104,93	1,12	-6,933
	46	-124,46	1,00	-6,843
	47	-119,45	1,45	-6,839
	48	-105,00	0,39	-6,825
	22	-124,70	1,64	-6,707
	50	-119,15	1,03	-6,691
	23	-123,96	0,73	-6,687
	52	-99,65	0,81	-6,613
	24	-129,48	0,75	-6,610
	55	-79,00	0,77	-6,397
	25	-118,59	0,93	-6,385
	26	-90,64	0,71	-6,338
	58	79,00	1,02	-6,334
	32	-104,77	0,98	-6,265
	**Average**	**-105,98**	**0,96**	**-6,989**
	**Standard dev.**	**40,04**	**0,29**	**0,438**

Table S12. Analysis for **Lipid Va** in the antagonist form**.** Summary of docking poses reproducing the X-ray conformation. For each selected pose, the pose number, AutoDock score (kcal/mol), MM-GBSA binding free energy (kcal/mol), and RMSD (Å) with respect to the heavy atoms (14 atoms total, Figure S3) of the disaccharide in the X-ray structure are reported. Mean and standard deviation values for each metric are shown in bold at the end of the list. Poses from different docking replicates are color-coded as follows: green for replicate 1, orange for replicate 2, and blue for replicate 3.

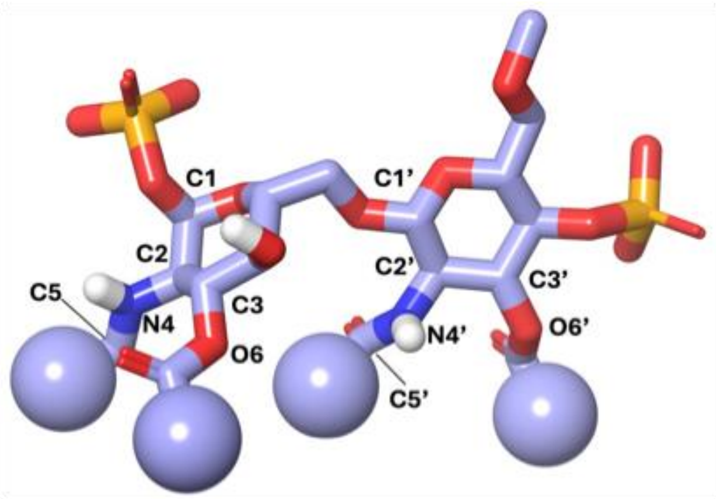

Reference structure	C1-C2-N4-C5	C1-C2-C3-O6	C1'-C2'-N4'-C5'	C1'-C2'-C3'-O6'
**Lipid A**	125.0	-169.1	100.7	163.5
**Lipid IVa**	79.0	-176.5	129.4	-167.9

Table S13. Dihedral values of the lipophilic chains constrained in the semi-flexible docking protocol. Values for the agonist (Lipid A) and antagonist (Lipid IVa) conformations are derived from the corresponding X-ray structures.

Compound 9

^{1}H-NMR (400 MHz, $CDCl_3$)

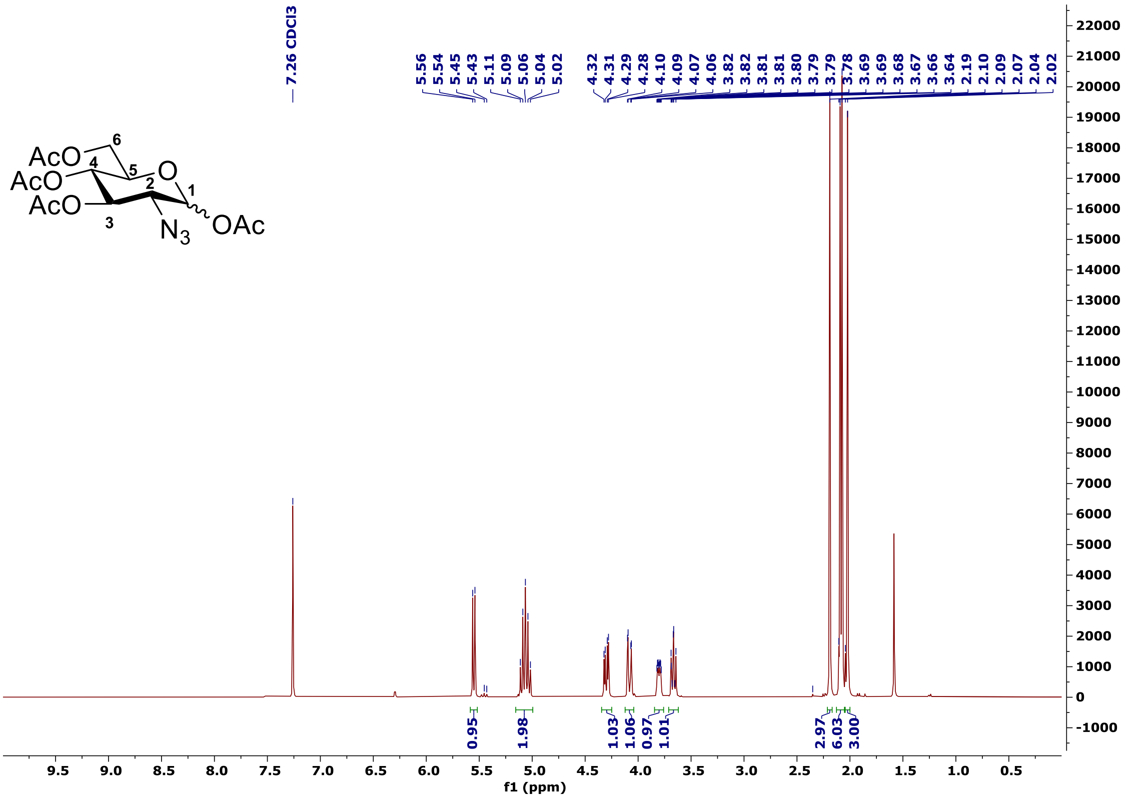

- The spectroscopic data are in agreement with those reported in literature [1].

Compound 10

^{1}H-NMR (400 MHz, $CDCl_3$) on crude material

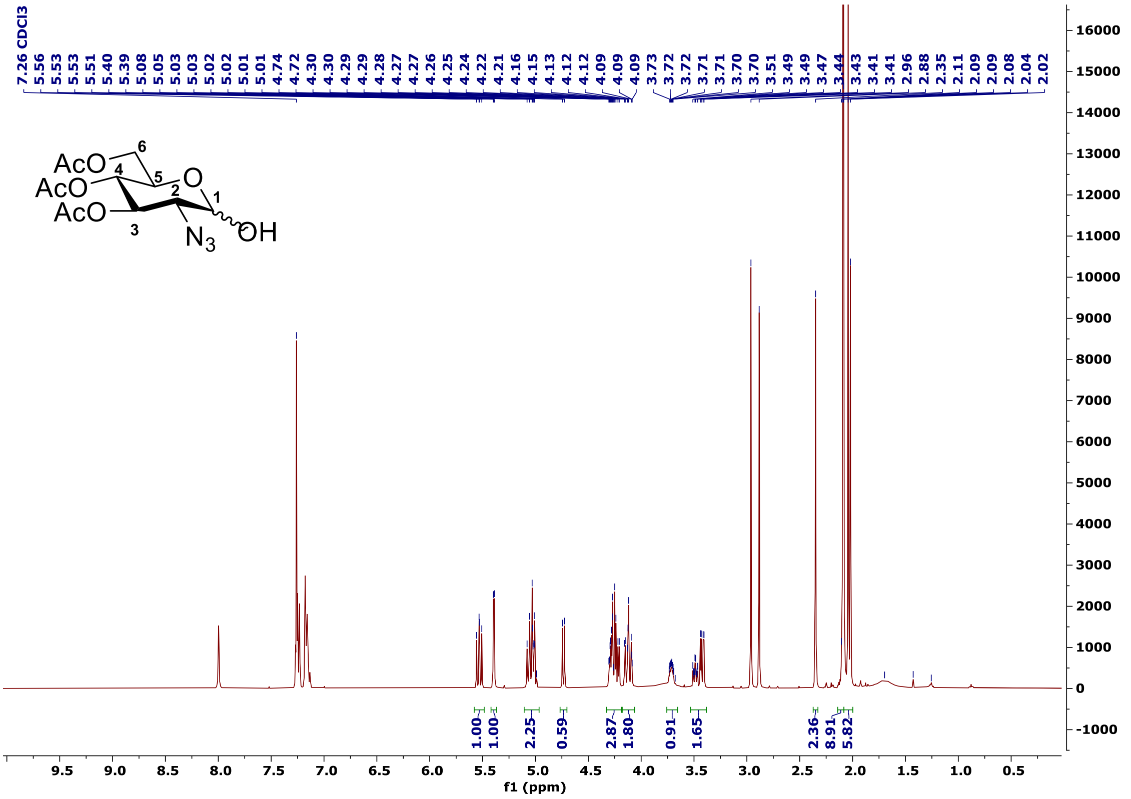

- The spectroscopic data are in agreement with those reported in literature [2].

Compound 11

^{1}H-NMR (400 MHz, $CDCl_3$)

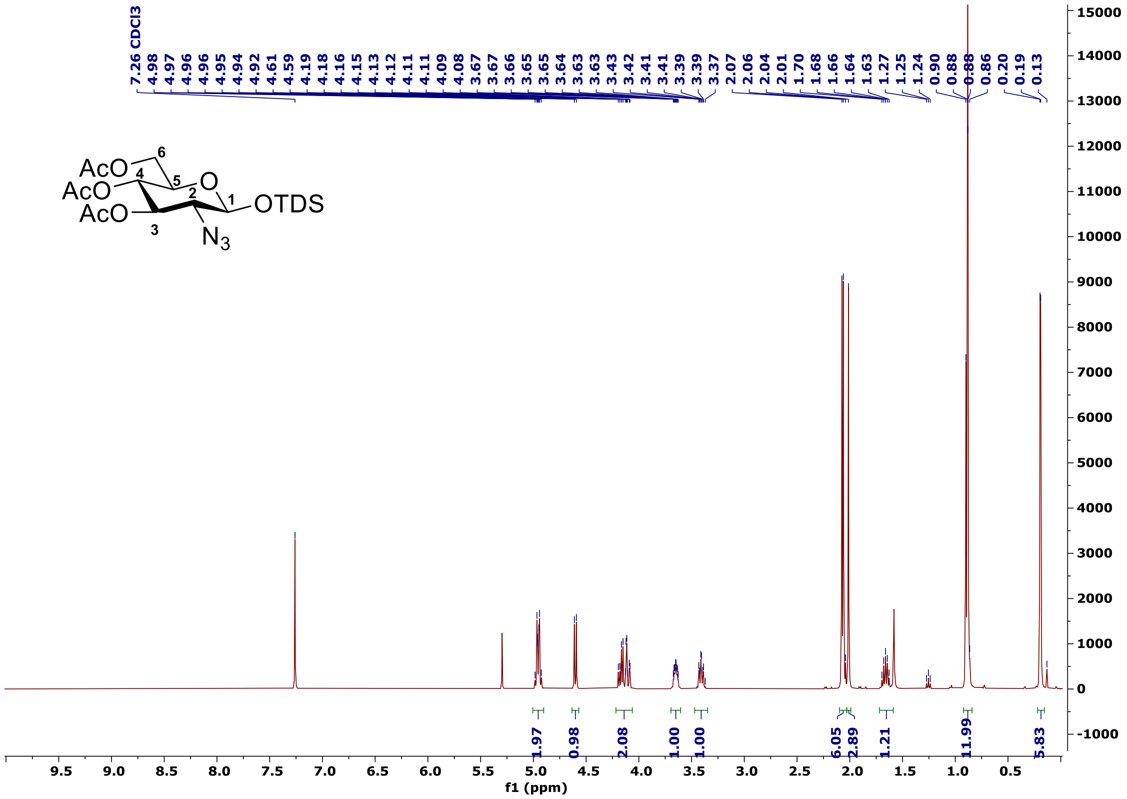

- The spectroscopic data are in agreement with those reported in literature [1].

Compound 12

^{1}H-NMR (400 MHz, $CDCl_3$)

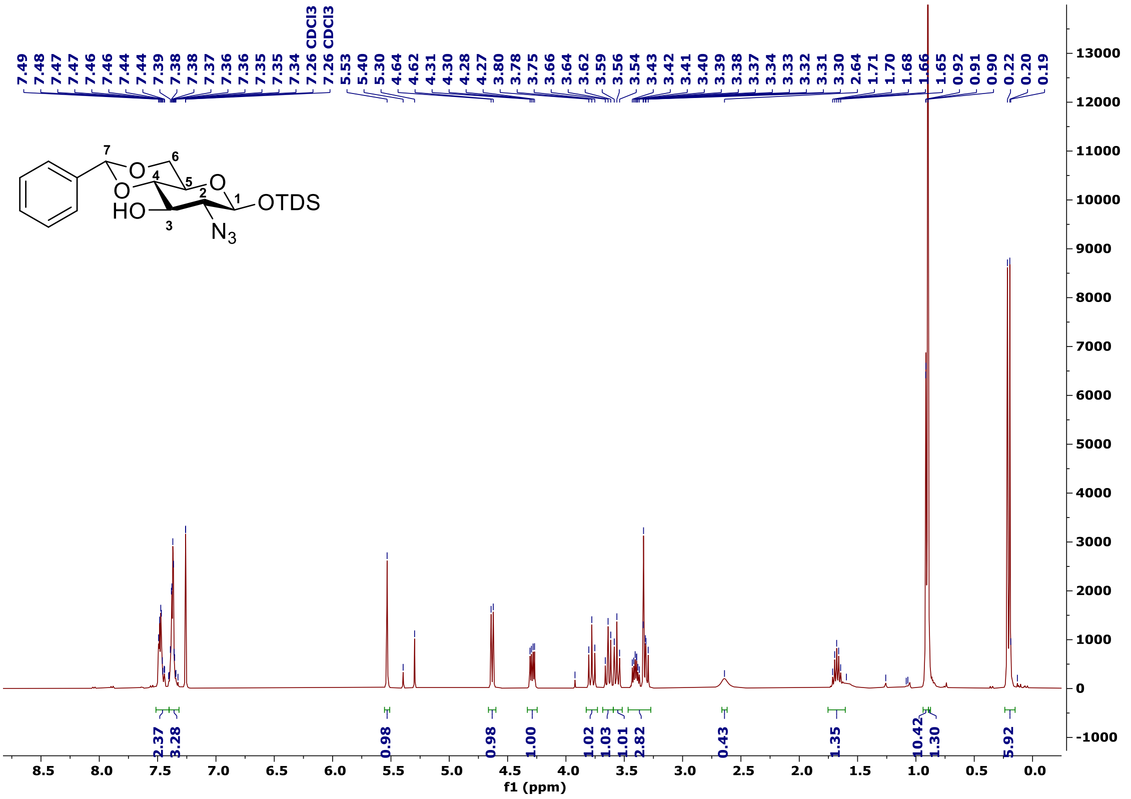

^{13}C-NMR (400 MHz, $CDCl_3$)

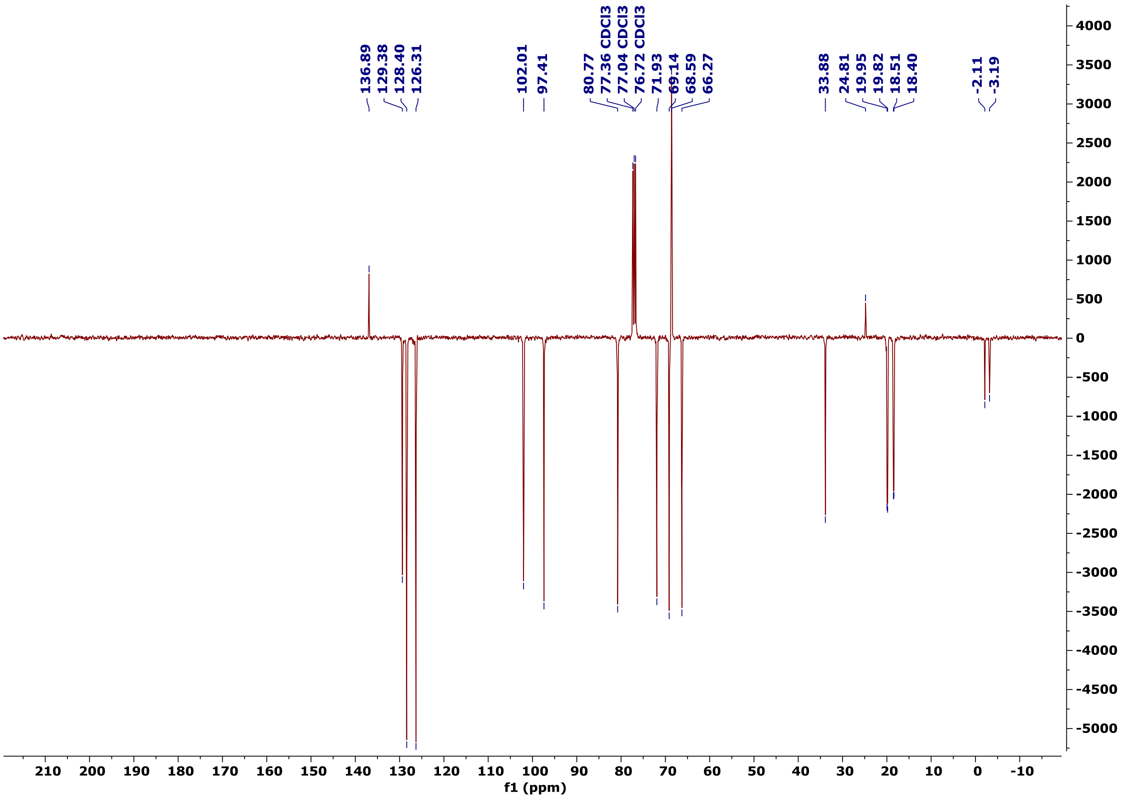

Compound 13

^{1}H-NMR (400 MHz, $CDCl_3$)

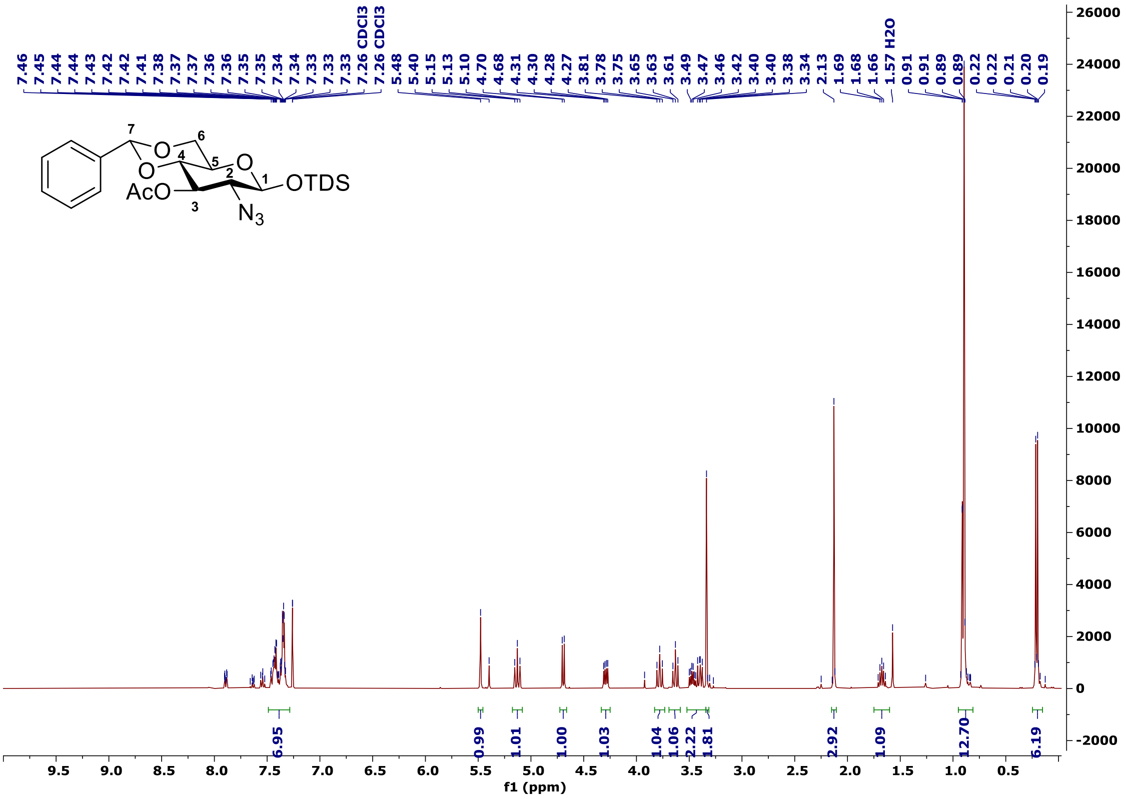

^{13}C-NMR (400 MHz, $CDCl_3$)

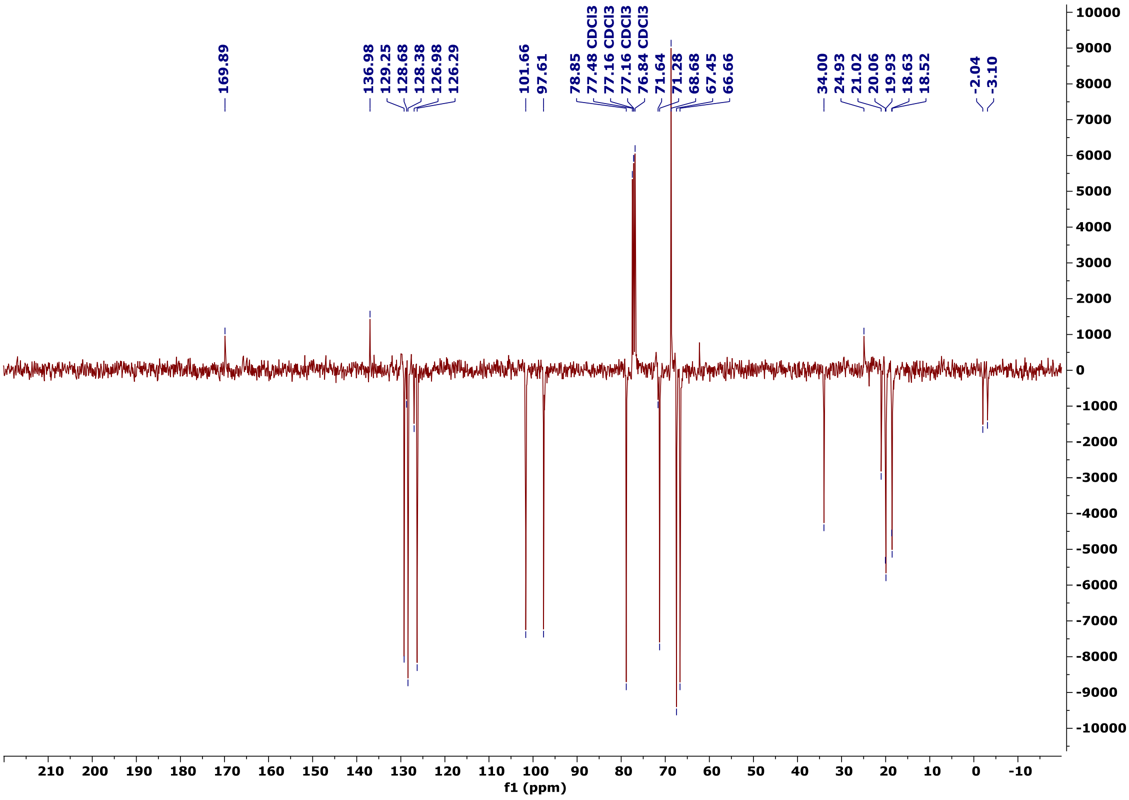

Compound 2

^{1}H-NMR (400 MHz, $CDCl_3$)

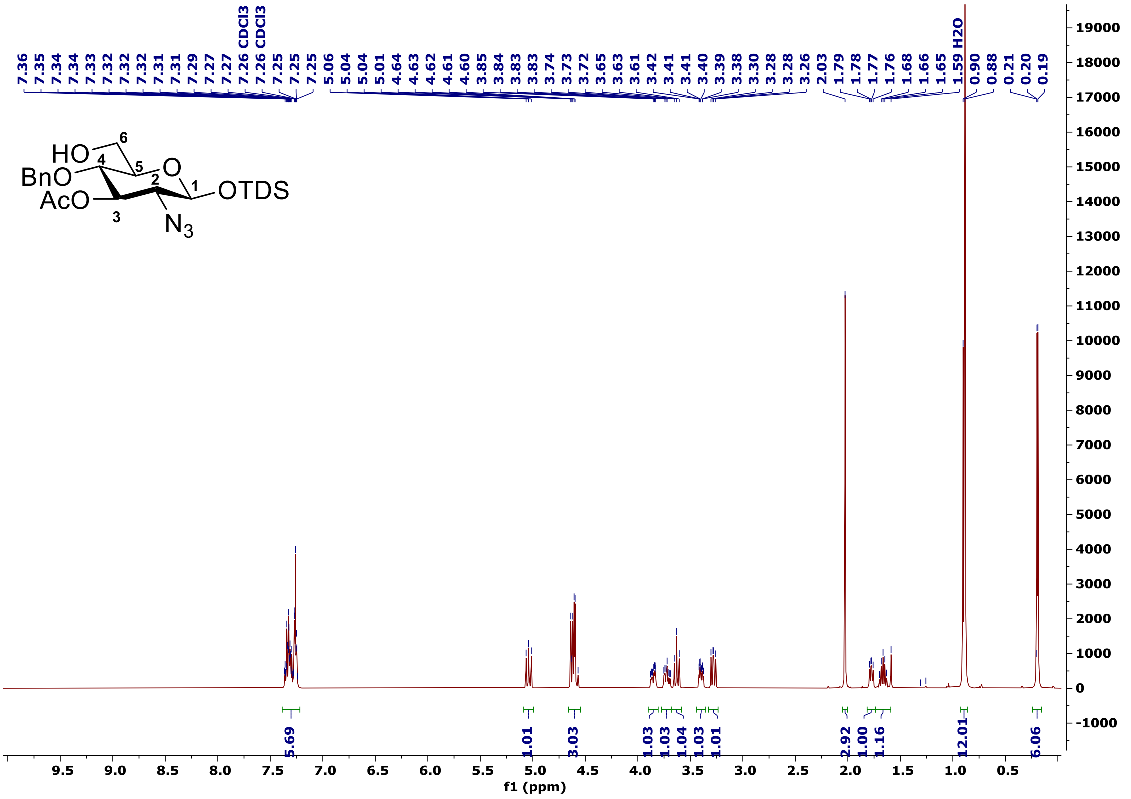

^{13}C-NMR (400 MHz, $CDCl_3$)

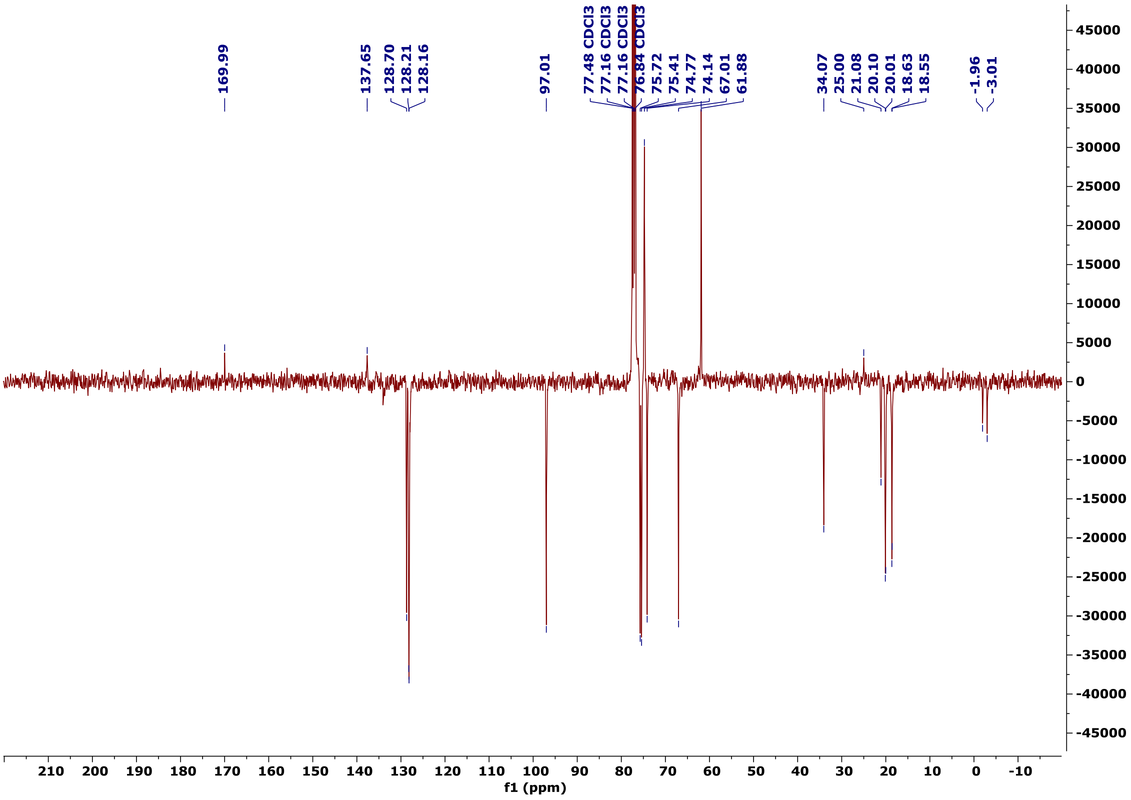

Compound 14

^{1}H-NMR (400 MHz, $CDCl_3$)

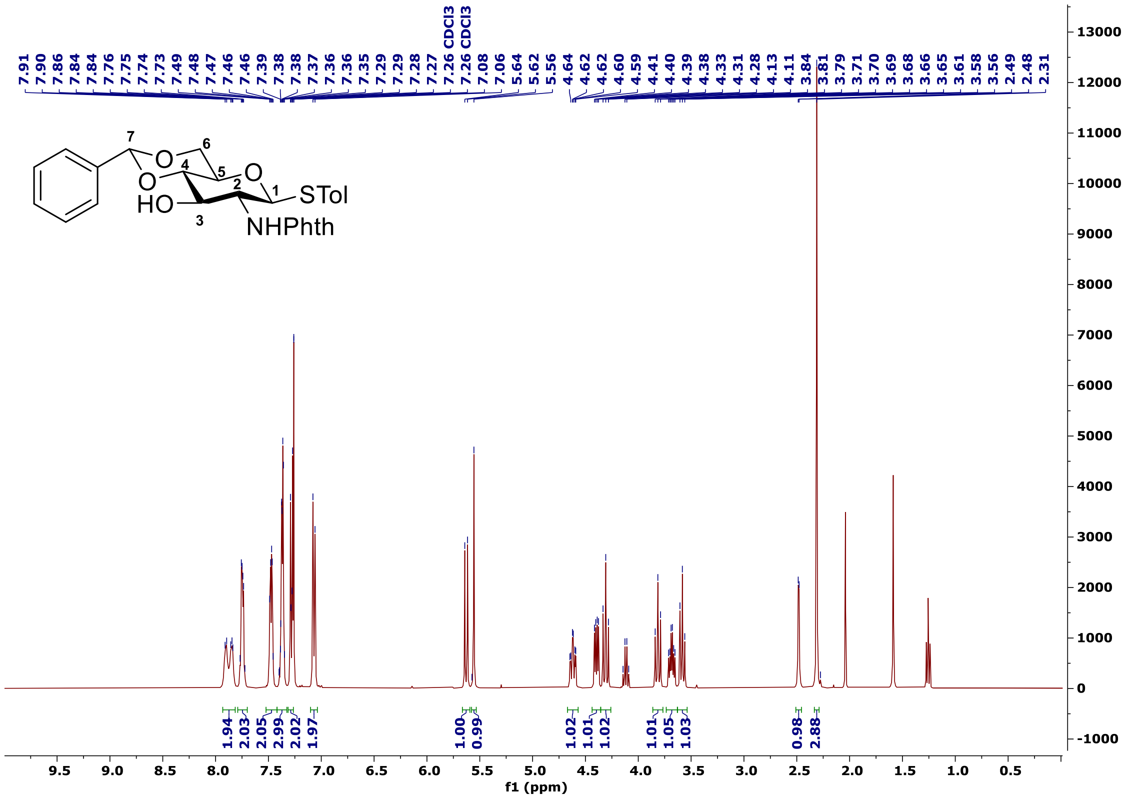

- The spectroscopic data are in agreement with those reported in literature [3].

Compound 1

^{1}H-NMR (400 MHz, $CDCl_3$)

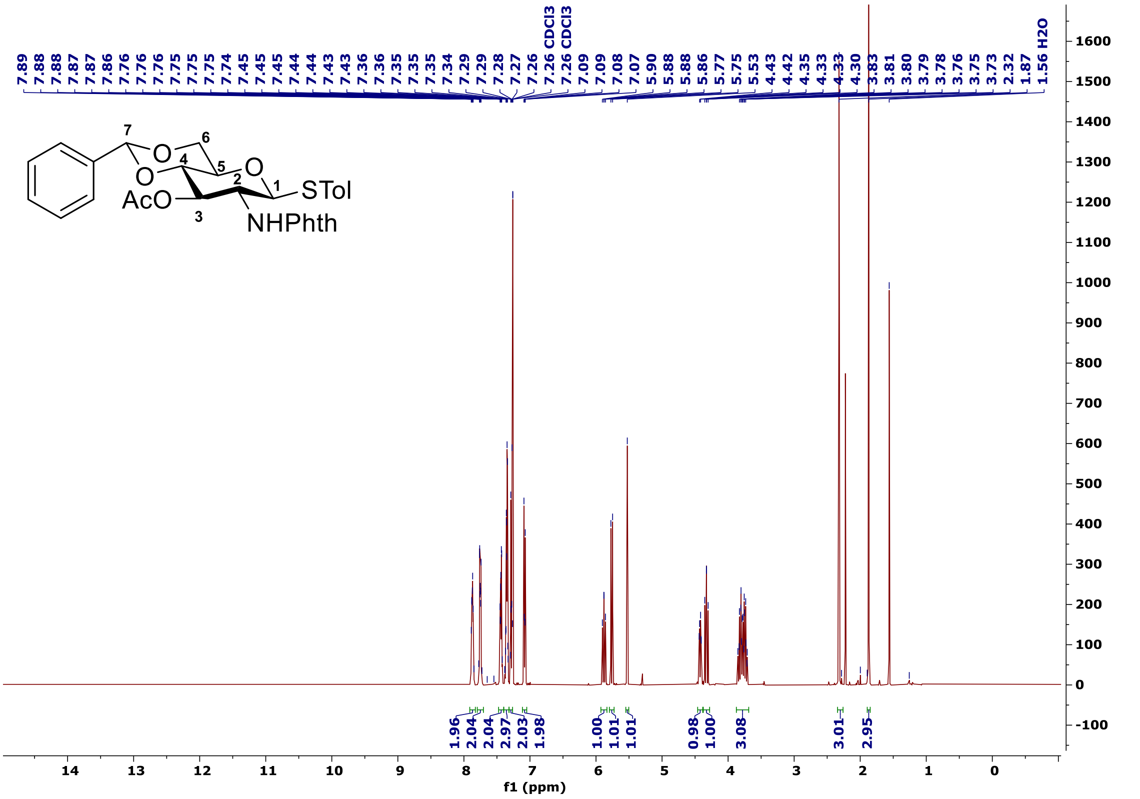

^{13}C-NMR (400 MHz, $CDCl_3$)

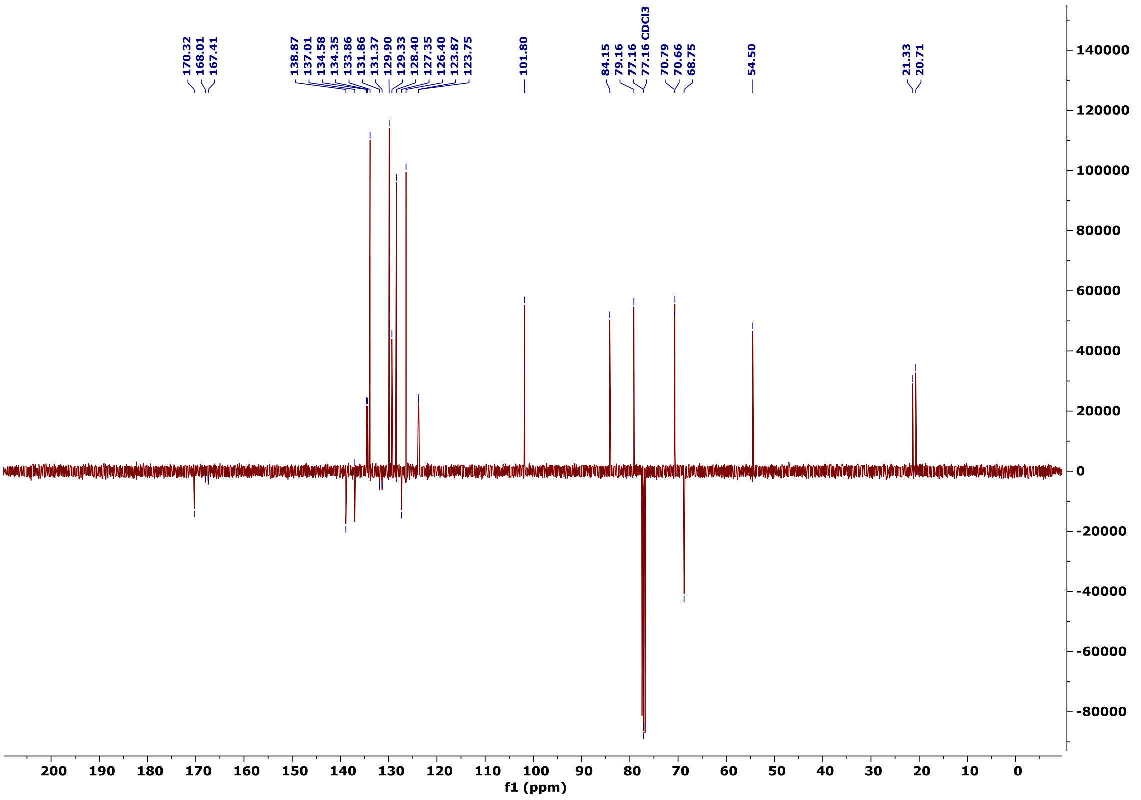

Compound 3

^{1}H-NMR (400 MHz, CDCl$_3$)

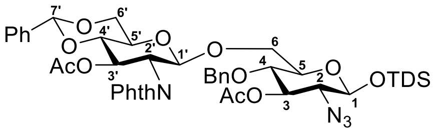

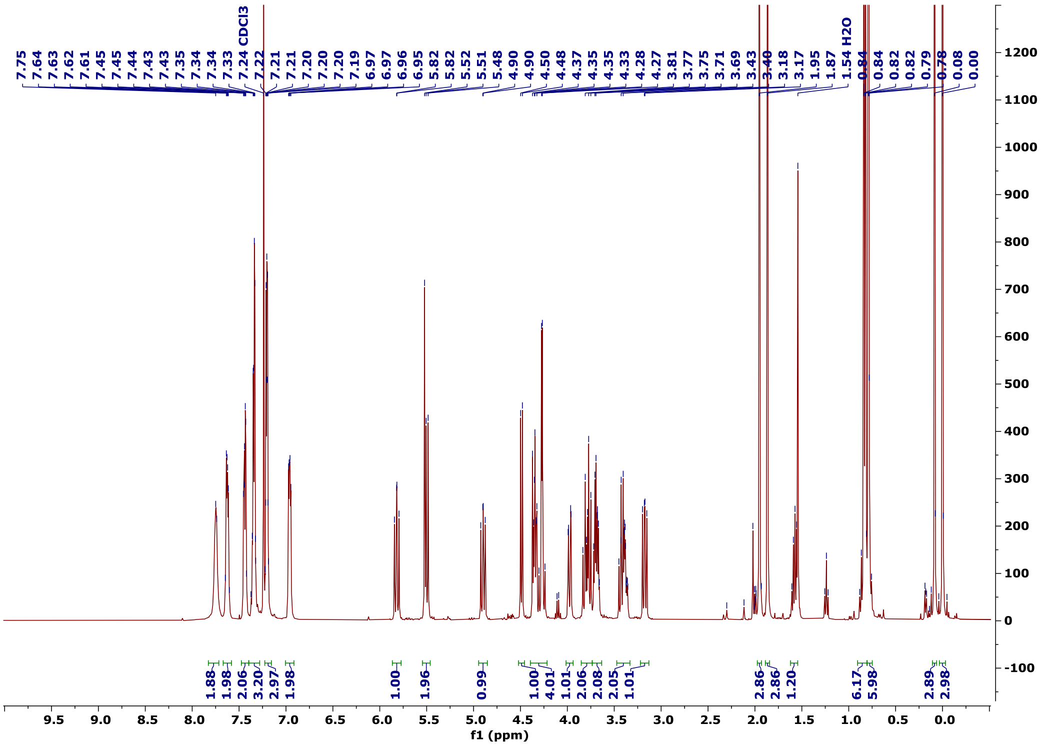

^{13}C-NMR (400 MHz, CDCl$_3$)

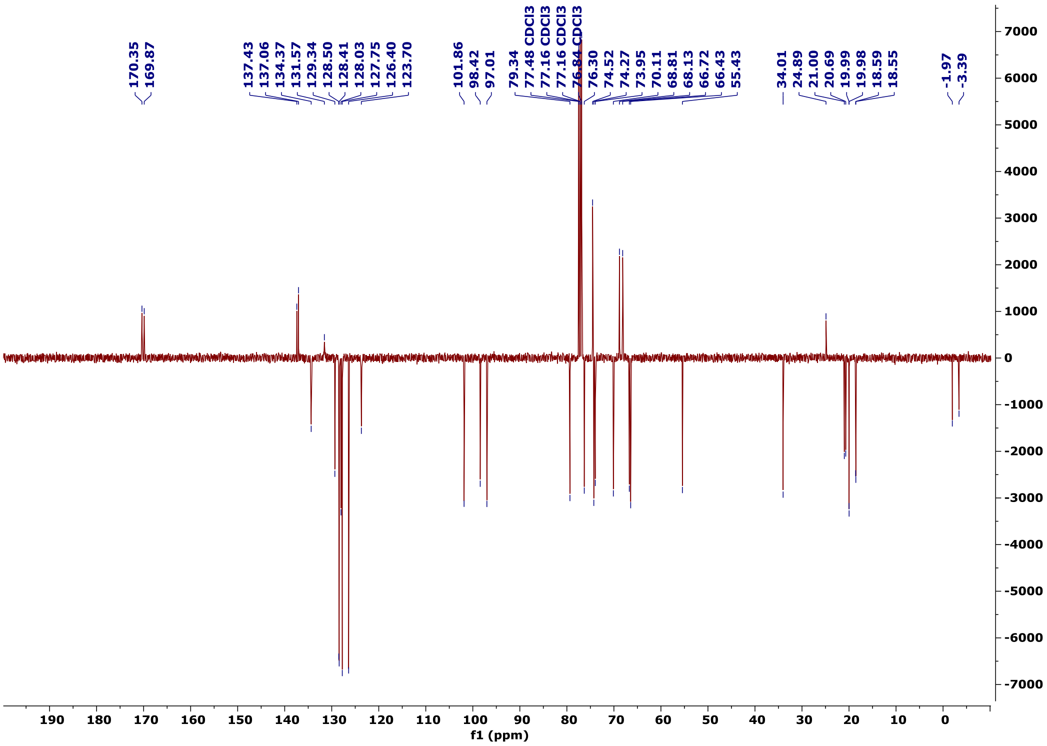

^1H-^1H COSY NMR (400 MHz, $CDCl_3$)

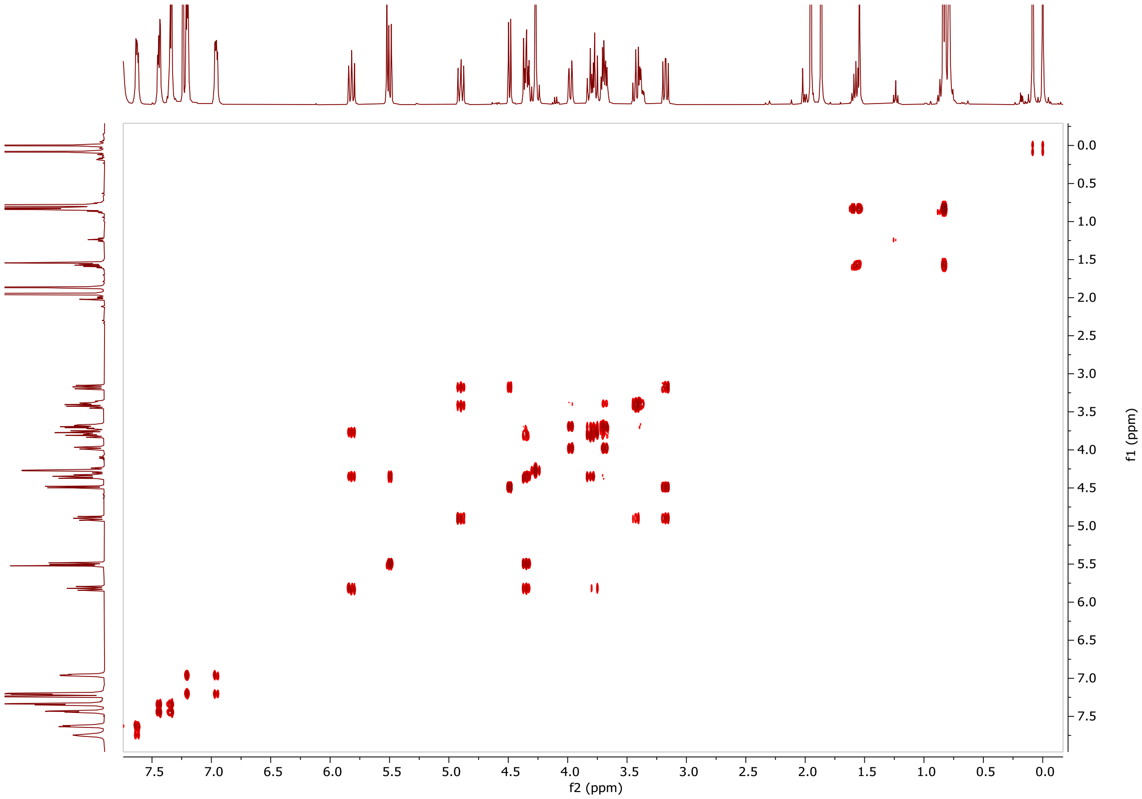

^1H-^{13}C HSQC NMR (400 MHz, $CDCl_3$)

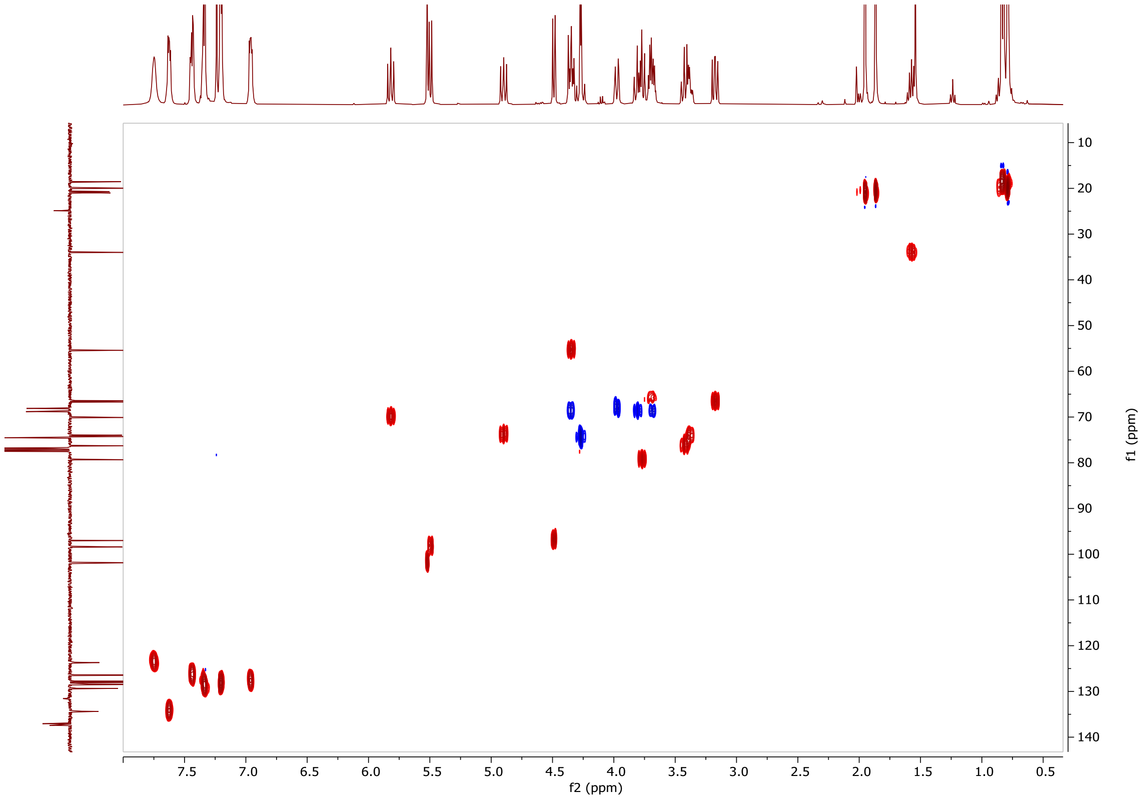

Compound 15

^{1}H-NMR (400 MHz, $CDCl_3$)

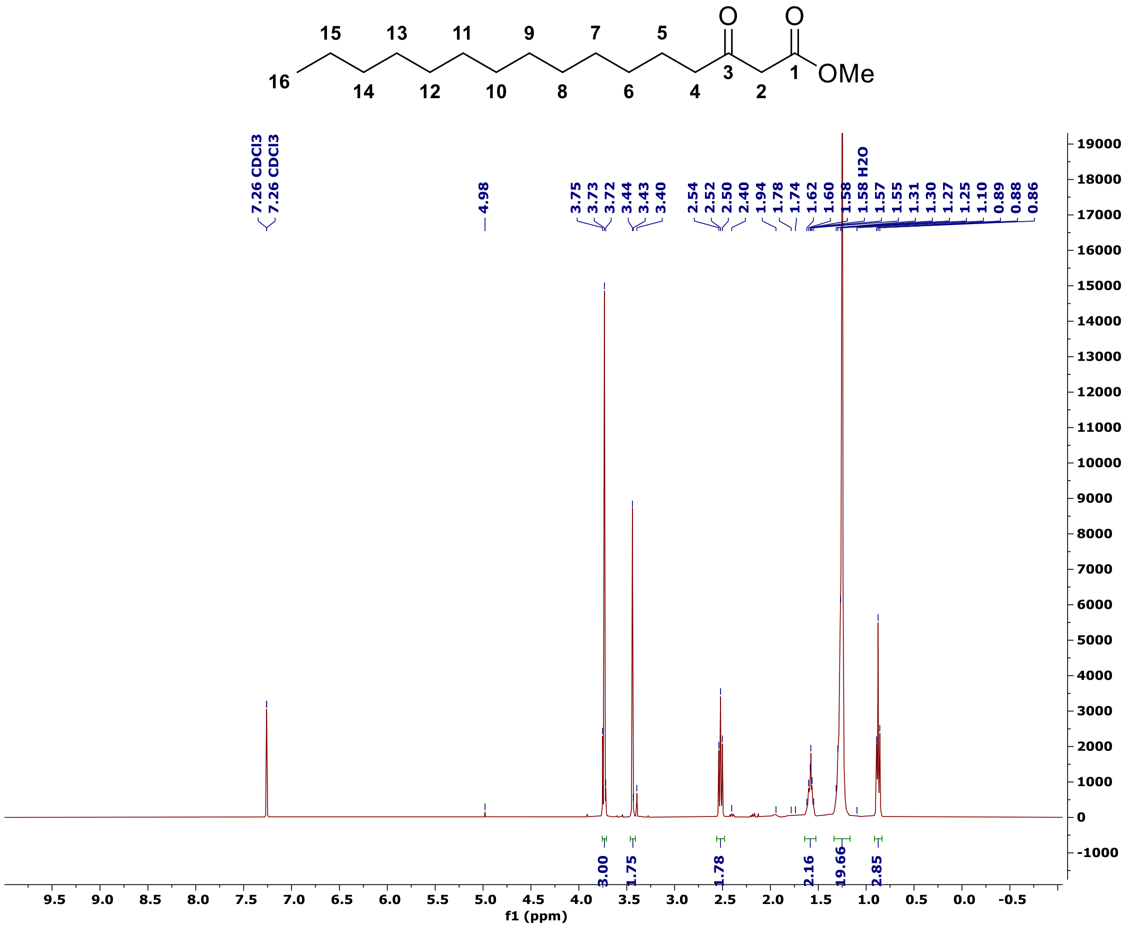

- The spectroscopic data are in agreement with those reported in literature [4].

Compound 16

^{1}H-NMR (400 MHz, $CDCl_3$)

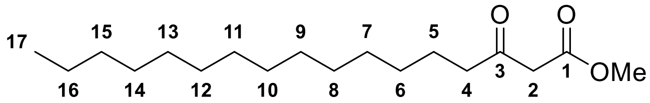

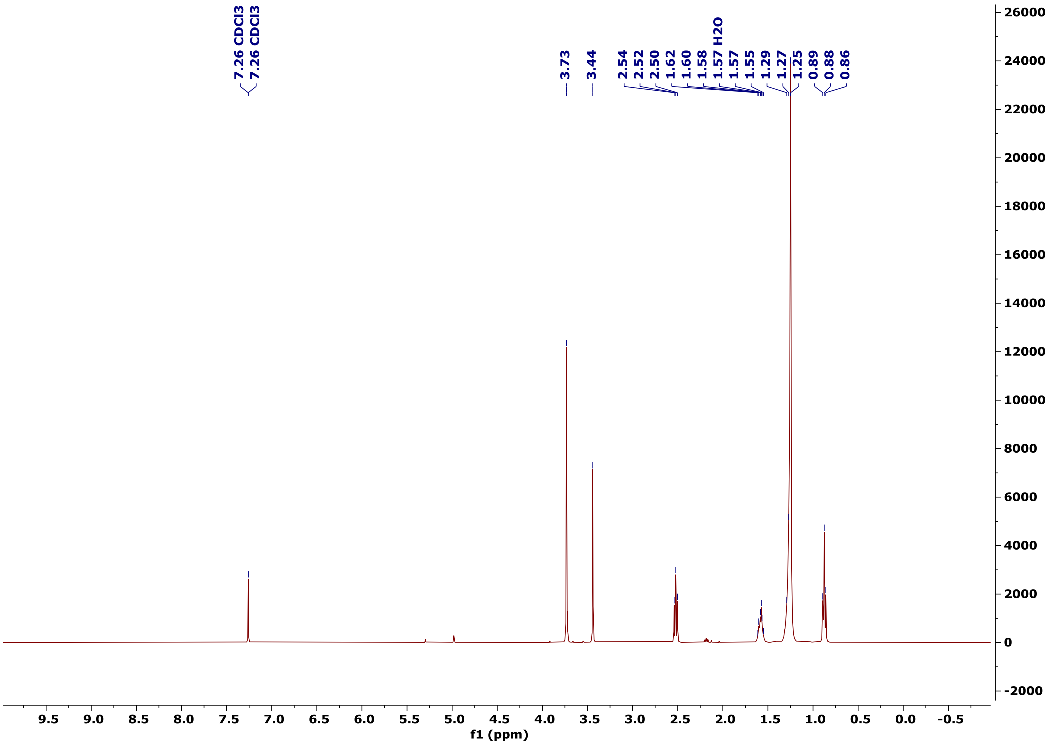

^{13}C-NMR (400 MHz, $CDCl_3$)

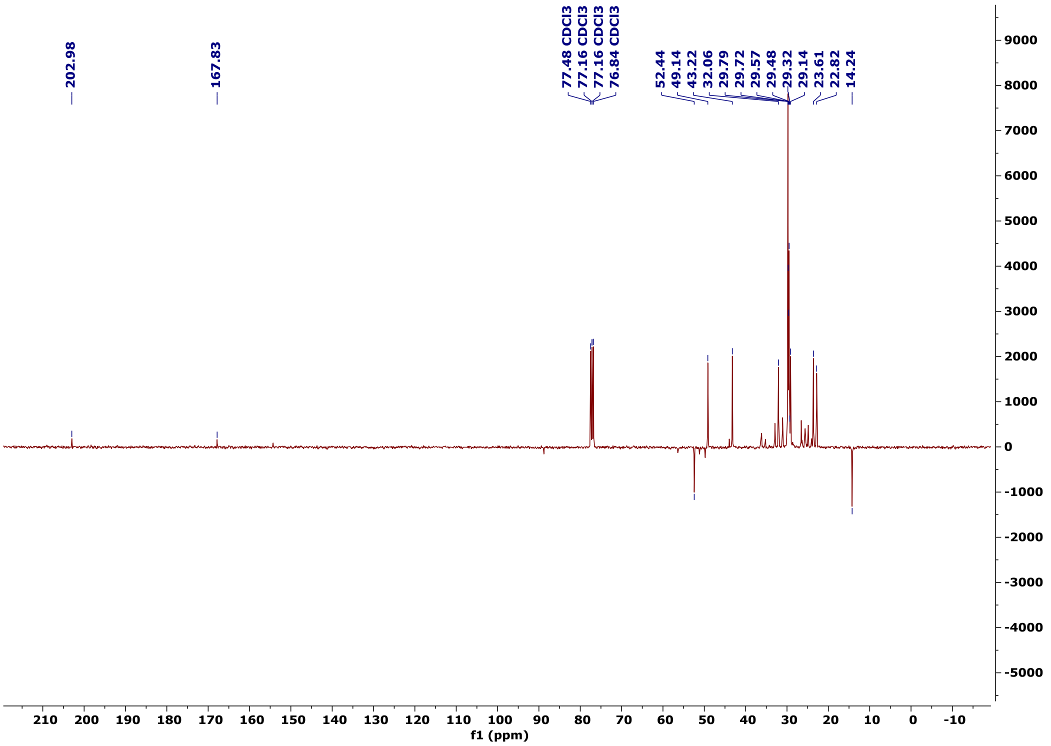

Compound 17

^{1}H-NMR (400 MHz, $CDCl_3$)

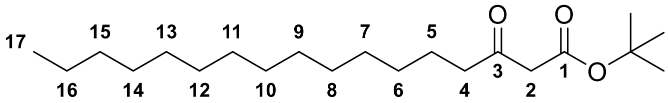

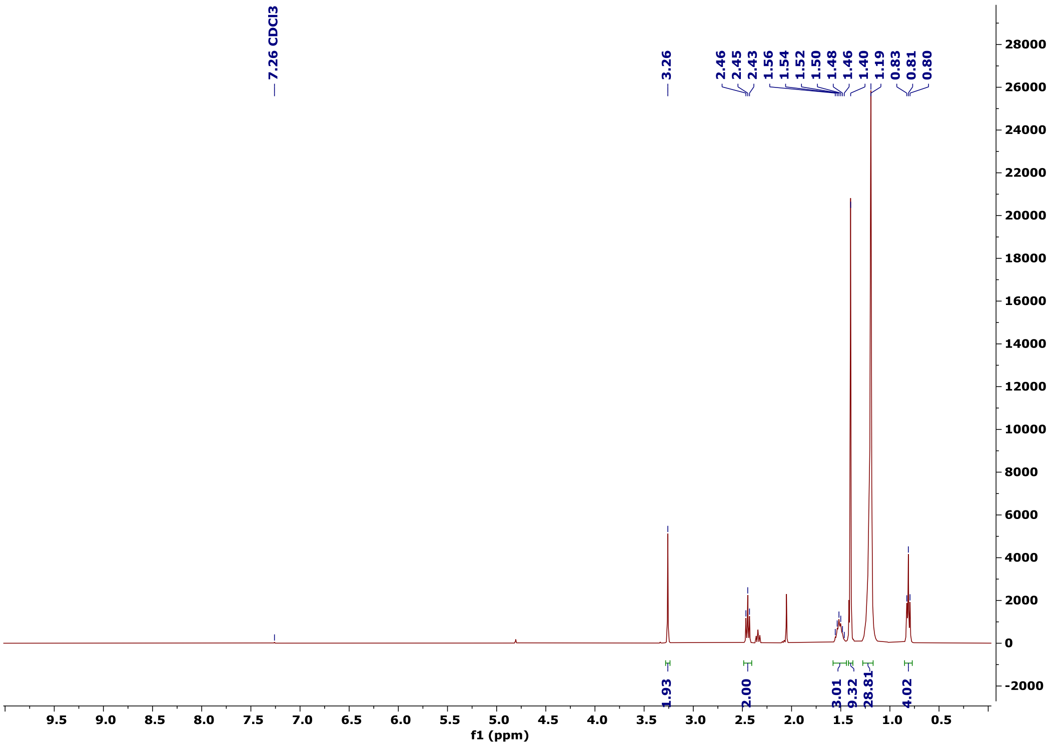

^{13}C-NMR (400 MHz, $CDCl_3$)

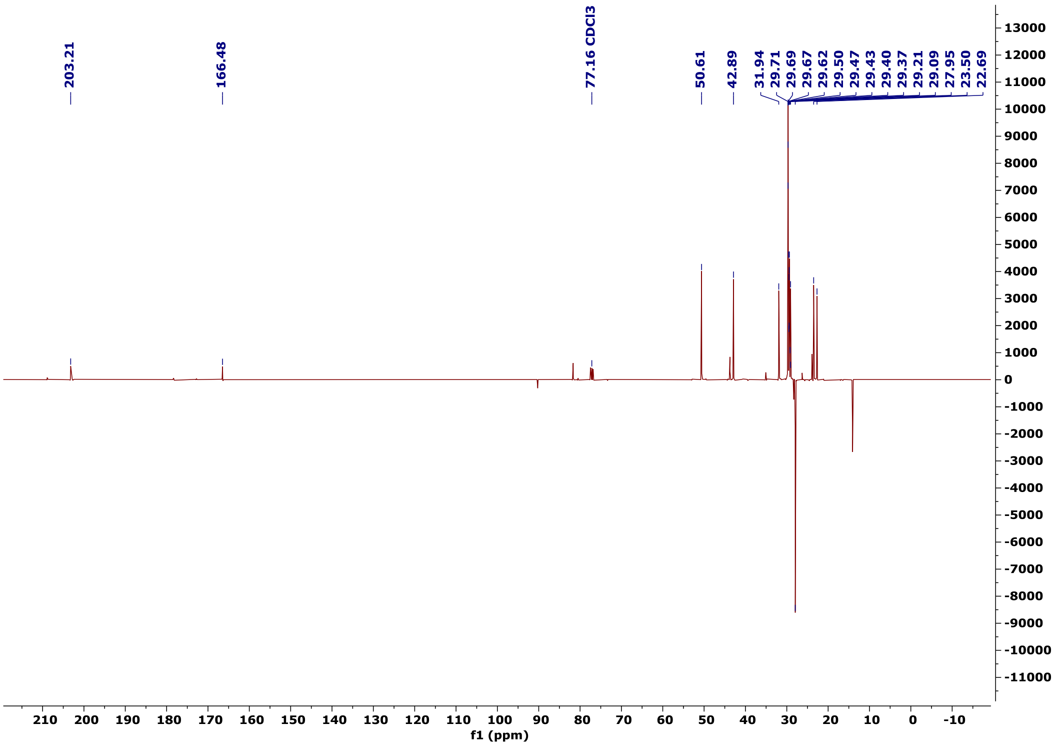

Compound 18

^{1}H-NMR (400 MHz, $CDCl_3$)

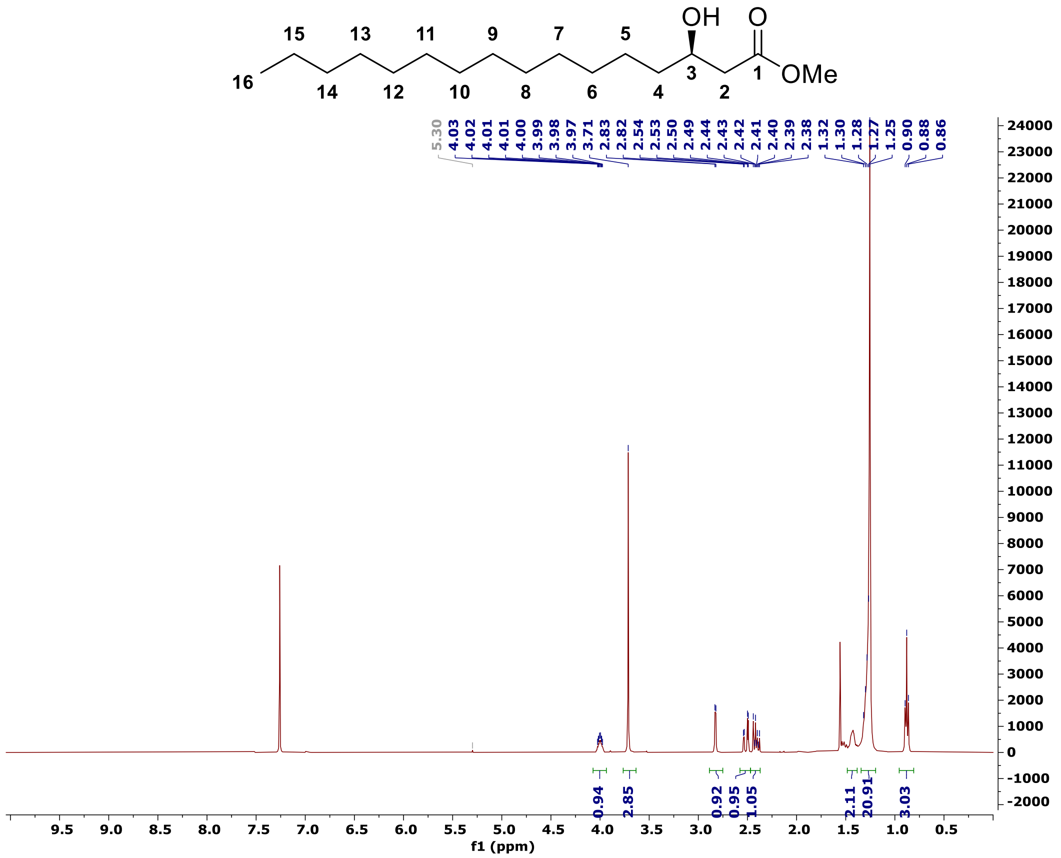

- The spectroscopic data are in agreement with those reported in literature [5].

Mosher's ester analysis by ^{1}H-NMR [6]

Compound **18** was reacted with (R)- and (S)-MTPA chloride according to this scheme:

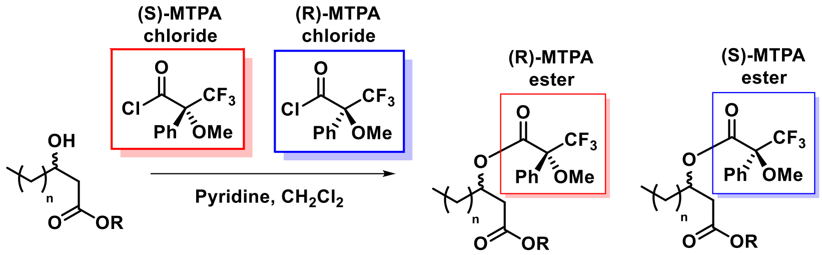

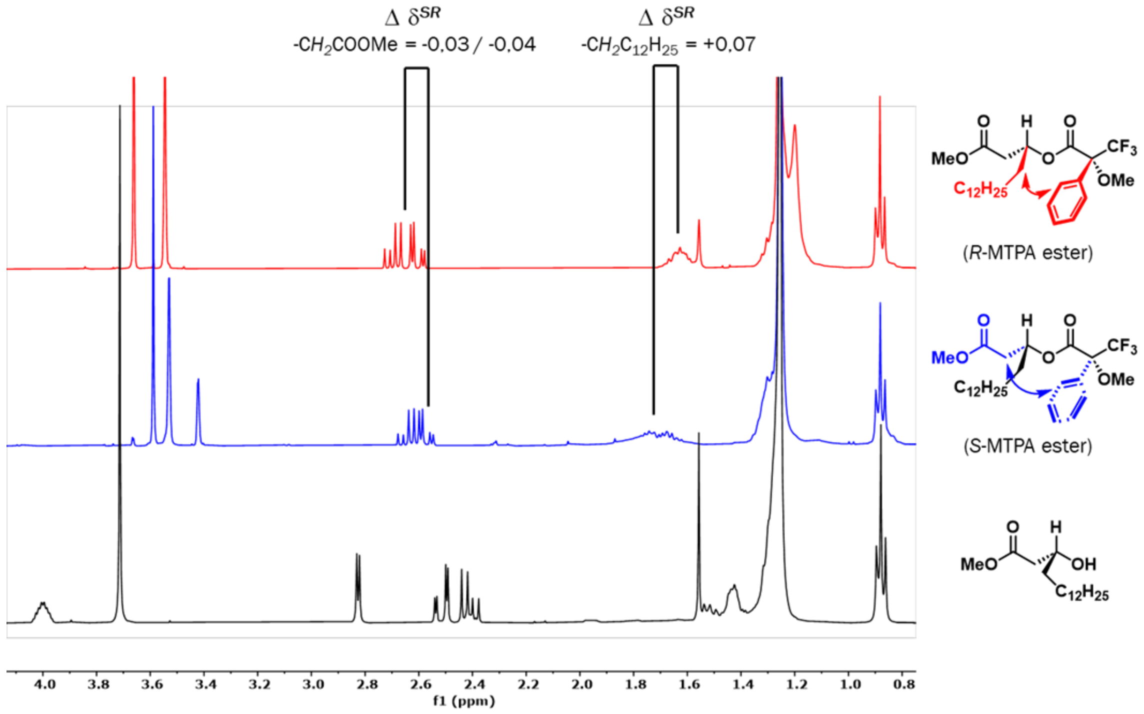

Determination of configuration

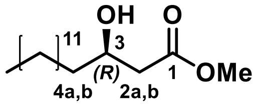

$\Delta\delta^{S-R}$ = δ (*S*-MTPA) - δ (*R*-MTPA)

Proton	Chemical shift (S)	Chemical shift (R)	$\Delta\delta^{S-R}$
2a	2.65	2.70	- 0.04
2b	2.57	2.60	- 0.03
3	5.47	5.47	0
4a, 4b	1.70	1.63	+ 0.07
OMe	3.59	3.66	- 0.07

$\Delta\delta^{S-R}$ **H-4a,b (R1) = + 0.07 Positive**
$\Delta\delta^{S-R}$ **H-2a,2b (R2) = - 0.07 Negative**

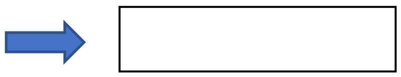

NMR studies using the chiral lanthanide shift reagent Eu(hfc)$_3$ [7].

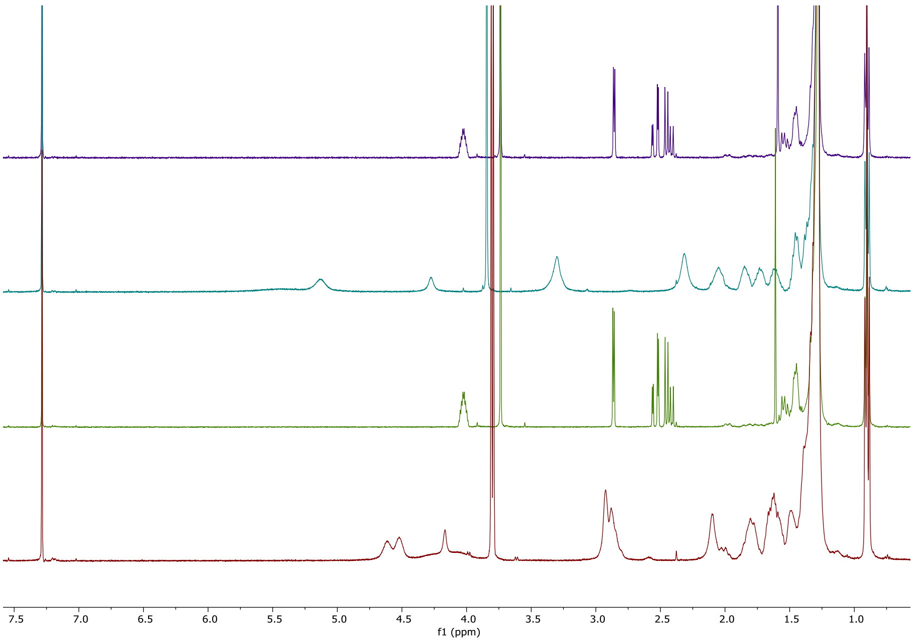

^{1}H spectrum of **18**

^{1}H spectrum of **18** + *Eu(hfc)$_3$*

^{1}H spectrum of **18** (racemic mixture after NaBH$_4$ reduction of **15**).

^{1}H spectrum of **18** (racemic mixture after NaBH$_4$ reduction of **15**) + *Eu(hfc)$_3$*

Compound 19

^{1}H-NMR (400 MHz, $CDCl_3$)

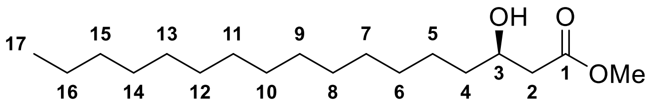

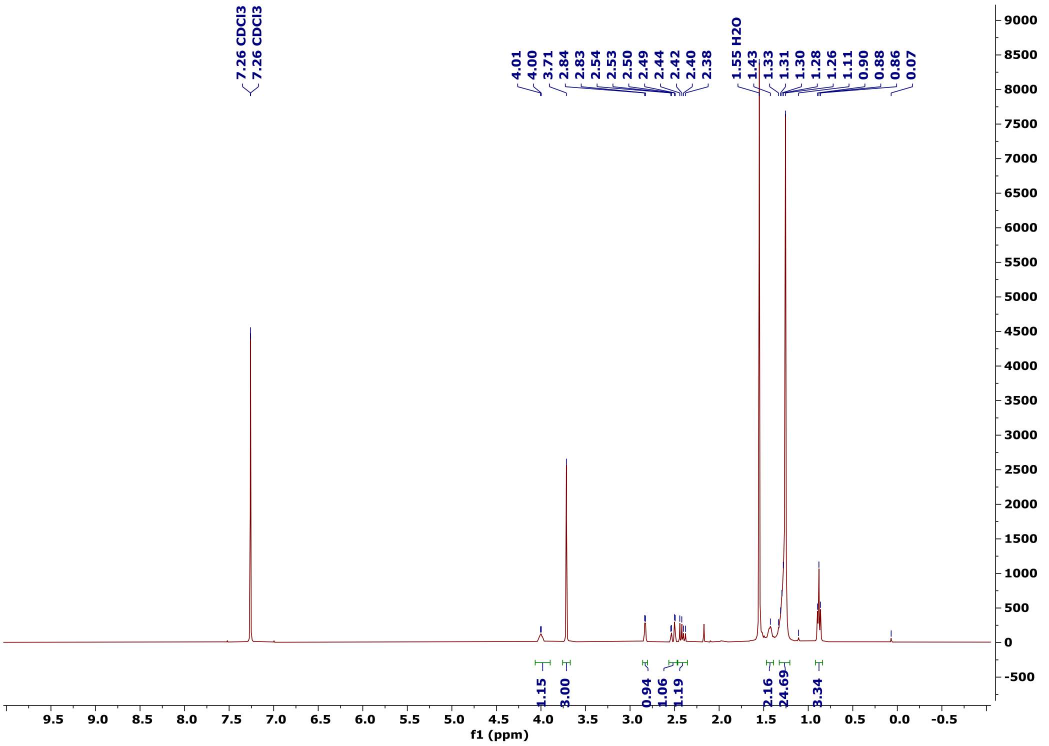

^{13}C-NMR (400 MHz, $CDCl_3$)

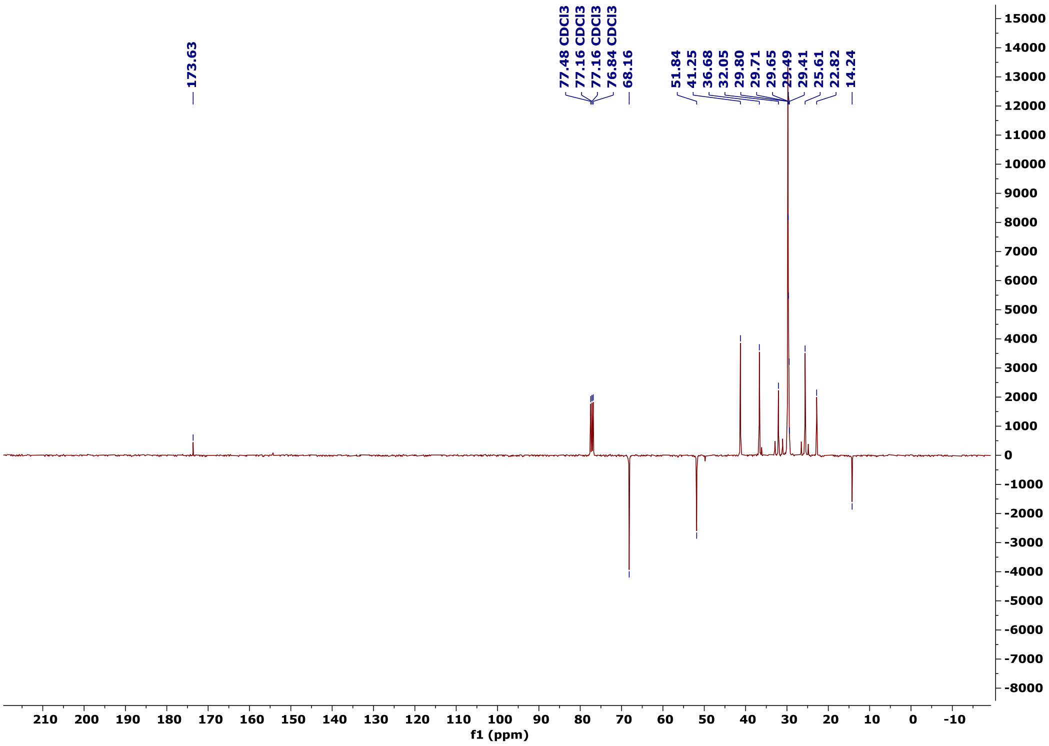

Compound 20

^{1}H-NMR (400 MHz, $CDCl_3$)

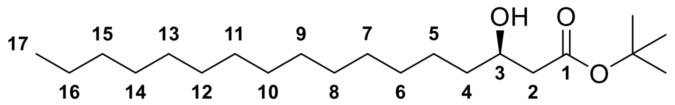

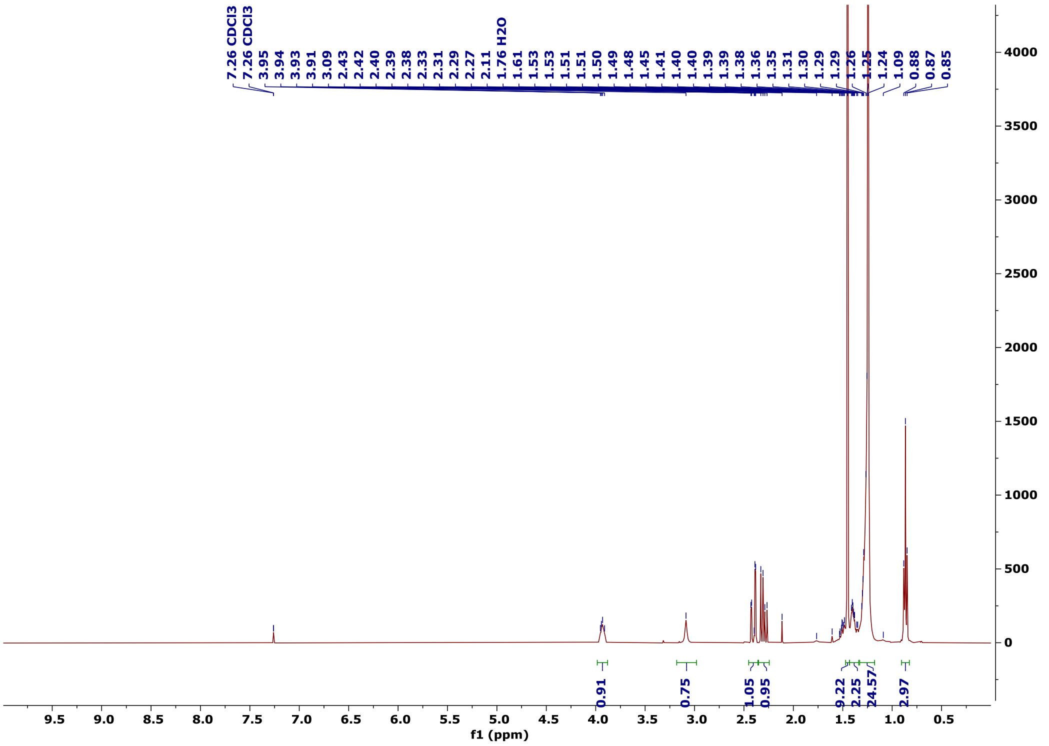

^{13}C-NMR (400 MHz, $CDCl_3$)

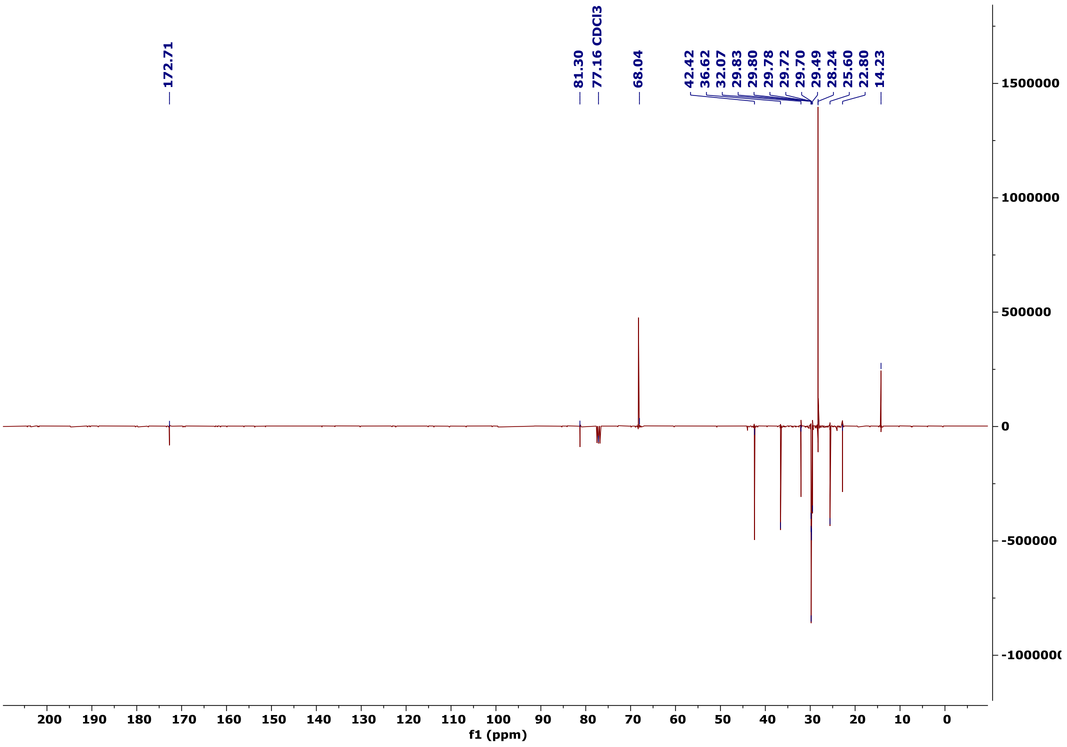

Compound 4

^{1}H-NMR (400 MHz, $CDCl_3$)

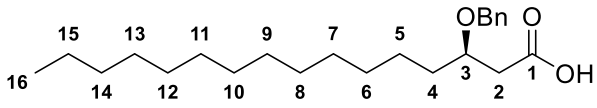

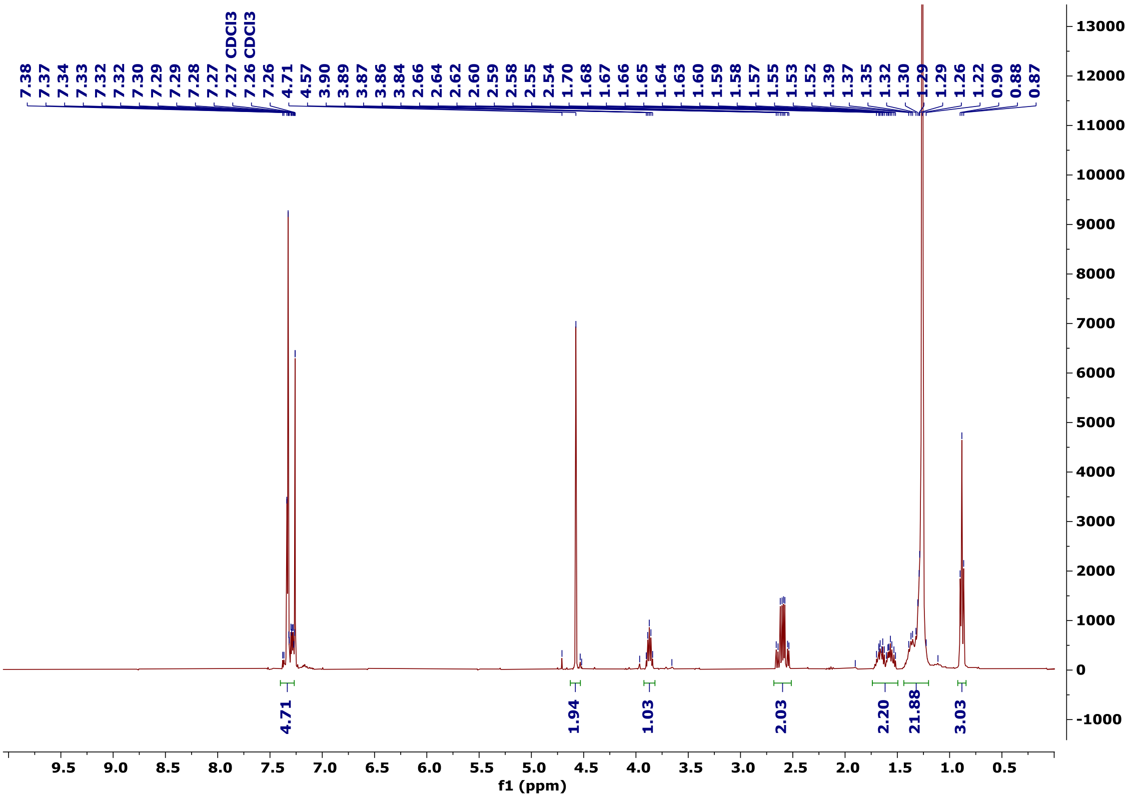

- The spectroscopic data are in agreement with those reported in literature [8].

Compound 6

^{1}H-NMR (400 MHz, $CDCl_3$)

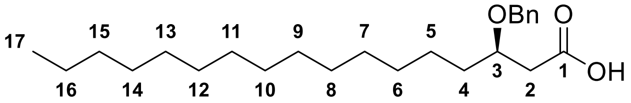

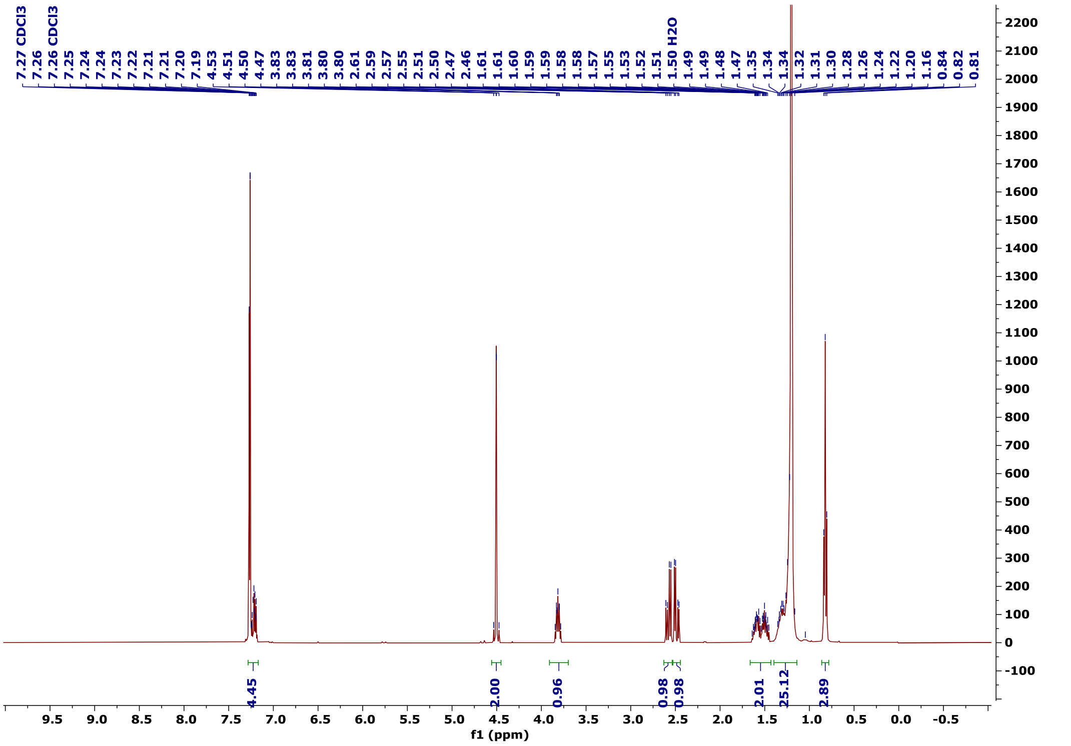

^{13}C-NMR (400 MHz, $CDCl_3$)

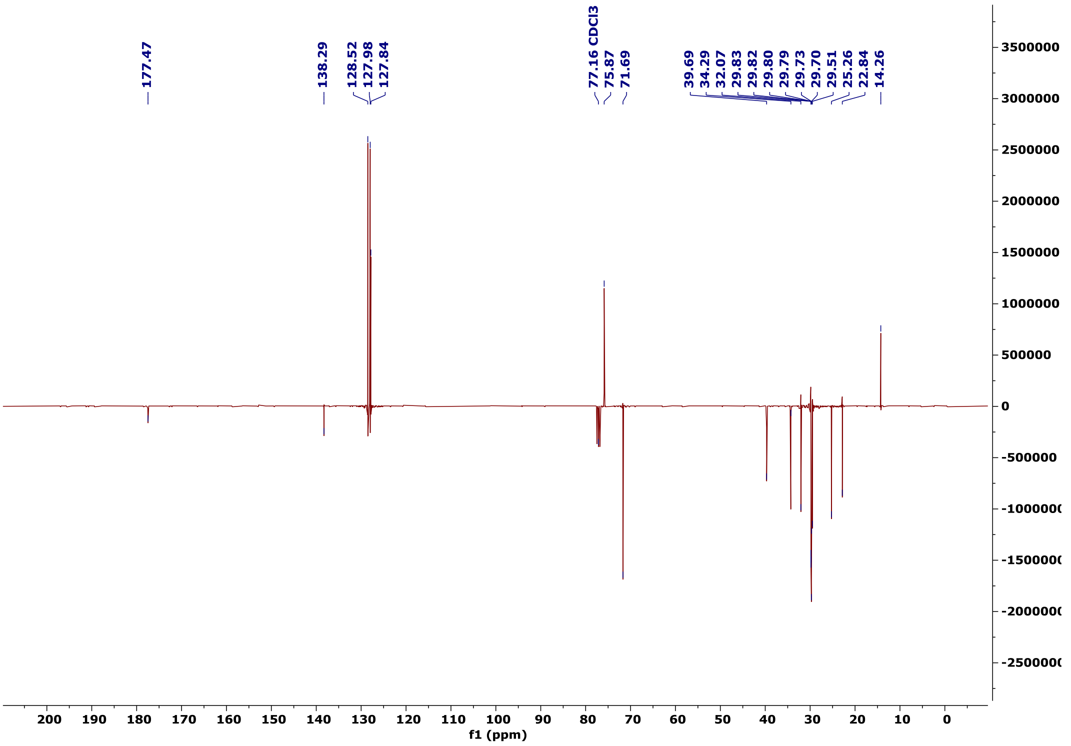

Compound 21

^{1}H-NMR (400 MHz, $CDCl_3$)

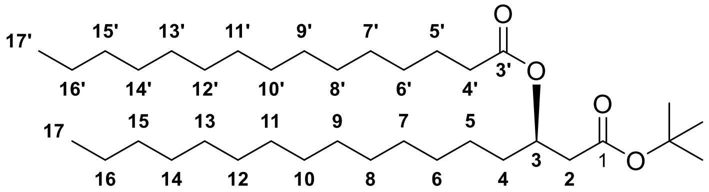

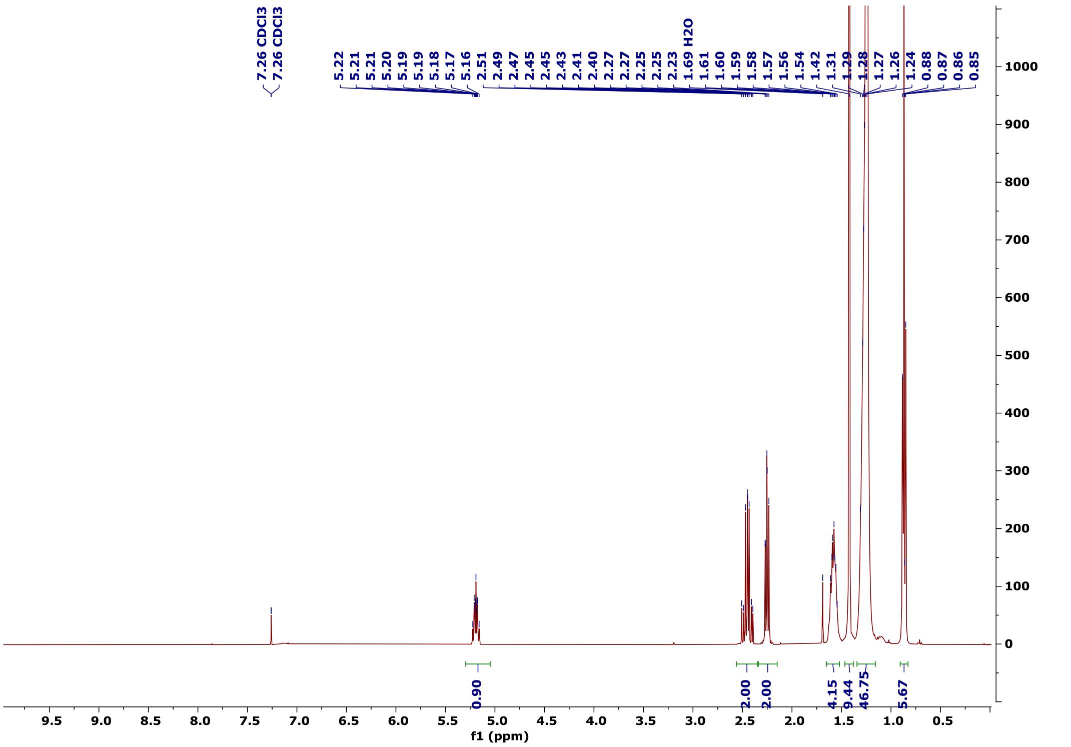

^{13}C-NMR (400 MHz, $CDCl_3$)

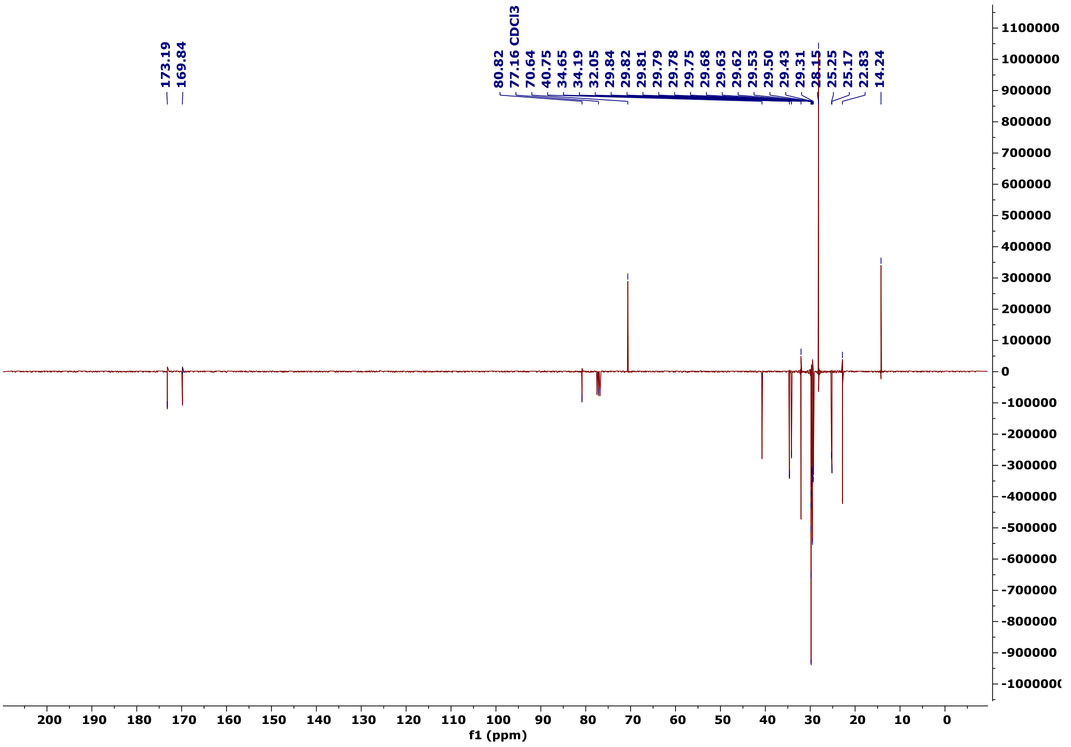

Compound 7

^{1}H-NMR (400 MHz, $CDCl_3$)

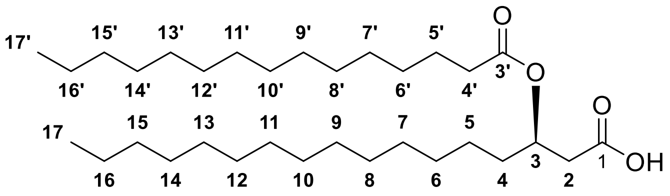

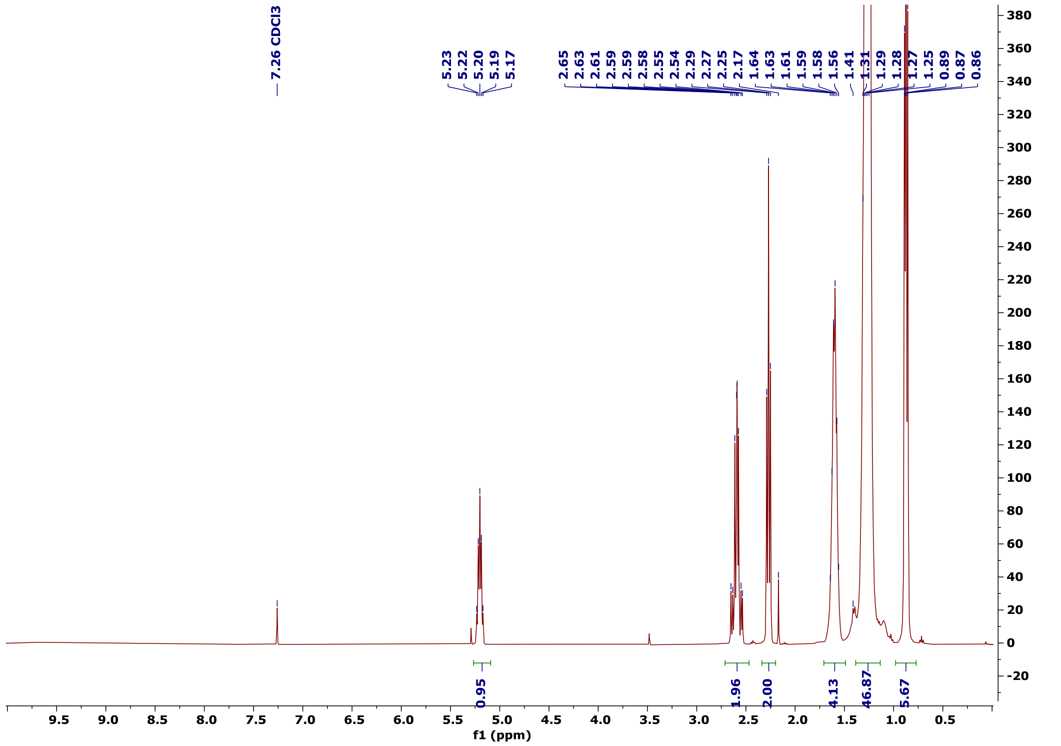

^{13}C-NMR (400 MHz, $CDCl_3$)

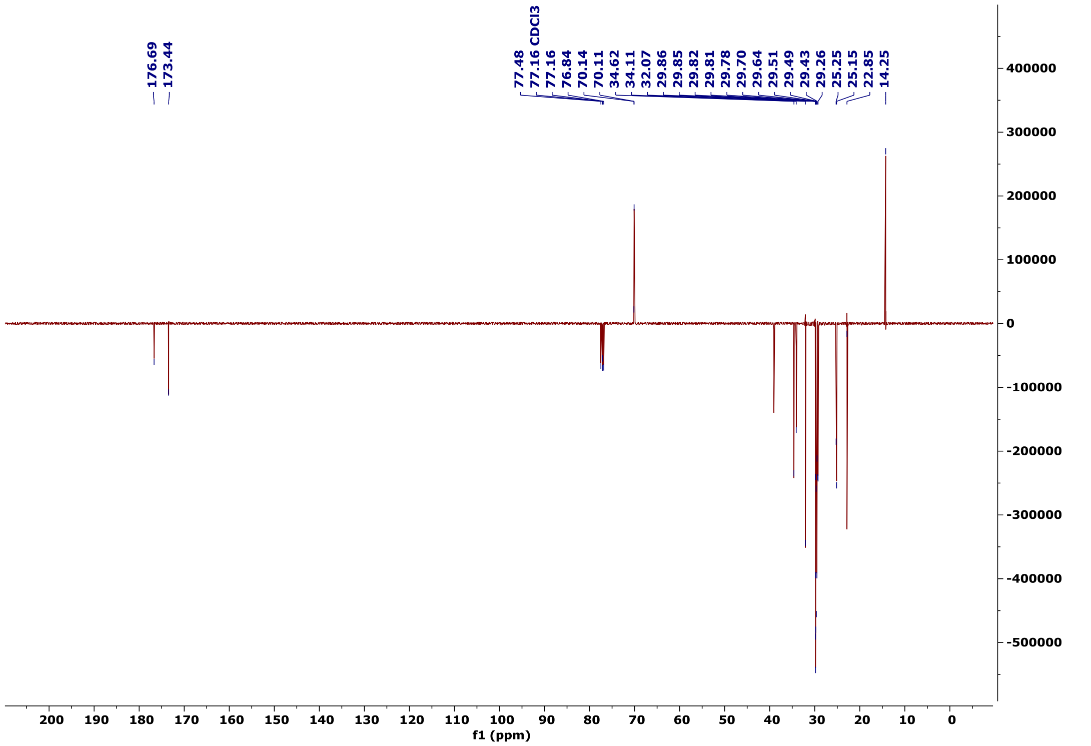

Compound 23

^{1}H-NMR (400 MHz, $CDCl_3$)

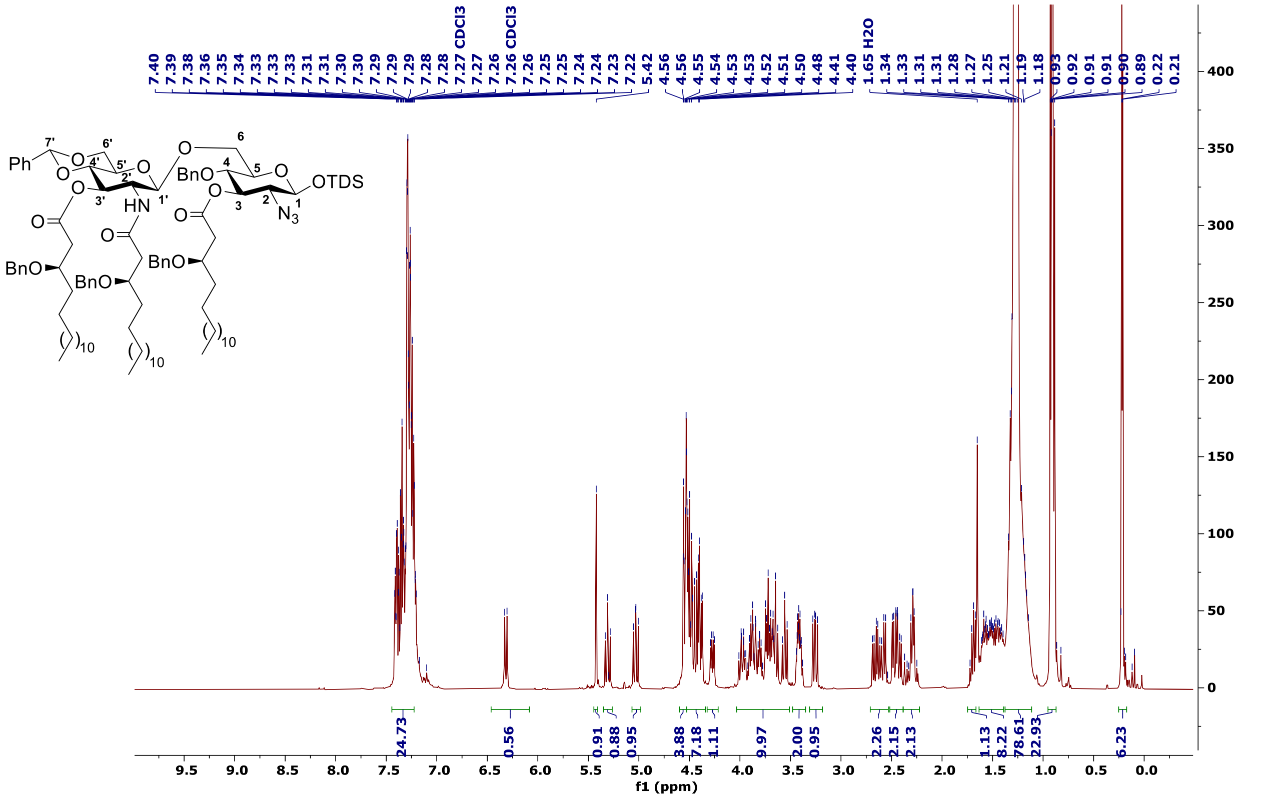

^{13}C-NMR (400 MHz, $CDCl_3$)

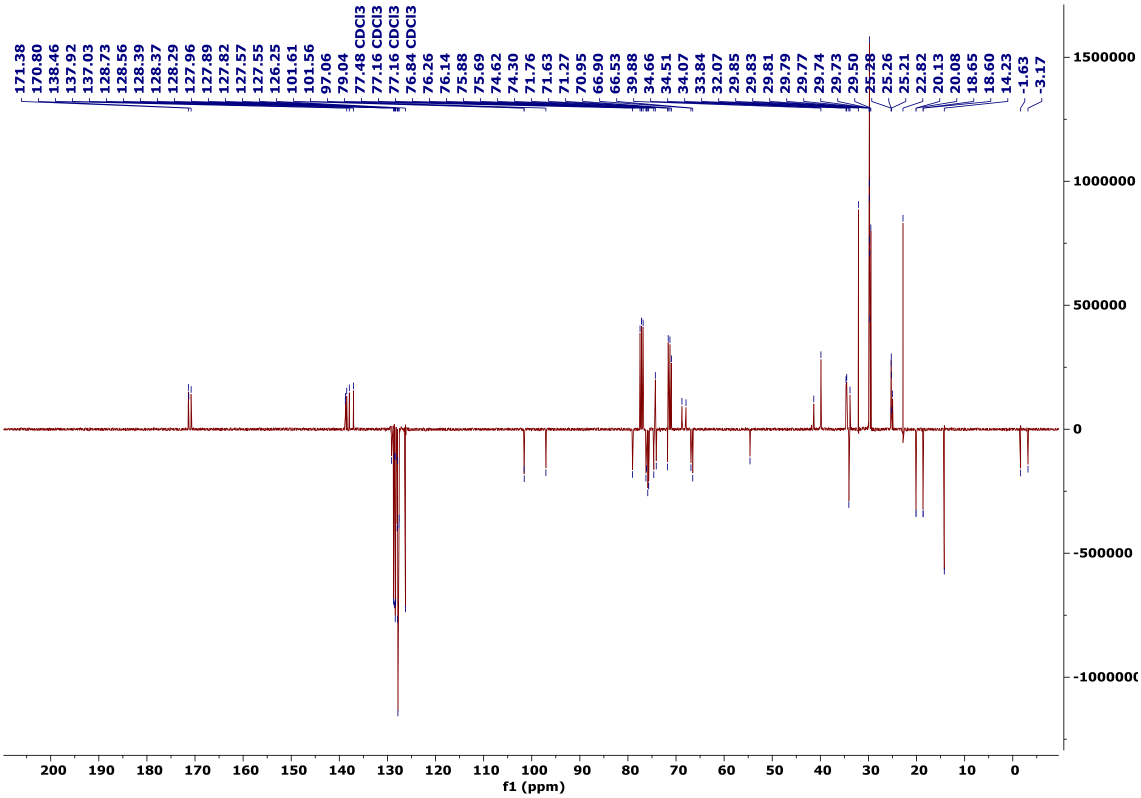

Compound 24

^{1}H-NMR (400 MHz, $CDCl_3$)

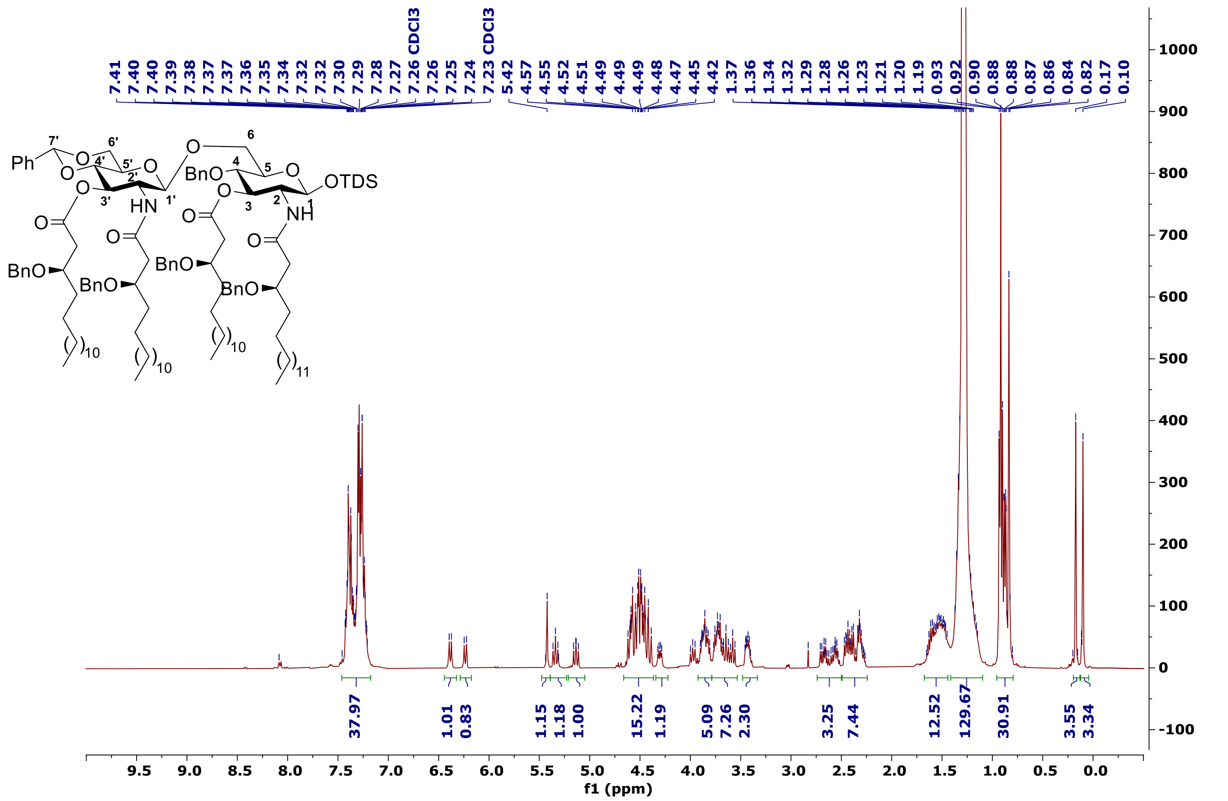

^{13}C-NMR (400 MHz, $CDCl_3$)

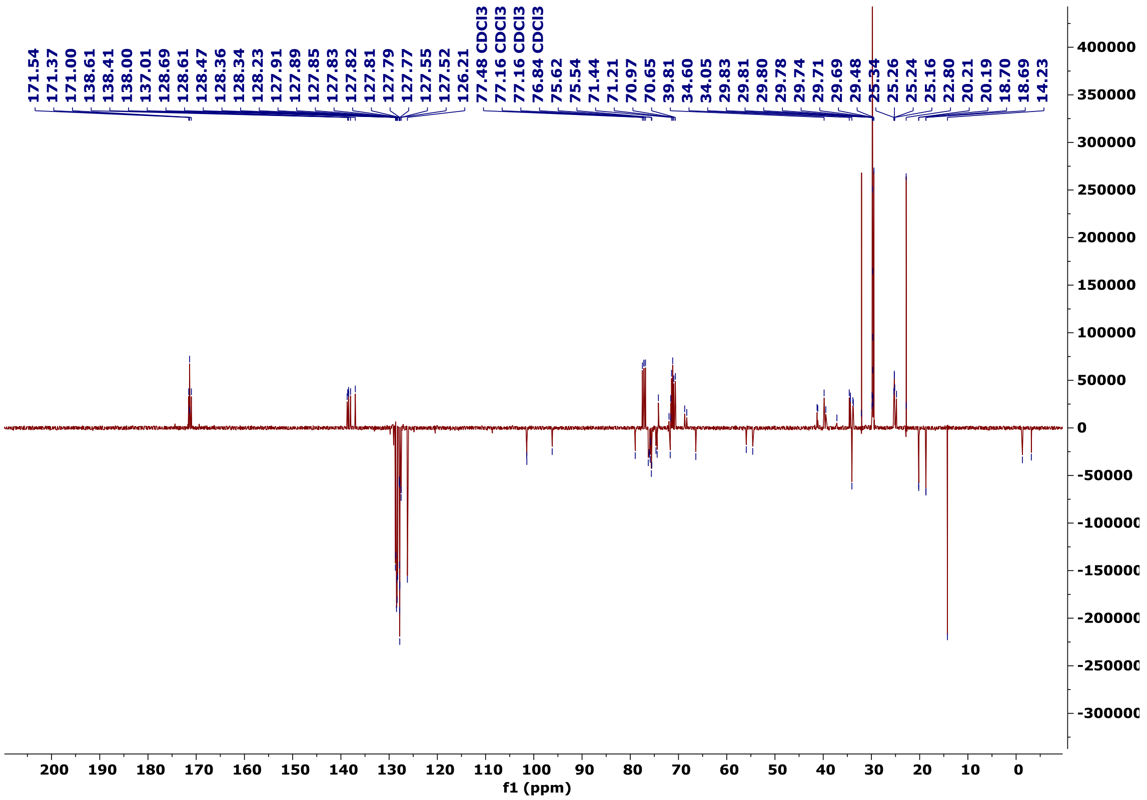

Compound 27

^{1}H-NMR (400 MHz, $CDCl_3$)

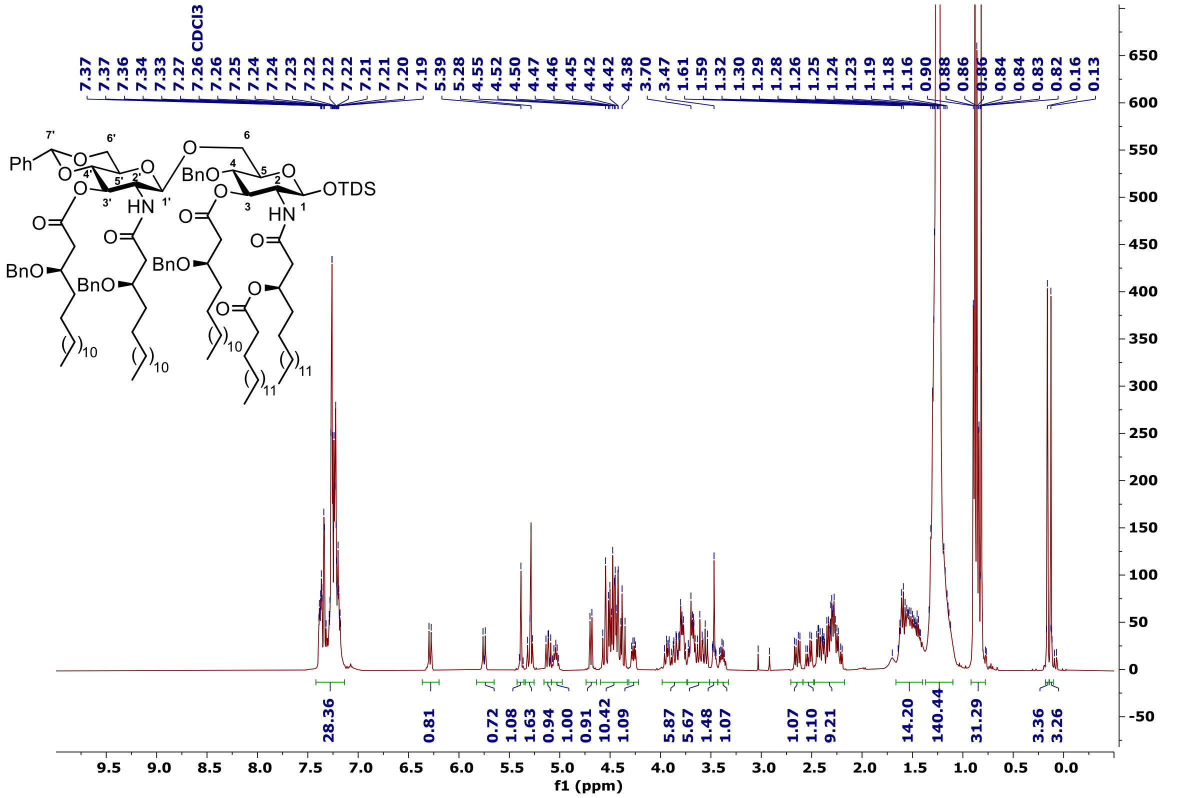

^{13}C-NMR (400 MHz, $CDCl_3$)

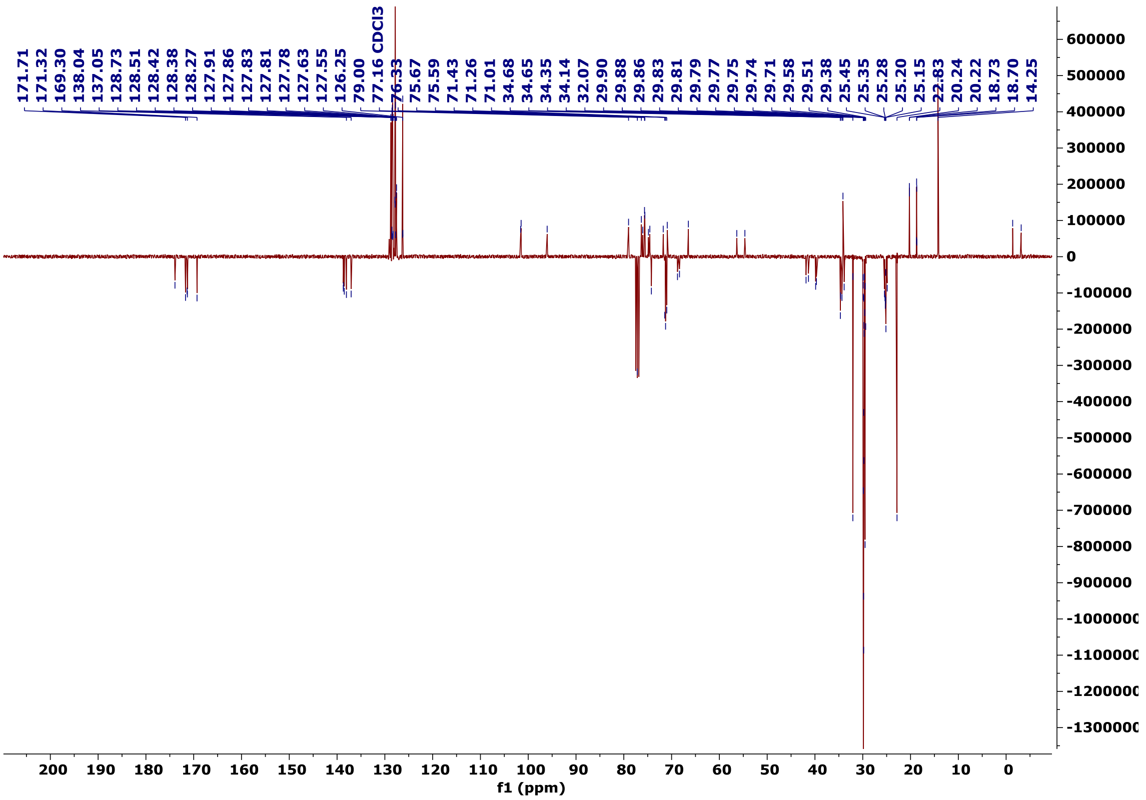

Compound 25

^{1}H-NMR (400 MHz, $CDCl_3$)

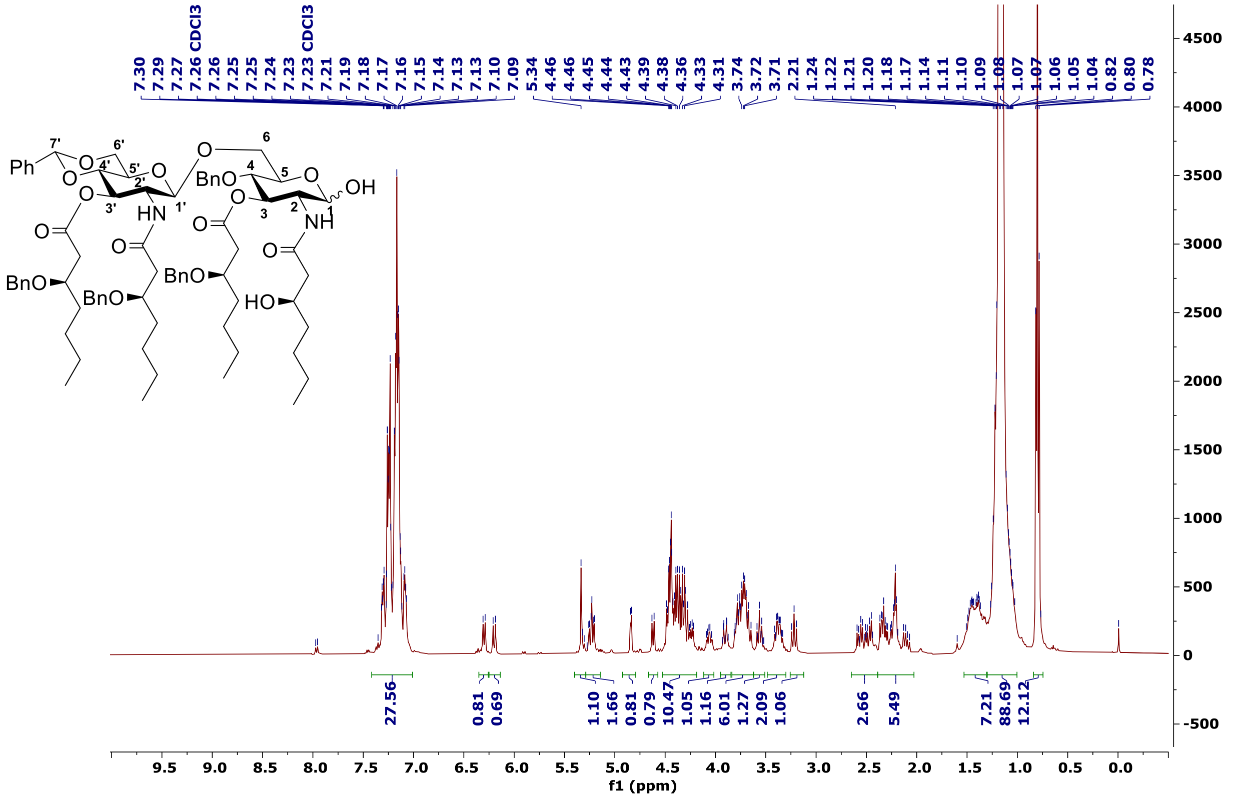

^{13}C-NMR (400 MHz, $CDCl_3$)

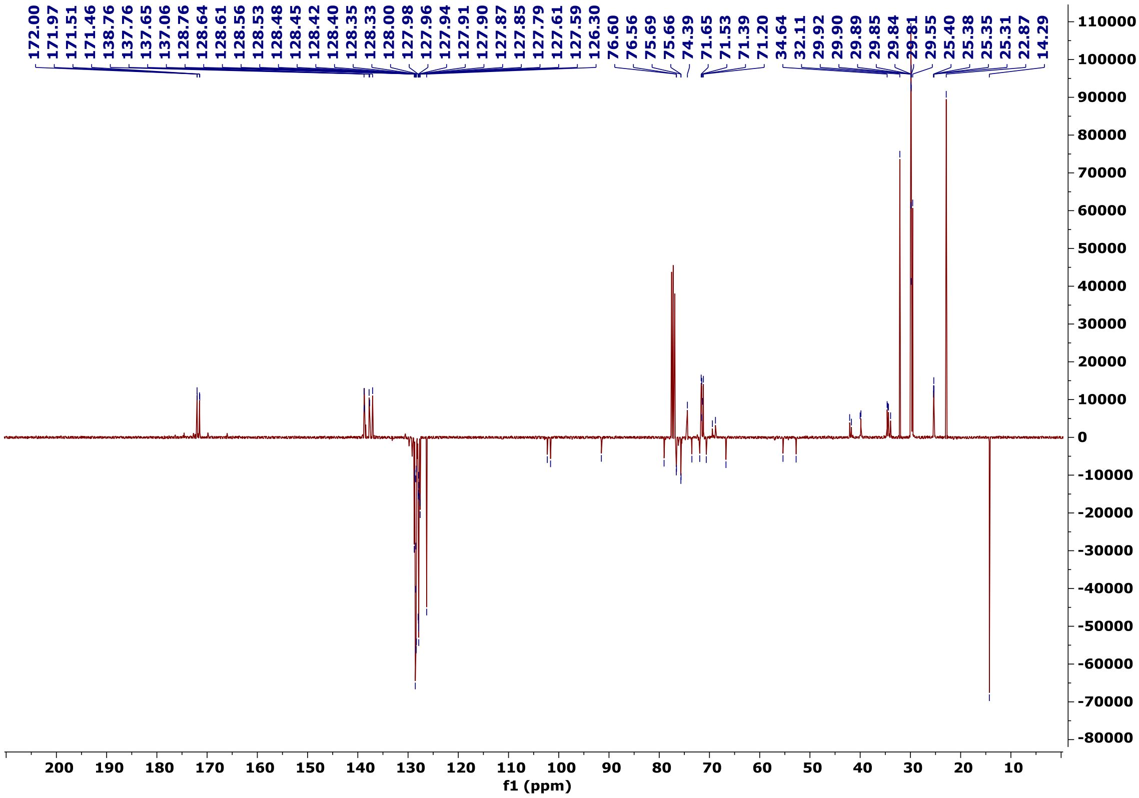

Compound 28

^{1}H-NMR (400 MHz, $CDCl_3$)

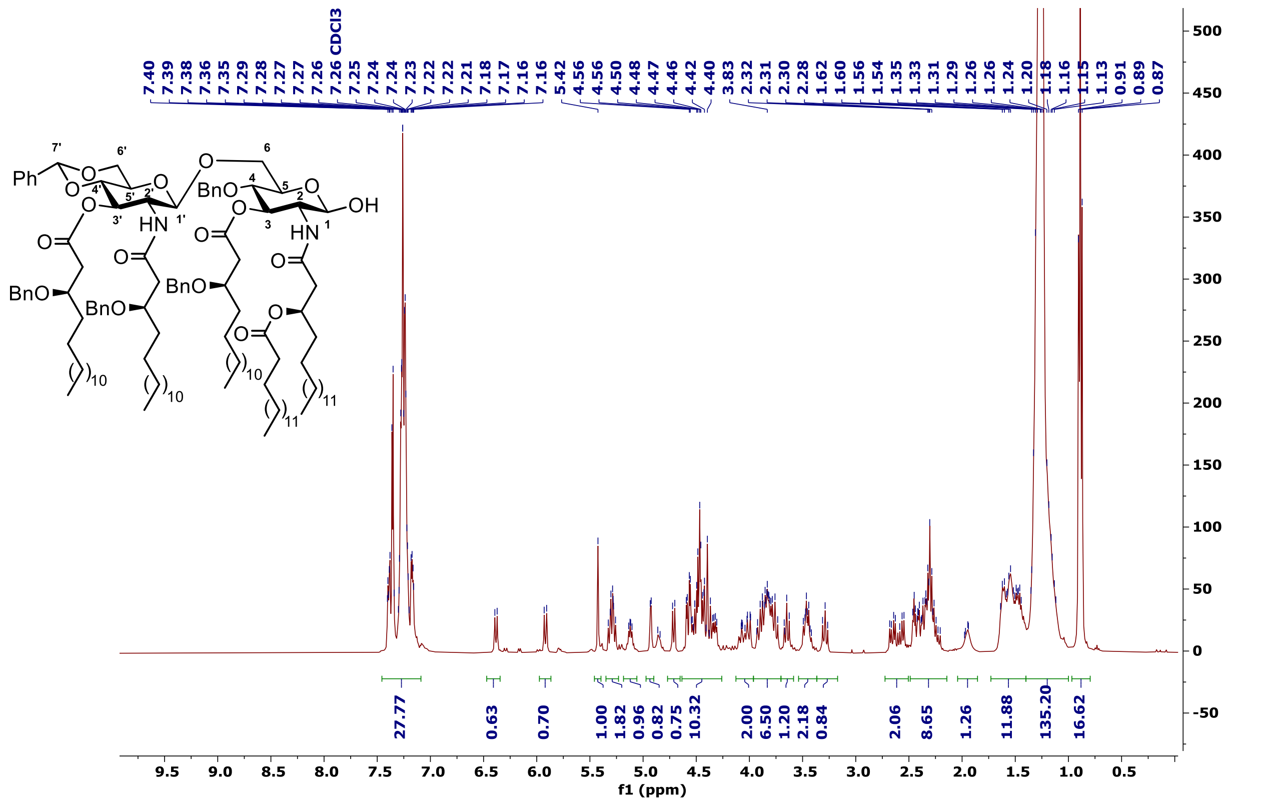

^{13}C-NMR (400 MHz, $CDCl_3$)

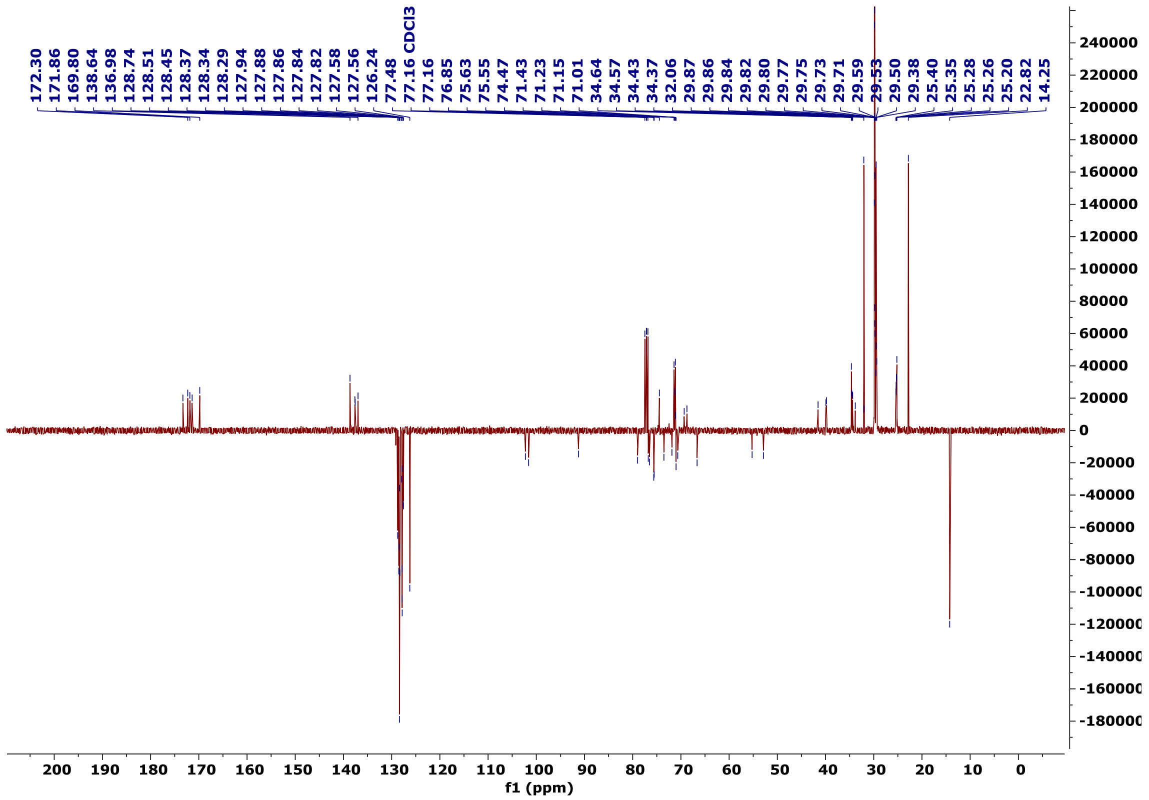

Compound 32

^{1}H-NMR (400 MHz, $CDCl_3$)

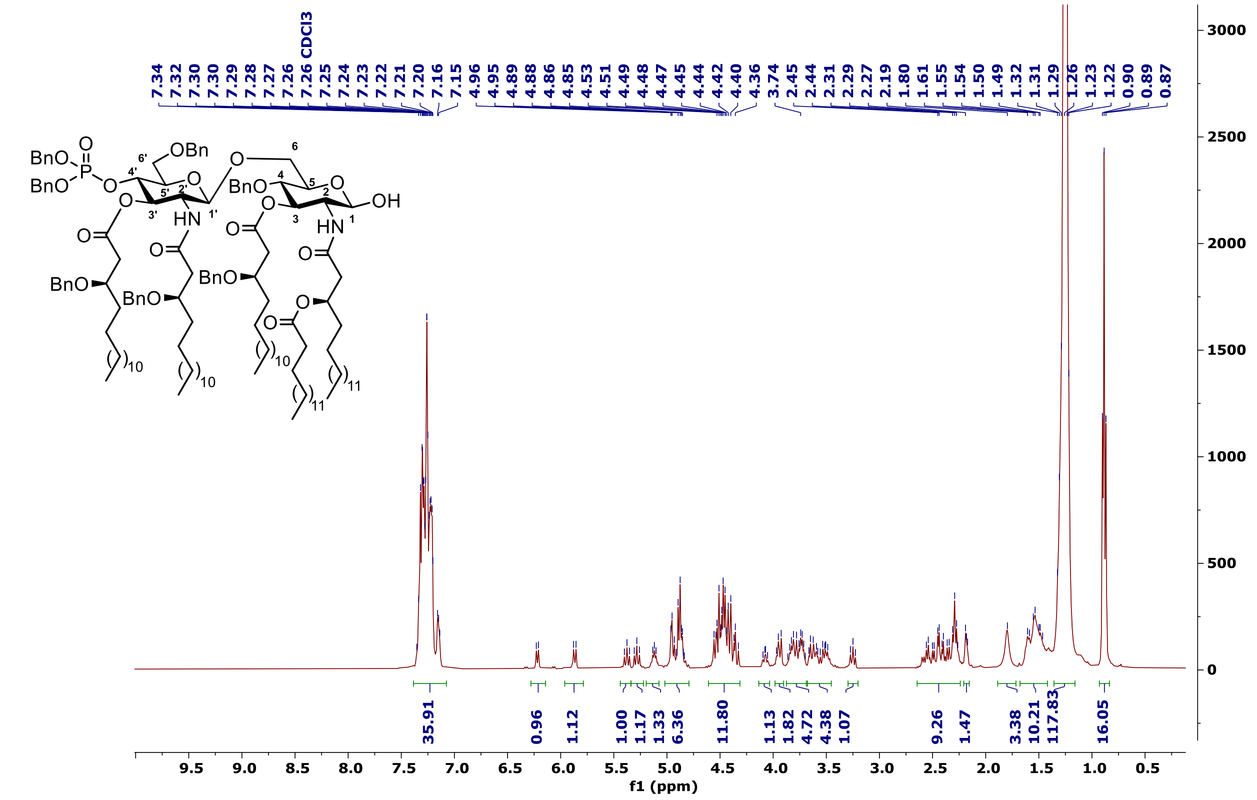

^{13}C-NMR (400 MHz, $CDCl_3$)

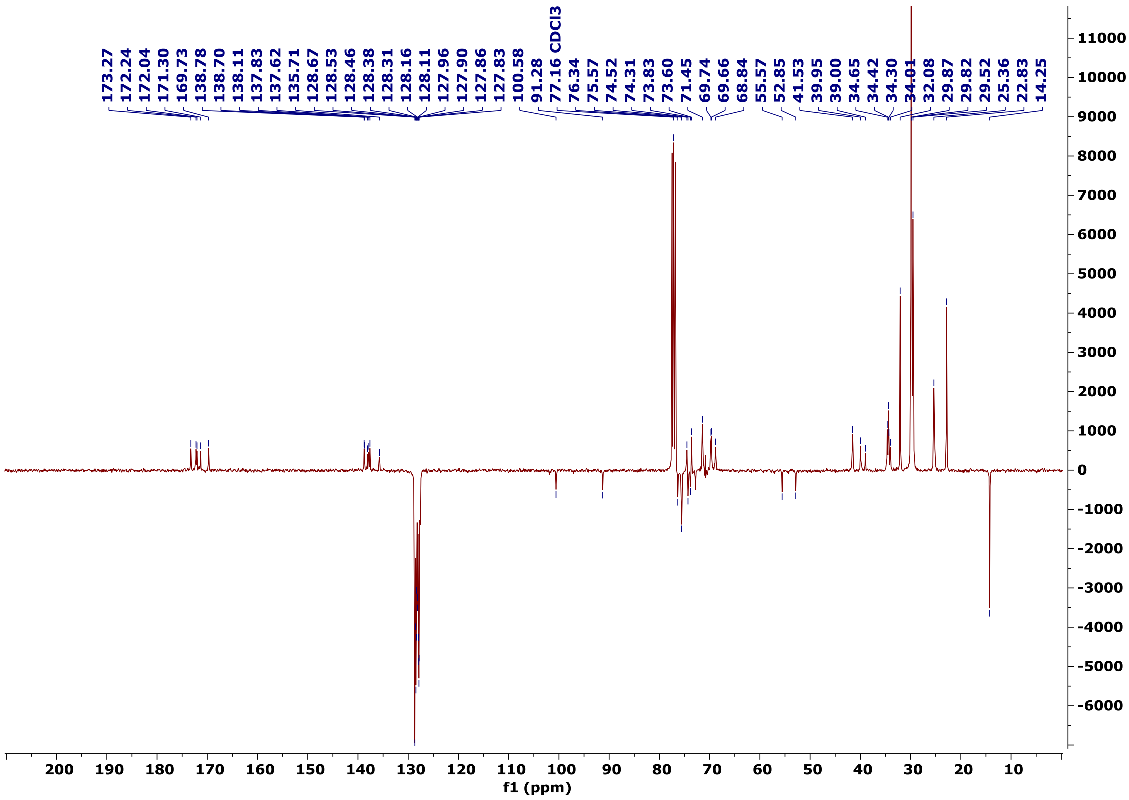

^{31}P NMR (162 MHz, $CDCl_3$)

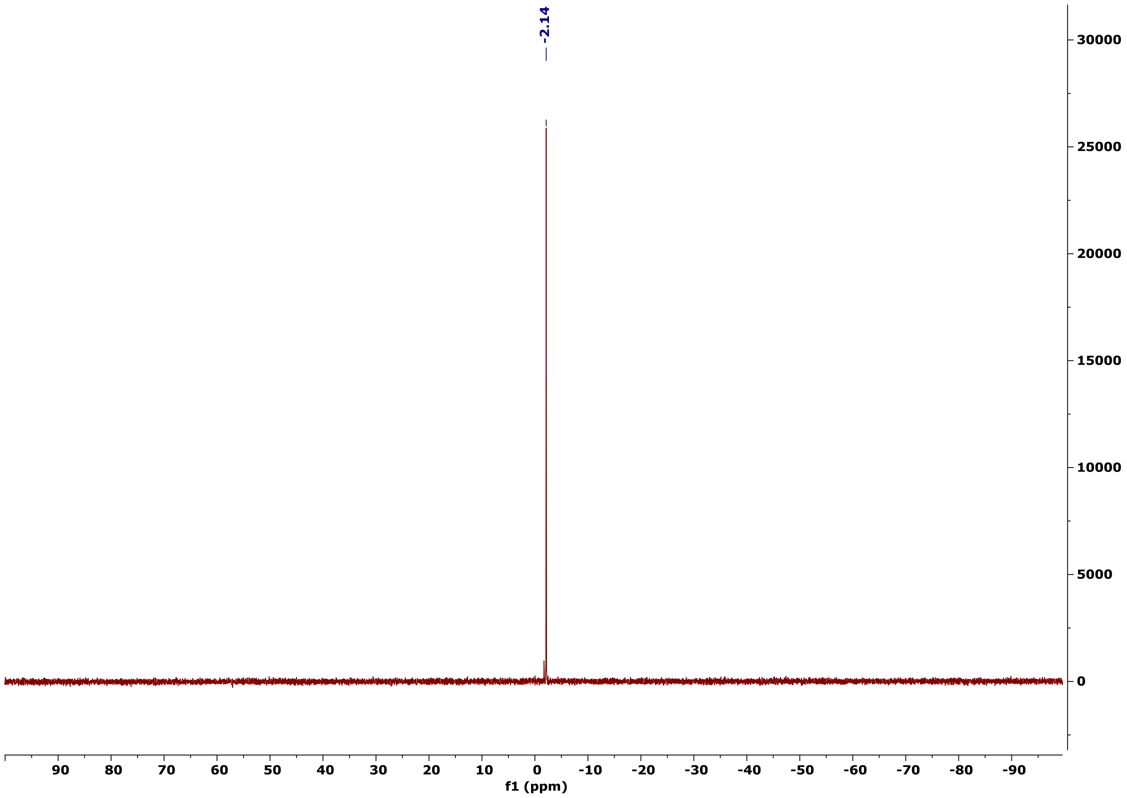

Compound 26

^{1}H-NMR (400 MHz, $CDCl_3$)

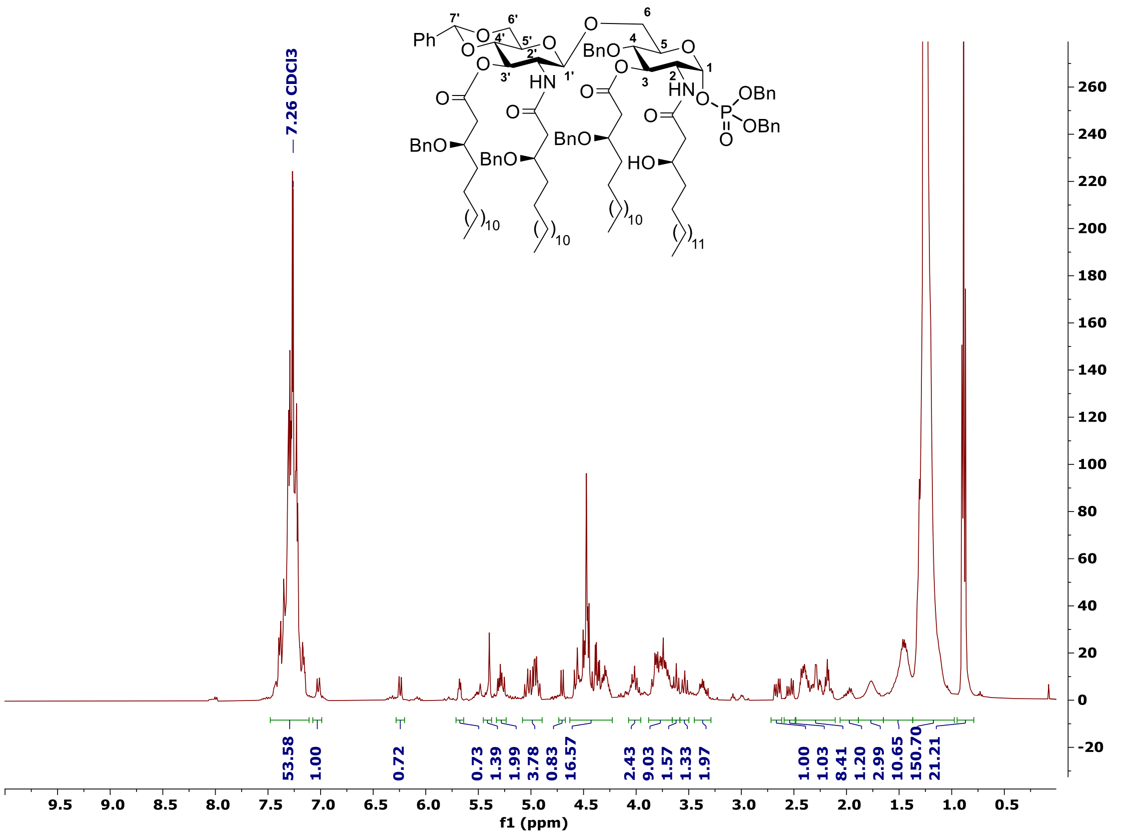

^{13}C-NMR (400 MHz, $CDCl_3$)

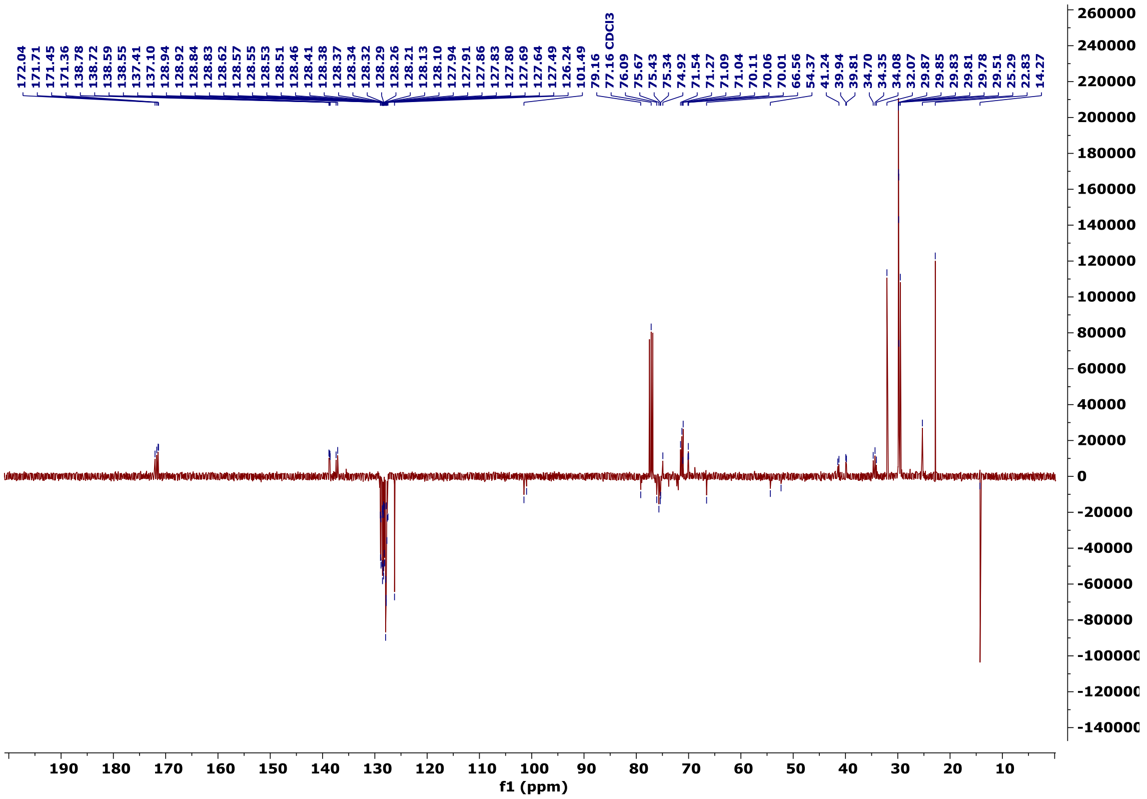

^{31}P NMR (162 MHz, $CDCl_3$)

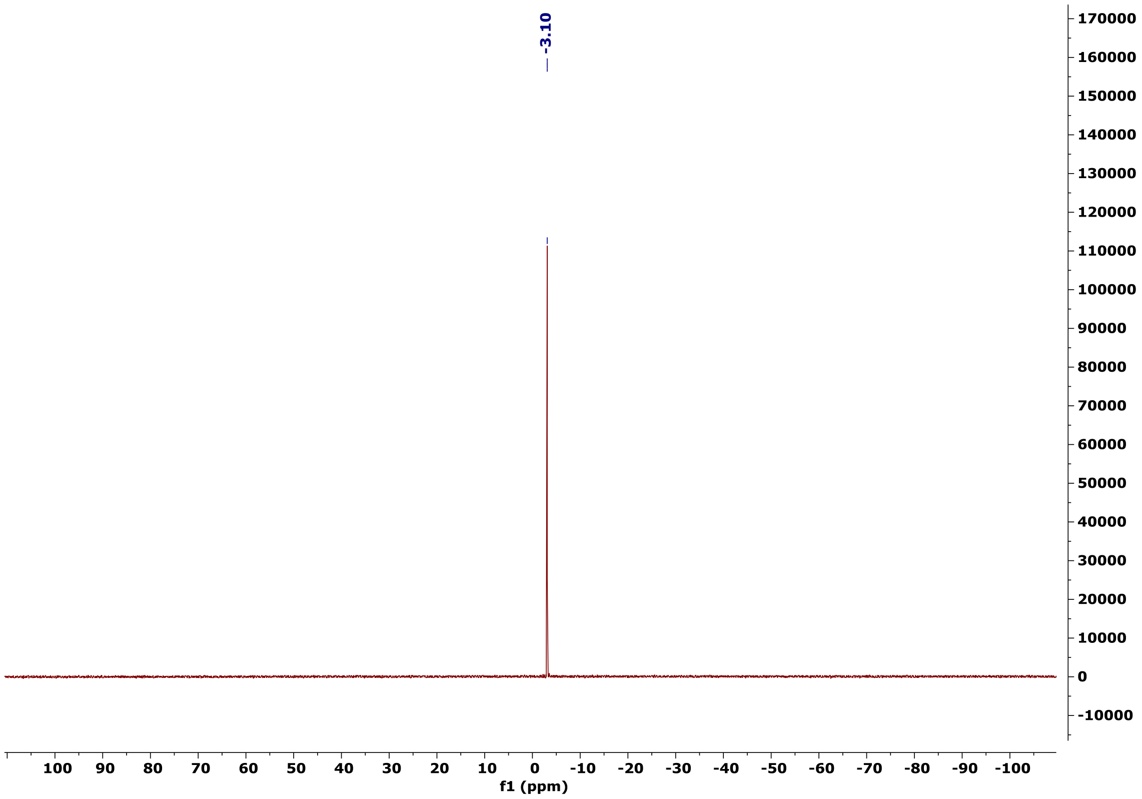

Compound 29

^{1}H-NMR (400 MHz, $CDCl_3$)

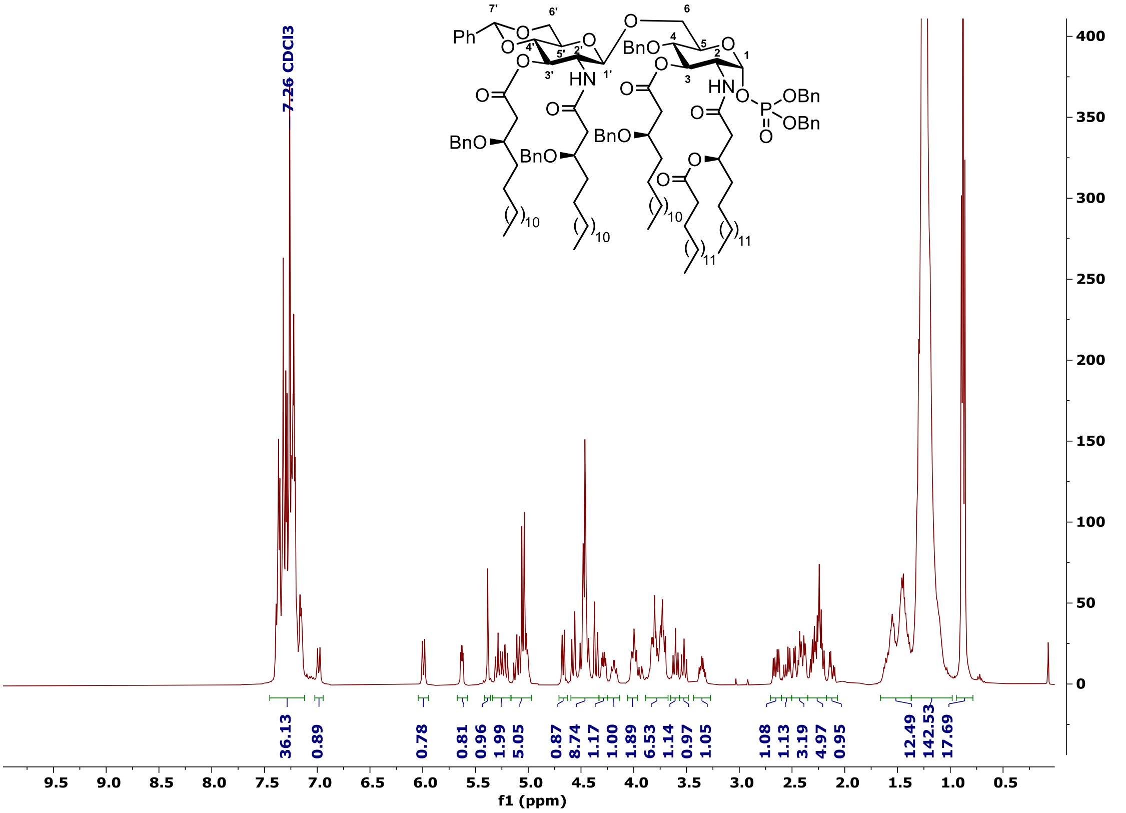

^{13}C-NMR (400 MHz, $CDCl_3$)

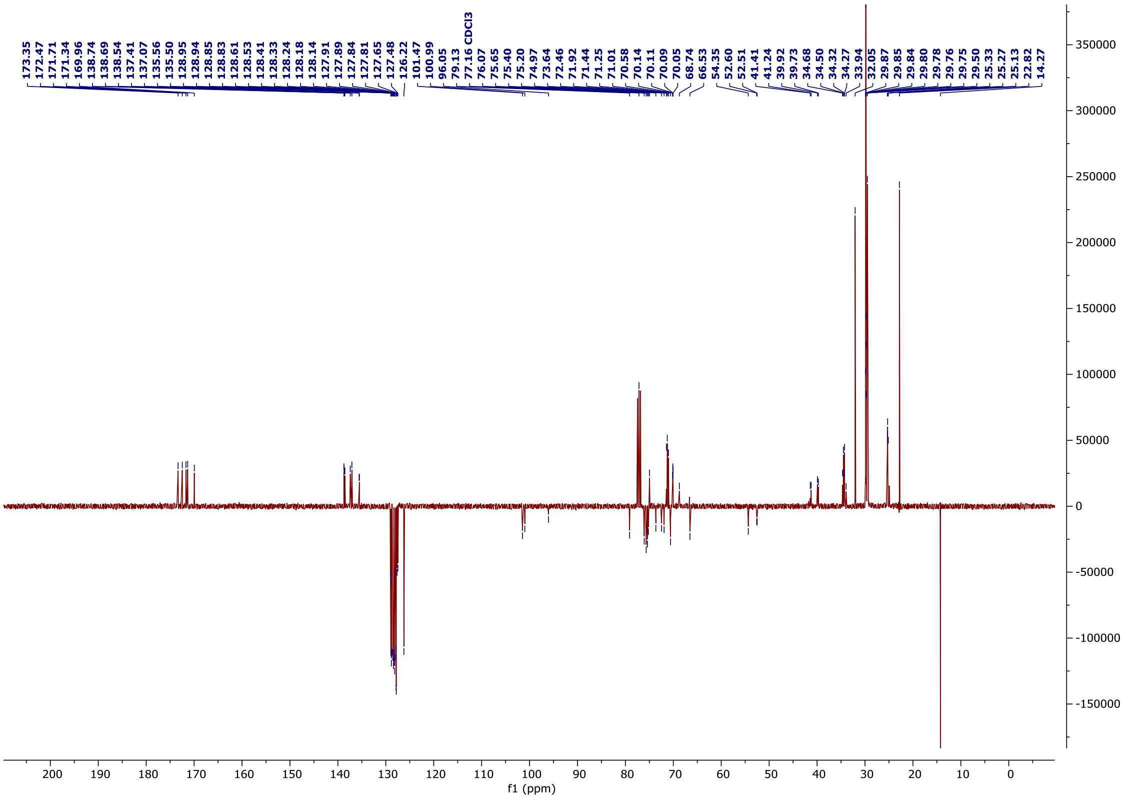

^{31}P NMR (162 MHz, $CDCl_3$)

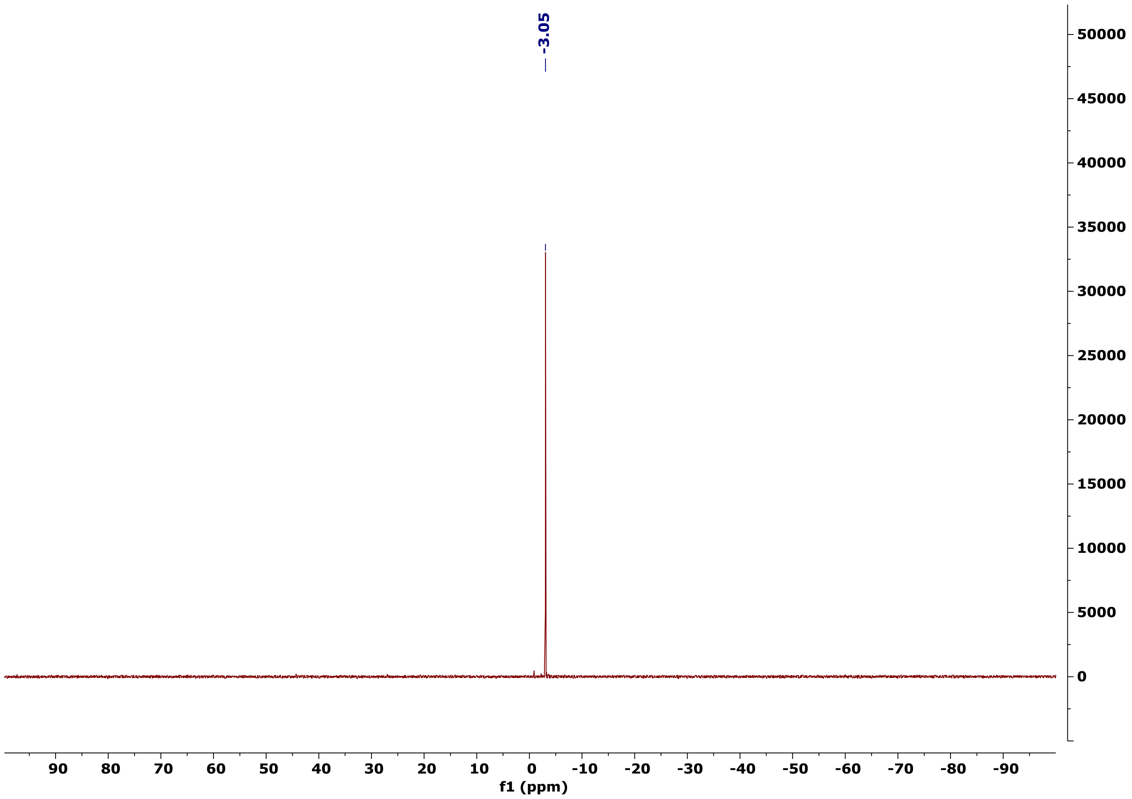

Compound 30

^{1}H-NMR (400 MHz, $CDCl_3$)

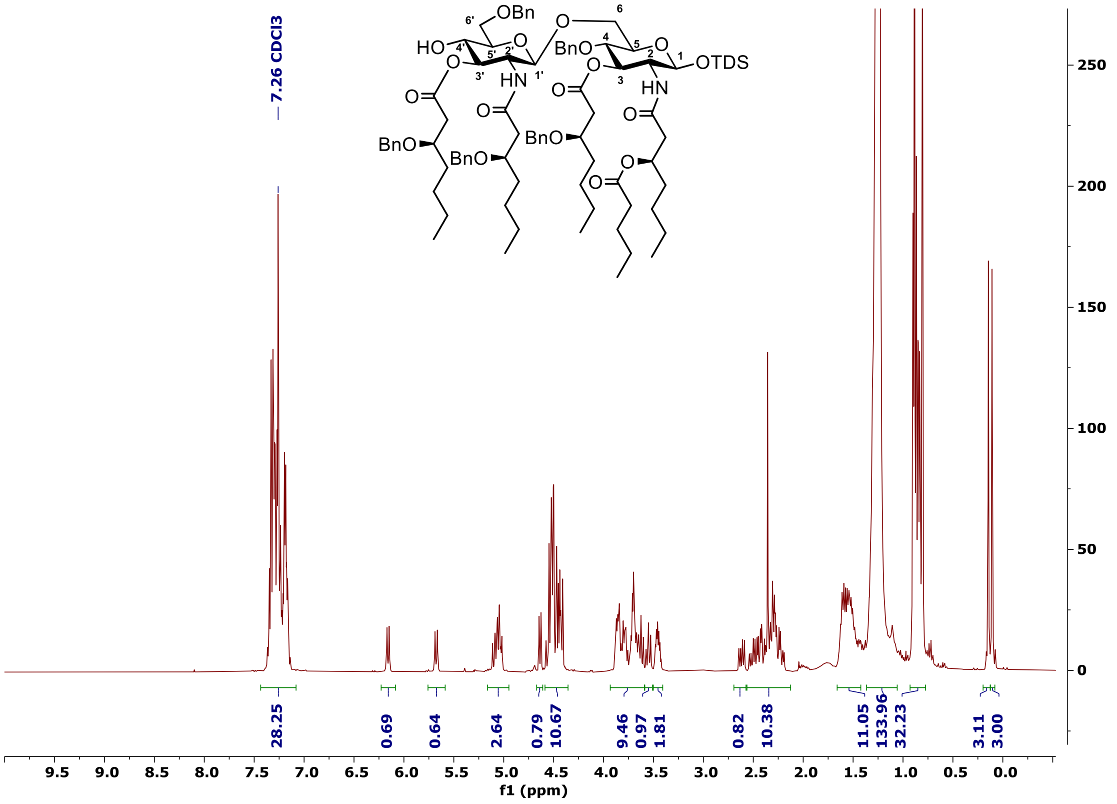

^{13}C-NMR (400 MHz, $CDCl_3$)

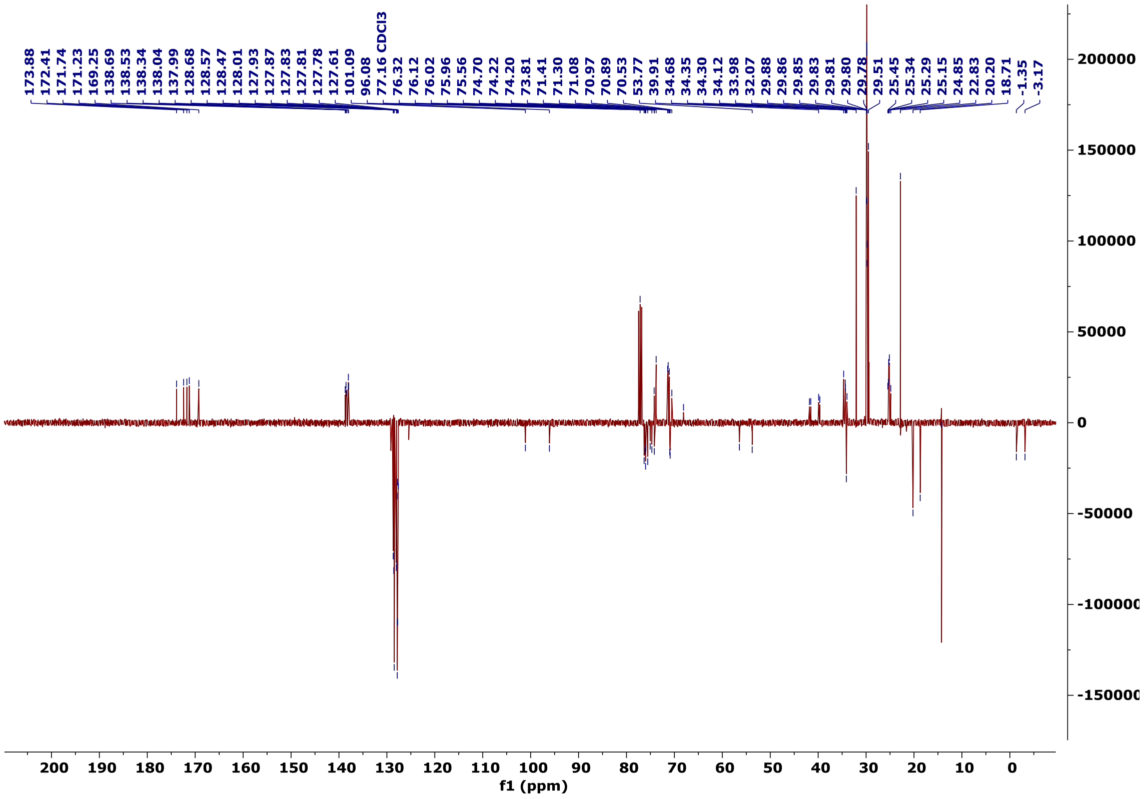

Compound 31

^{1}H-NMR (400 MHz, $CDCl_3$)

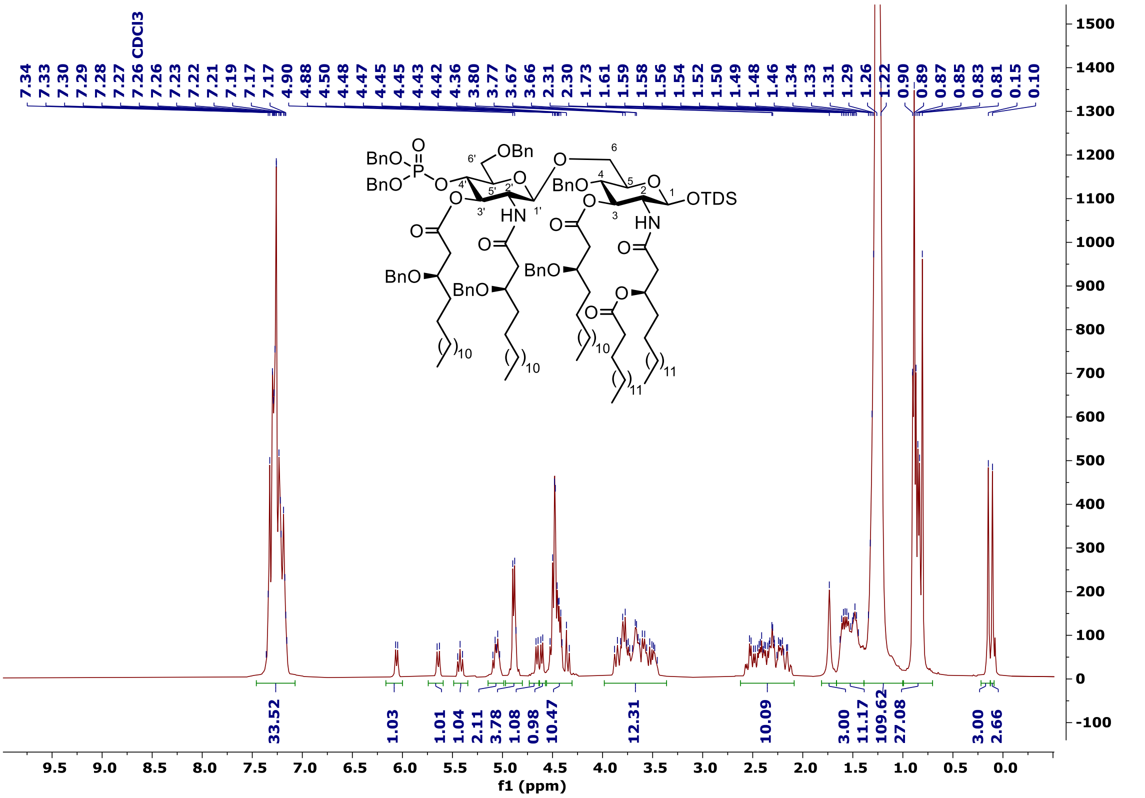

^{13}C-NMR (400 MHz, $CDCl_3$)

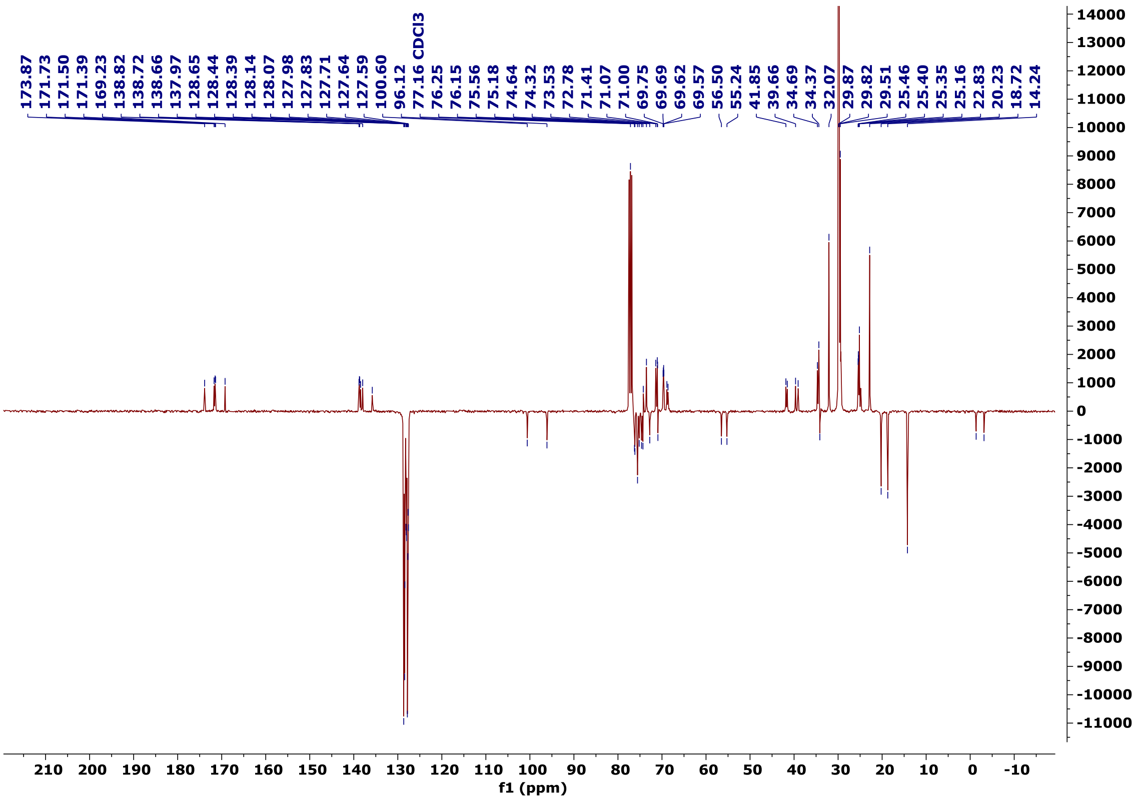

^{31}P NMR (162 MHz, $CDCl_3$)

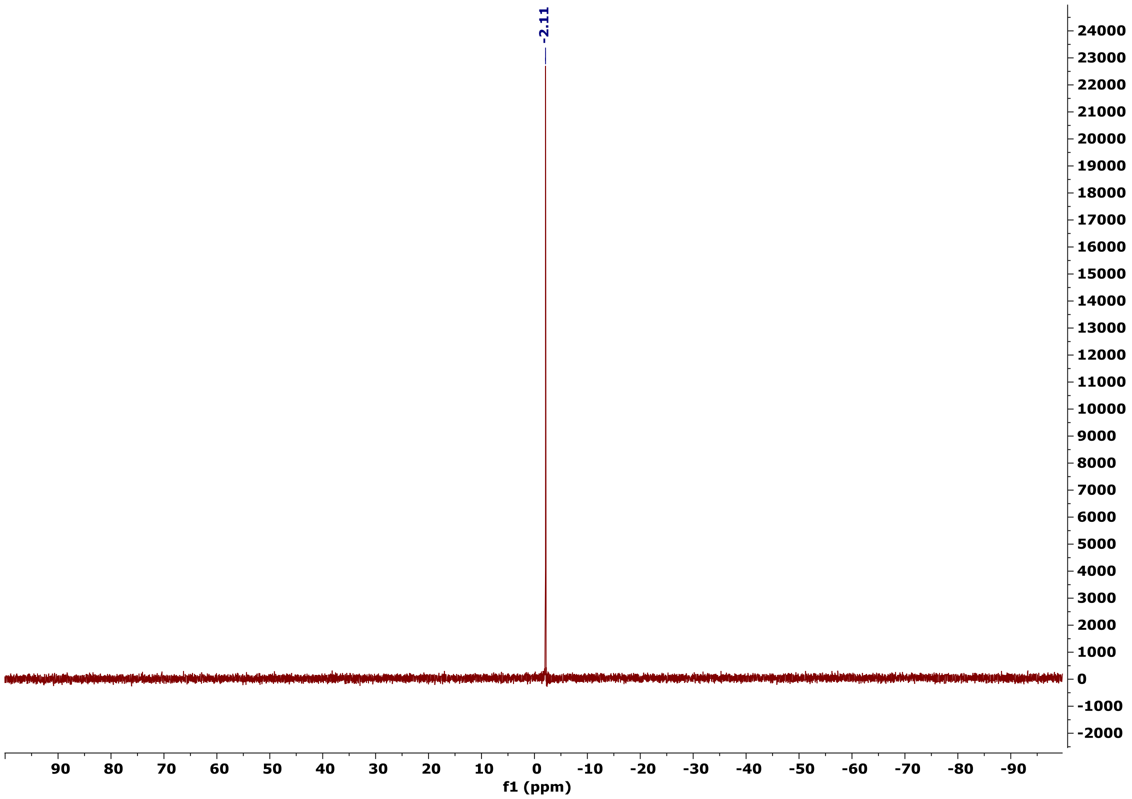

Compound Tetra-C1

^{1}H-NMR (400 MHz, $CDCl_3$/MeOD)

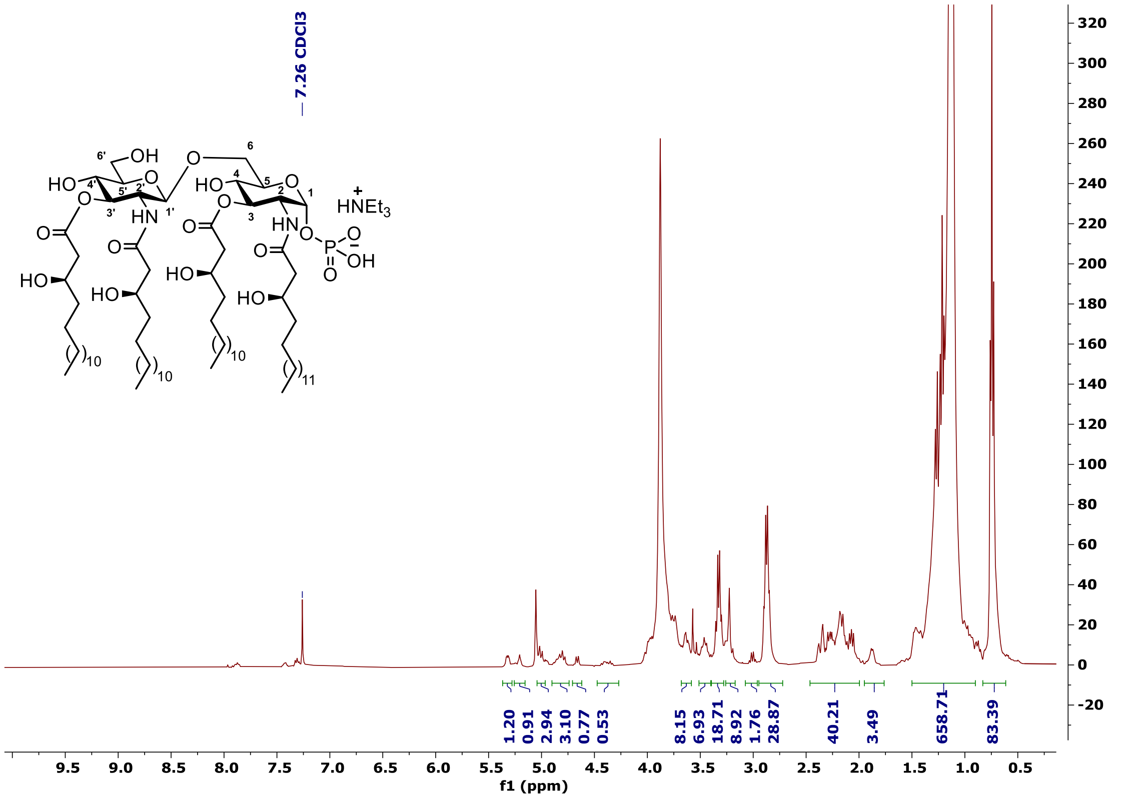

^{13}C-NMR (400 MHz, $CDCl_3$/MeOD)

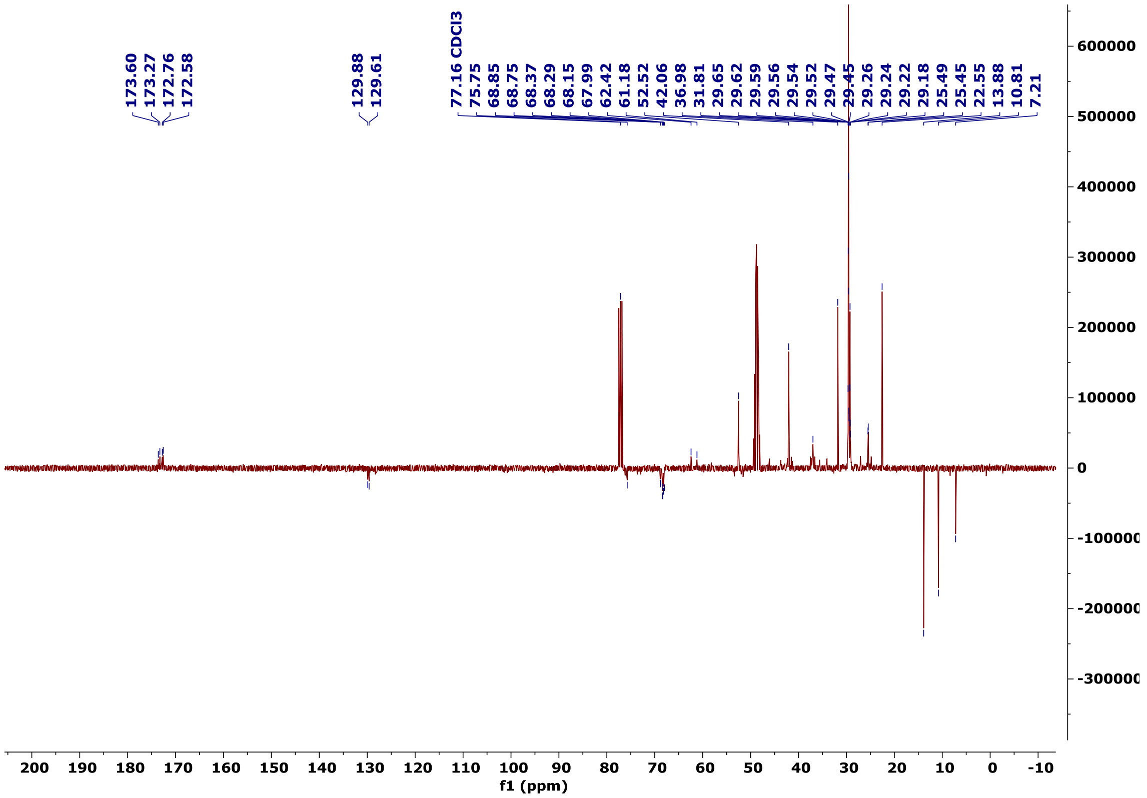

^{31}P NMR (162 MHz, $CDCl_3$/MeOD)

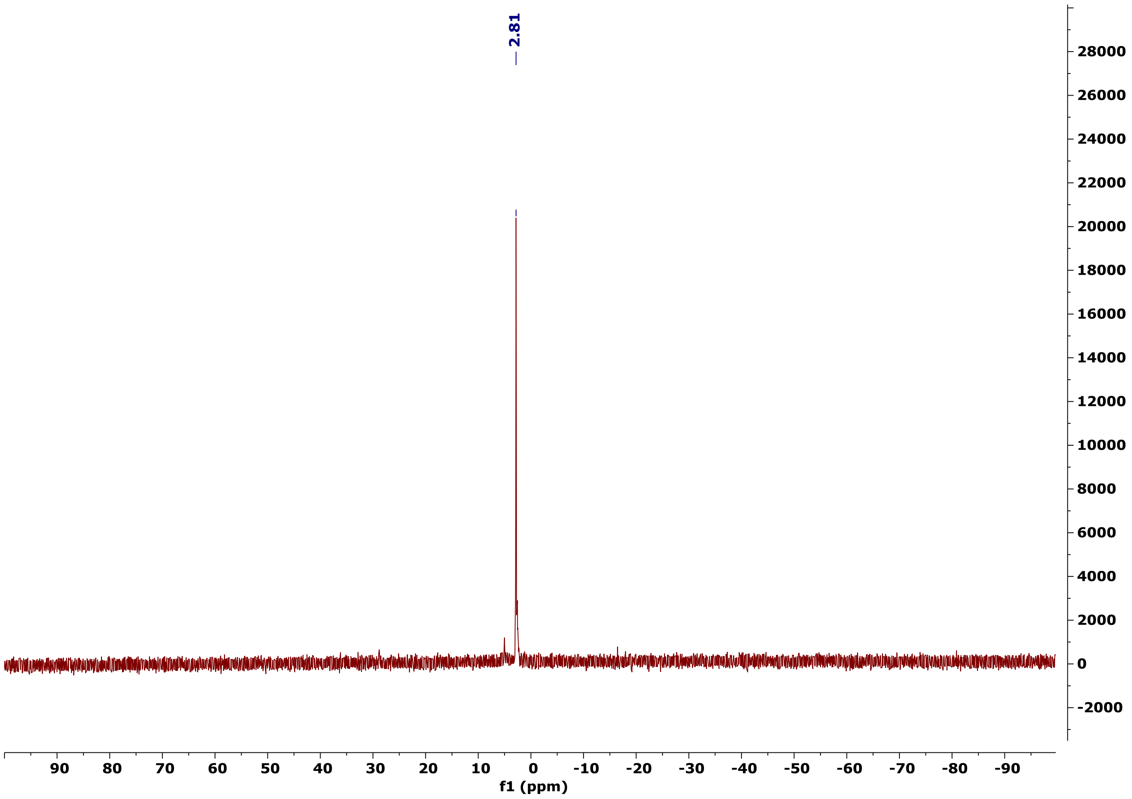

HR-MS

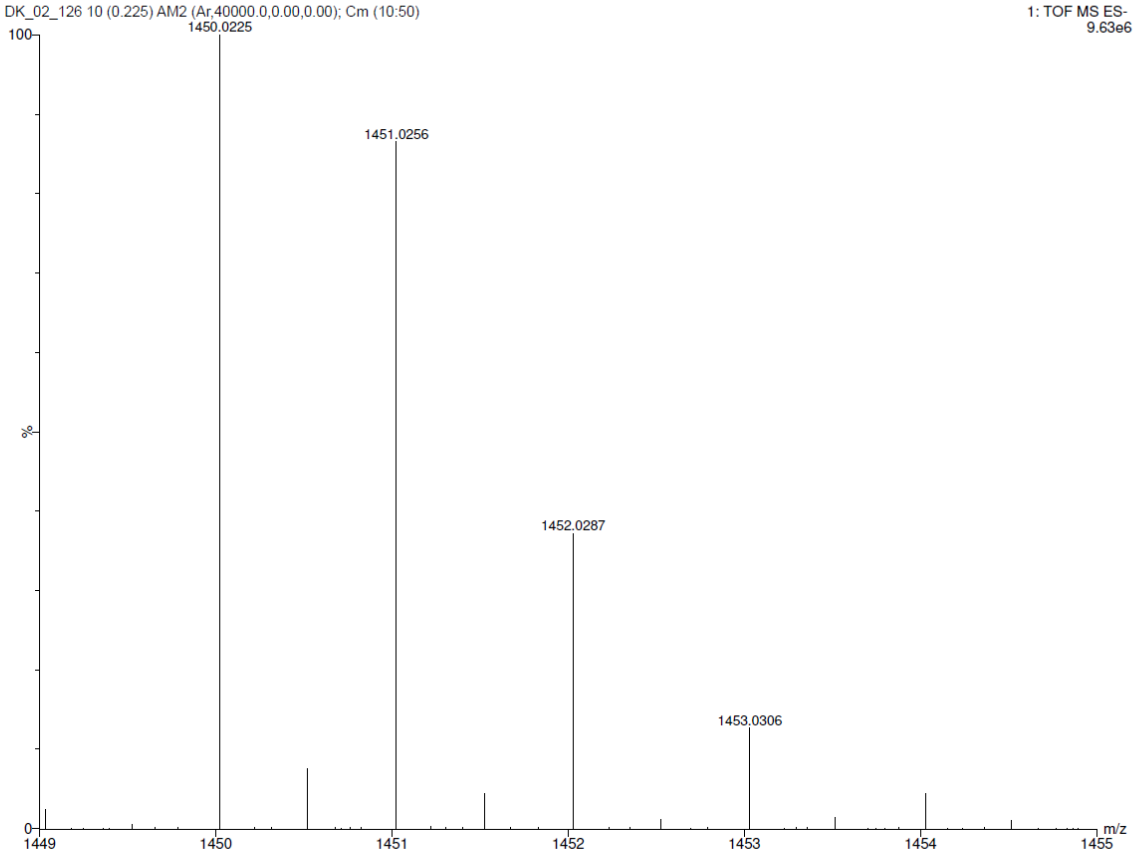

Compound Penta-C1

^{1}H-NMR (400 MHz, $CDCl_3$/MeOD)

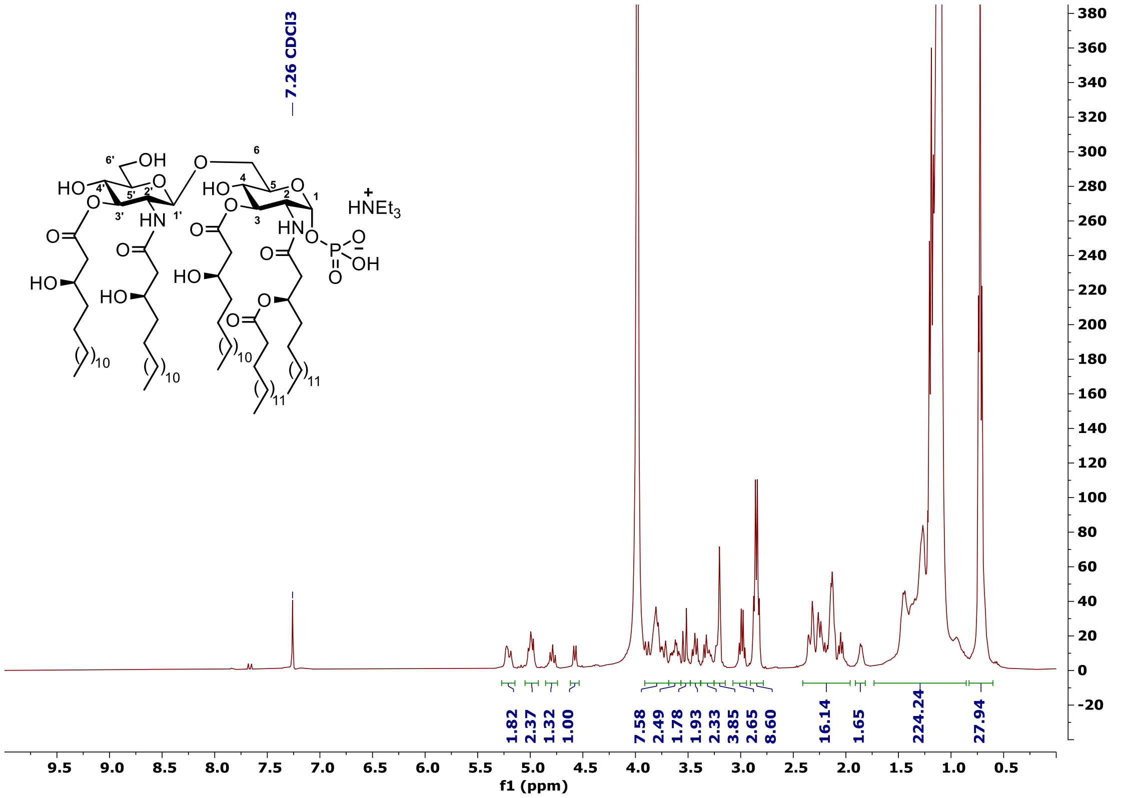

^{13}C-NMR (400 MHz, $CDCl_3$/MeOD)

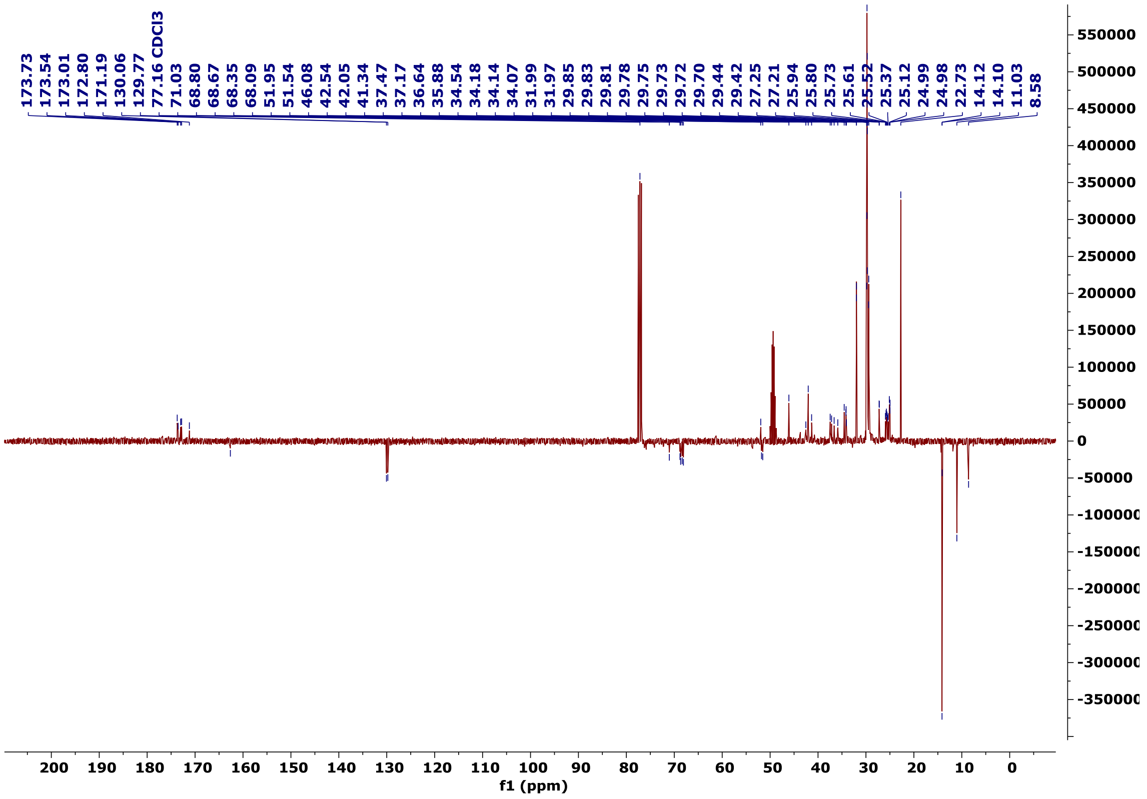

^{31}P NMR (162 MHz, $CDCl_3$/MeOD)

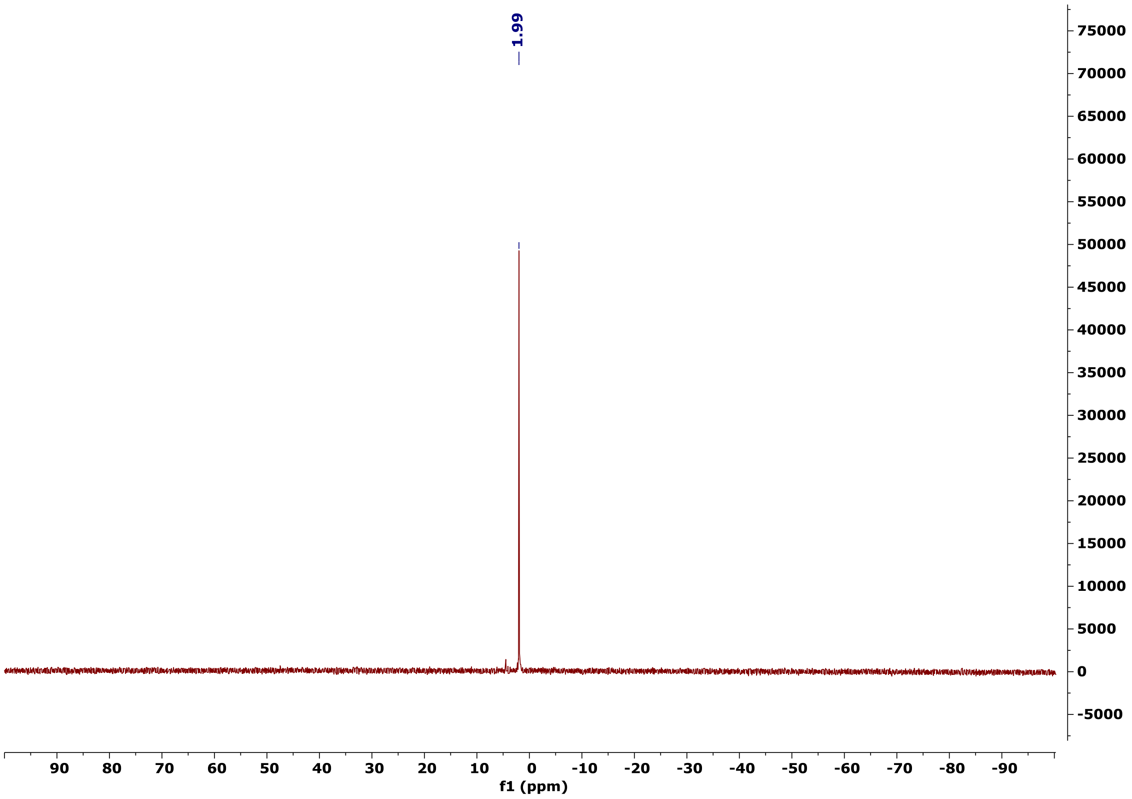

HR-MS

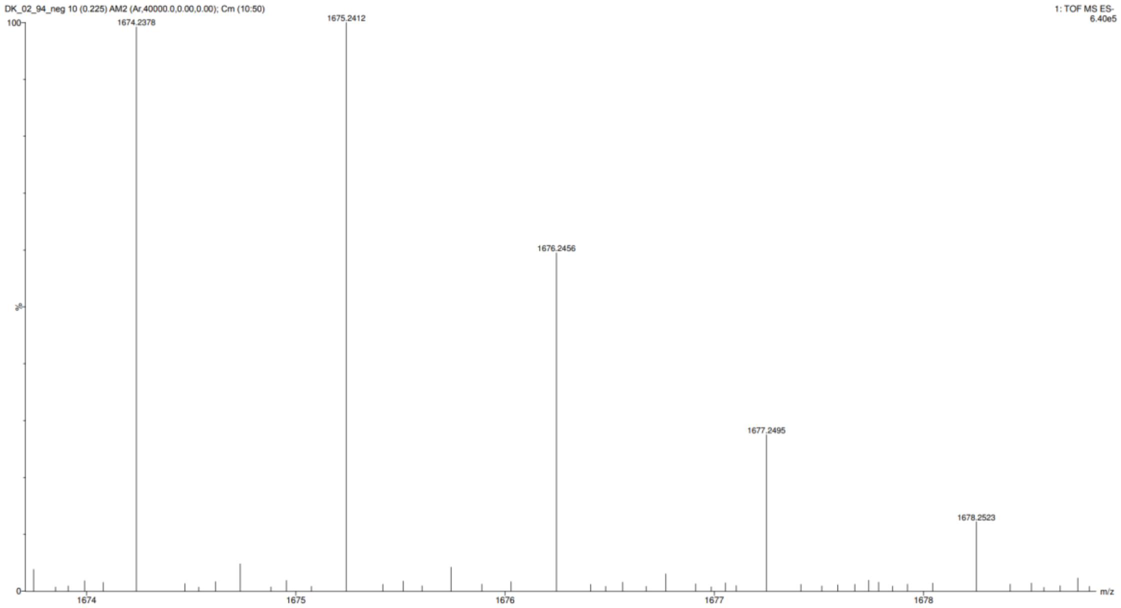

Compound Penta-C4′

^{1}H-NMR (400 MHz, $CDCl_3$/MeOD)

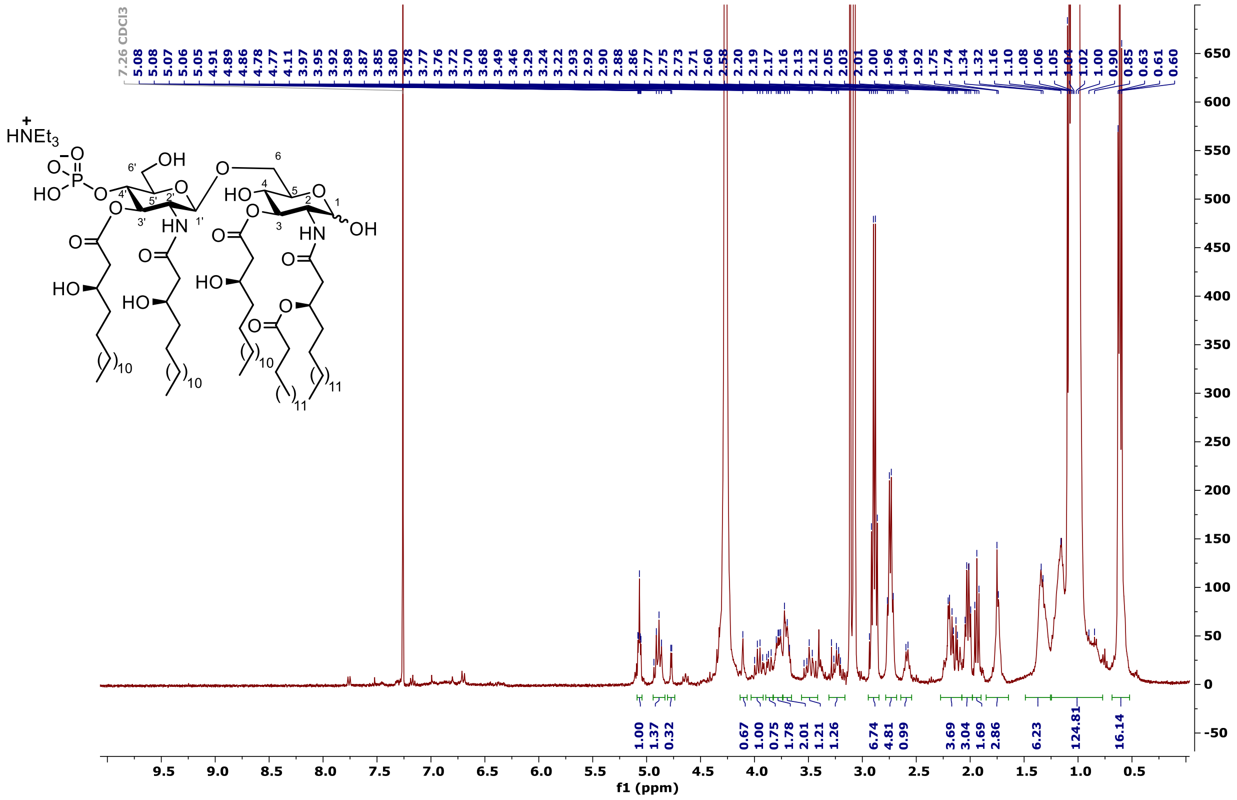

^{13}C-NMR (400 MHz, $CDCl_3$/MeOD)

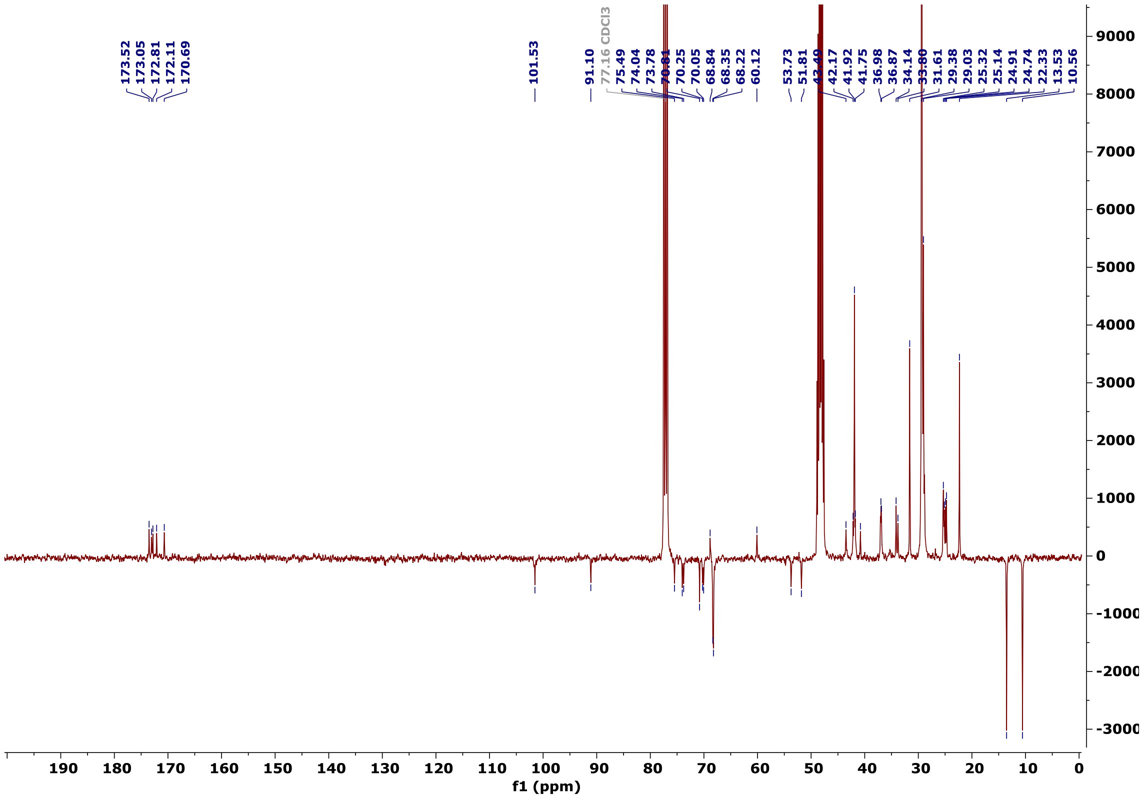

^{31}P NMR (162 MHz, $CDCl_3$/MeOD)

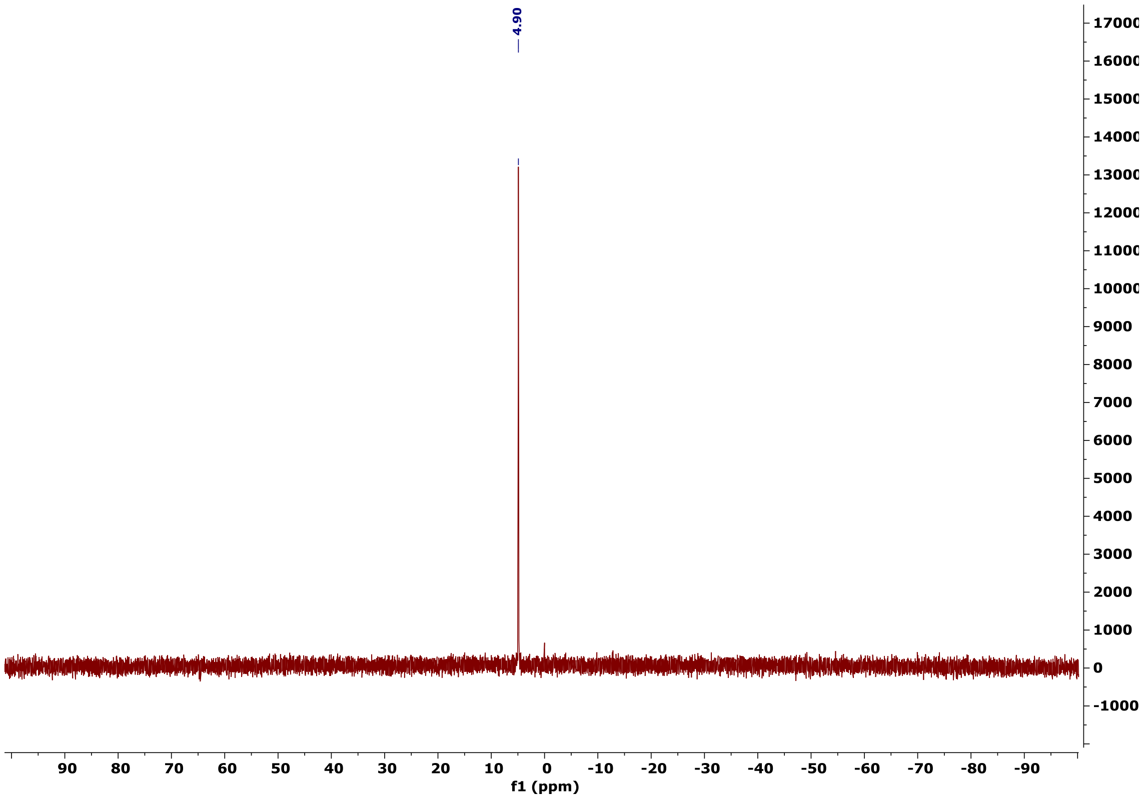

HR-MS

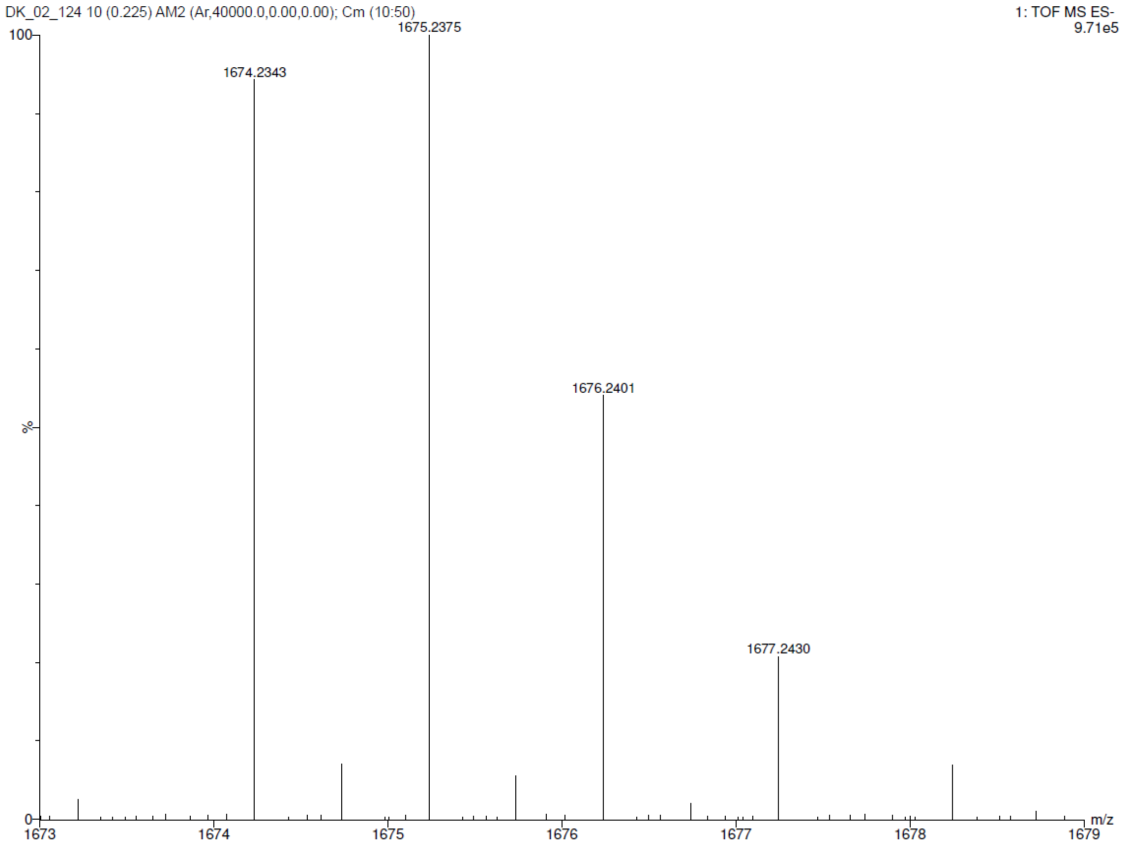

References

1. Morelli, L.; Lay, L. Synthesis of Neisseria Meningitidis X Capsular Polysaccharide Fragments. *Arkivoc* **2012**, *2013*, 166–184, doi:10.3998/ark.5550190.0014.214.
2. Melnyk, J.E.; Mohanan, V.; Schaefer, A.K.; Hou, C.-W.; Grimes, C.L. Peptidoglycan Modifications Tune the Stability and Function of the Innate Immune Receptor Nod2. *J Am Chem Soc* **2015**, *137*, 6987–6990, doi:10.1021/jacs.5b01607.
3. Liu, R.; Wei, A. Solid-Phase Synthesis of 2-Aminoethyl Glucosamine Sulfoforms. *J Carbohydr Chem* **2012**, *31*, 384–419, doi:10.1080/07328303.2012.658274.
4. Sieben, D.; Santana, A.; Nowka, P.; Weber, S.; Funke, K.; Hüttenhain, S.H. Preparation of the Even-Numbered 3-Oxo Fatty Acid Nicotinyl Esters from C6:0 to C18:0. *Tetrahedron Lett* **2016**, *57*, 808–810, doi:10.1016/j.tetlet.2016.01.031.
5. Ratovelomanana-Vidal, V.; Girard, C.; Touati, R.; Tranchier, J.P.; Hassine, B.B.; Genêt, J.P. Enantioselective Hydrogenation of Β-Keto Esters Using Chiral Diphosphine-Ruthenium Complexes: Optimization for Academic and Industrial Purposes and Synthetic Applications. *Adv Synth Catal* **2003**, *345*, 261–274, doi:10.1002/adsc.200390021.
6. Hoye, T.R.; Jeffrey, C.S.; Shao, F. Mosher Ester Analysis for the Determination of Absolute Configuration of Stereogenic (Chiral) Carbinol Carbons. *Nat Protoc* **2007**, *2*, 2451–2458, doi:10.1038/nprot.2007.354.
7. Ghosh, I.; Zeng, H.; Kishi, Y. Application of Chiral Lanthanide Shift Reagents for Assignment of Absolute Configuration of Alcohols. *Org Lett* **2004**, *6*, 4715–4718, doi:10.1021/ol048061f.
8. Verpalen, E.C.J.M.; Ehlers, A.M.; van Wingaarden, A.C.A.; Brouwer, A.J.; Boons, G.-J. Synthesis and Biological Evaluation of Lipid A Derived from Commensal *Bacteroides*. *Org Biomol Chem* **2024**, *22*, 8793–8800, doi:10.1039/D4OB01340A.
